# Evolution of canonical circadian clock genes underlies unique sleep strategies of marine mammals for secondary aquatic adaptation

**DOI:** 10.1371/journal.pgen.1011598

**Published:** 2025-03-18

**Authors:** Daiqing Yin, Zhaomin Zhong, Fan Zeng, Zhikang Xu, Jing Li, Wenhua Ren, Guang Yang, Han Wang, Shixia Xu

**Affiliations:** 1 Jiangsu Key Laboratory for Biodiversity and Biotechnology, College of Life Sciences, Nanjing Normal University, Nanjing, China; 2 Southern Marine Science and Engineering Guangdong Laboratory (Guangzhou), Guangzhou, Guangdong, China; 3 Center for Circadian Clocks, Soochow University, Suzhou, Jiangsu, PR China; 4 School of Biology & Basic Medical Sciences, Suzhou Medical College, Soochow University, Suzhou, Jiangsu, PR China; Charité - Universitätsmedizin Berlin, GERMANY

## Abstract

To satisfy the needs of sleeping underwater, marine mammals, including cetaceans, sirenians, and pinnipeds, have evolved an unusual form of sleep, known as unihemispheric slow-wave sleep (USWS), in which one brain hemisphere is asleep while the other is awake. All aquatic cetaceans have only evolved USWS without rapid eye movement (REM) sleep, whereas aquatic sirenians and amphibious pinnipeds display both bihemispheric slow-wave sleep (BSWS) and USWS, as well as REM sleep. However, the molecular genetic changes underlying USWS remain unknown. The present study investigated the evolution of eight canonical circadian genes and found that positive selection occurred mainly within cetacean lineages. Furthermore, convergent evolution was observed in lineages with USWS at three circadian clock genes. Remarkably, *in vitro* assays showed that cetacean-specific mutations increased the nuclear localization of zebrafish *clocka*, and enhanced the transcriptional activation activity of Clocka and Bmal1a. *In vivo*, transcriptome analysis showed that the overexpression of the cetacean-specific mutant *clocka* (*clocka*-mut) caused the upregulation of the wakefulness-promoting glutamatergic genes and the differential expression of multiple genes associated with sleep regulation. In contrast, the GABAergic and cholinergic pathways, which play important roles in promoting sleep, were downregulated in the *bmal1a*-mut-overexpressing zebrafish. Concordantly, sleep time of zebrafish overexpressing *clocka*-mut and *bmal1a*-mut were significantly less than the zebrafish overexpressing the wild-type genes, respectively. These findings support our hypothesis that canonical circadian clock genes may have evolved adaptively to enhance circadian regulation ability relating to sleep in cetaceans and, in turn, contribute to the formation of USWS.

## Author summary

Despite decades of intensive research, the functions and mechanisms of sleep remain elusive. What are the phylogenetic origins of sleep? What are the adaptive changes occurring across the life-time, and why would it be? These are some questions that await answers. Sleeping in the water poses a significant challenge for air-breathing mammals. Accordingly, aquatic mammals have evolved a very special form of sleep characterized by unihemispheric slow-wave sleep (USWS) and a negligible amount or complete absence of REM sleep. The existence of such a specific nature of lateralized sleep in aquatic mammals suggests the role of evolutionary adaptive forces in the development and modification of mammalian sleep. It may offer us some clues to find out the ways for sleep management and skills for the adverse effects of sleep loss experienced in humans. Here, we assessed the evolutionary characteristics of canonical circadian genes across mammalian phylogeny and found genetic changes possibly related to the generation of USWS. *In vitro* and *in vivo* assays subsequently revealed that cetacean-specific mutations in *CLOCK* and *BMAL1* play a positive role in transcriptional activation and the associated wake-promoting effects. Our findings facilitate a deeper understanding how molecular changes affect sleep states over evolutionary time.

## Introduction

Sleep, an indispensable part of animal life, refers to a state of rapidly reversible immobility with greatly reduced responsiveness to environmental stimuli [1]. Mammalian sleep displays two distinct cyclically alternating phases: slow-wave sleep (SWS, also called non-rapid eye movement sleep) and rapid eye movement (REM) sleep [2]. SWS is characterized by high-amplitude slow waves in an electroencephalogram (EEG), as well as behavioral and autonomic nervous system quiescence [3], whereas REM sleep is characterized by low-amplitude and rapid desynchronized EEG activity, episodic bursts of rapid eye movements, the suppression of muscle activity, and decreased thermoregulation [4].

During the long-term process of ecological adaptation and speciation, different mammals have evolved different sleep features. Marine mammals, a fascinating group of mammals that includes cetaceans (whales, dolphins, and porpoises), sirenians (manatees and dugongs), and pinnipeds (walrus, otariids and phocids), have made the transition from land to the ocean via their independent terrestrial ancestors [5]. To adapt to their needs of sleeping and moving in the aquatic environment, marine mammals have evolved unihemispheric slow-wave sleep (USWS), *i.e.*, where one brain hemisphere is in a state of SWS while the other is awake; this is different from bihemispheric slow-wave sleep (BSWS), found in all terrestrial mammals, in which both brain hemispheres sleeping simultaneously [1, 2]. The unique sleep process of USWS allows marine mammals not only to facilitate continuous movement but also to reap the benefits of sleep while breathing, monitoring the surroundings, and thermogenesis underwater [6]. The sleep of fully aquatic cetaceans, is characterized by USWS, along with continuous swimming and the absence or a negligible amount of REM sleep [7]. However, sirenians, another type of fully aquatic mammals, display not only the typical features of BSWS and REM sleep but also USWS, and maintain a motionless state at the bottom of the water during all sleep stages [8]. In the case of amphibious pinnipeds, including three families, Odobenidae (walrus), Otariidae (sea lions and fur seals), and Phocidae (true seals), the Odobenidae and Otariidae families predominantly exhibit a typical BSWS and REM sleep found in all terrestrial mammals, and USWS as in cetaceans [9]. In contrast, the Phocidae family display BSWS and REM sleep distinguished by EEG recordings, but the complete absence of USWS both on land and in water; they hold their breath when sleeping under water and periodically awaken to return to the surface to breathe [10].

It is proposed that sleep is regulated by two separate biological mechanisms: circadian rhythm and sleep-wake homeostasis [11]. The circadian clock is the main determinant of the distribution of sleep over a 24-hour period [12]. In mammals, the circadian system involves an autoregulatory transcriptional feedback loop that contains eight core genes encoding CLOCK (Circadian Locomotor Output Cycles Kaput Protein) and its paralog NPAS2 (neuronal PAS domain protein 2); BMAL1 (Brain and Muscle ARNT-like protein 1); CRY1 (CRYPTOCHROME 1) and CRY2 (CRYPTOCHROME 2); and PER1 (PERIOD 1), PER2 (PERIOD 2) and PER3 (PERIOD 3), respectively [13, 14]. CLOCK and BMAL1 proteins form heterodimers that bind to E-boxes and drive the expression of clock-controlled genes [15]. NPAS2 and BMAL1 proteins can also form heterodimers and induce gene expression through E-boxes [16, 17]. CLOCK:BMAL1 or NPAS2:BMAL1 heterodimers drive the transcription of three *PERIOD* and two *CRYPTOCHROME* genes [18, 19]. PER and CRY proteins, in turn, form complexes in the cytoplasm, and at a certain threshold, the PER/CRY complex translocates into the nucleus, where it represses its own transcription by interaction with CLOCK and BMAL1 [20, 21]. When PER:CRY complexes are eventually degraded in a controlled fashion, this inhibition is relieved, and CLOCK:BMAL1 heterodimers become active again, allowing the feedback loop to restart again [22, 23]. Some sleep disorders are closely related to genetic variation in circadian clock genes [24]. By contrast, the homeostasis system controls the increasing need for sleep during wakefulness and the decrease during sleep [25]. Sleep homeostasis involves multiple neurotransmitters, for example, glutamate is an important wake-promoting factor, whereas gamma-aminobutyric acid (GABA) and acetylcholine (ACh) are sleep-promoting factors [26]. In addition, there is growing experimental evidence that circadian clock genes have a direct effect on sleep homeostatic processes [27].

Unihemispheric sleep in marine mammals has been examined from electrophysiological and ecological perspectives; however, the underlying molecular mechanism of USWS remains poorly explored. Studies demonstrate the close functional and evolutionary relationships between sleep and circadian rhythms [28], as sleep behaviors are typically accompanied by the circadian organization [29]. Furthermore, genetic changes in the circadian network are found to be related to interspecies sleep diversity. For example, cave populations of the Mexican tetra (*Astyanax mexicanus*) have evolved a dramatically reduced sleep phenotype [30]. Widespread dysregulation of the circadian transcriptome plays an essential role in the evolved sleep loss in cave populations [31]. Moreover, the rapid evolution of *PER1* and premature termination of *PER2* are assumed to promote short and fragmented sleep patterns in giraffe (*Giraffa camelopardalis rothschildi*) [32]. In this study, we investigate the evolutionary history of eight canonical circadian clock genes in mammals to determine whether adaptive signatures were restricted to unihemispheric sleepers and whether marine mammals could provide molecular evidence for the convergent evolution of USWS. To explore the possible genetic basis of USWS in cetaceans, we screened for cetacean-specific mutations in these eight circadian clock genes via sequence comparison with other mammals, and further evaluated the potential functional consequences of identified mutations in two critical positive elements, CLOCK and BMAL1, by performing *in vitro* and *in vivo* assays using the zebrafish (*Danio rerio*), a well-established vertebrate model in sleep research [33]. This model species exhibits established, behaviorally defined diurnal sleep, and bears genetic and neuroanatomical similarities to mammalian sleep [34]. Furthermore, zebrafish offers unique advantages over more commonly used rodent models, including rapid development with complex behaviors such as sleep present in 5-day-old animals, and amenability to high-throughput behavioral assays and to genetic manipulations [33]. Overall, our results can help to elucidate the genetic mechanisms determining the unique USWS found in marine mammals and, in general, the associated adaptations.

## Results

**Fig 1:**
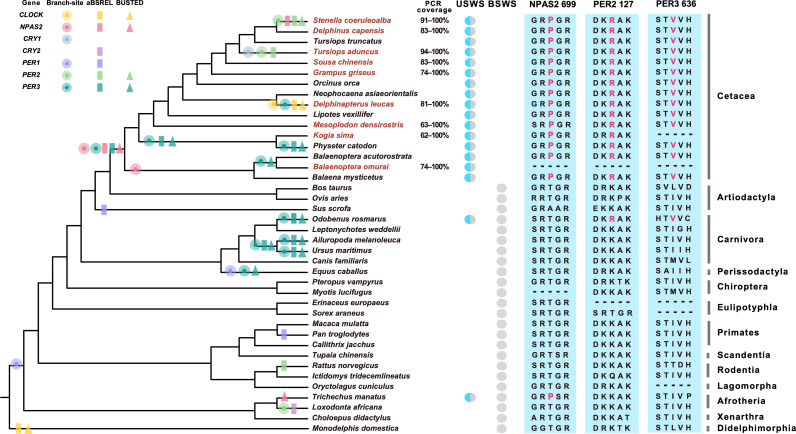
Evidence for positive selection and convergence sites across mammalian phylogeny. Positively selected lineages identified by the branch-site model, aBSREL, and BUSTED are indicated separately by circle, rectangle, and triangle. Cetacean species used to sequence circadian genes were marked with brown text. The unique mutations shared by USWS species are shown in the right of the figure and highlighted in red. Amino acid locations were deduced with reference to the human gene.

We successfully sequenced eight circadian clock genes in nine representative species of cetaceans, and newly obtained sequences (GenBank accession numbers OR712815–OR712886) covered at least 62% of the coding sequences (CDS) (Fig 1, S1 Table). All genes were intact and there were no frame-shift mutations or premature stop codons, which indicated the presence of functional proteins in cetaceans. The orthologous sequences of canonical circadian genes were also downloaded from 31 other mammals, including 11 marine mammals and 20 terrestrial relatives. Thus, these eight circadian clock genes from 38–40 species from representative mammalian lineages were used for our subsequent analyses (S2 Table). The overall evolutionary distance between amino acid sequences for eight circadian clock genes was estimated to be 0.02 to 0.31, and among them, the *BMAL1* gene showed the highest sequence similarity (a range of 93.57 - 100.00%) whereas the *PER3* gene showed the lowest similarity ranged from 40.87 to 99.42% (S3 Table).

### Evidence for positive selection of canonical circadian genes in marine mammals

The branch-site model in phylogenetic analysis by maximum likelihood (PAML) [35] and two methods in HyPhy [36] were used to determine whether positive selection occurred within specific mammalian lineages. The branch-site model revealed that the terminal branch of the beluga whale (*Delphinapterus leucas*) for *CLOCK*, two cetacean branches for *NPAS2*, the terminal branch of the Indo-pacific bottlenose dolphin (*Tursiops aduncus*) for *CRY1*, two cetacean branches for *PER2*, and five cetacean branches and the terminal branch of the Pacific walrus (*Odobenus rosmarus*) for *PER3* were subject to positive selection, respectively, although none remained after false discovery rate (FDR) correction (Fig 1, S4 Table). In contrast, one terrestrial branch for *PER3* (i.e., the terminal branch of the giant panda *Ailuropoda melanoleuca*) was found to be positively selected after FDR correction (Fig 1, and S4 Table). The adaptive Branch-Site Random Effects Likelihood (aBSREL) and the branch-site unrestricted statistical test for episodic diversification (BUSTED) methods available in Hyphy were further performed because these methods allow *d*_S_ to vary across sites and/or branches instead of constraining it to 1 as in PAML [37]. Evidence of positive selection within USWS-specific lineages was confirmed along the terminal branch of the beluga whale for *CLOCK*, the last common ancestor (LCA) of Cetacea for *NPAS2*, the terminal branch of the Indo-pacific bottlenose dolphin for *CRY1*, the terminal branch of the striped dolphin (*Stenella coeruleoalba*) for *PER2*, and the LCA of sperm whale (*Physeter catodon*) and dwarf sperm whale (*Kogia sima*), the LCA of Cetacea, the terminal branch of the sperm whale, and Pacific walrus for *PER3* (Fig 1, S5 and S6 Tables). In addition, BUSTED, a more efficient approach if only one or a few amino acid sites are under selection in the entire gene [38], also identified sign of positive selection at *NPAS2* gene in the West Indian manatee (*Trichechus manatus*) (*p* = 0.010; Fig 1, S6 Table).

### Associations between circadian clock gene evolution and sleep architecture

To explore the link between the evolutionary rate of circadian clock genes and sleep architecture, we performed phylogenetic generalized least squares (PGLS) regression analysis [39] between the log (root-to-tip ω) and the percentage of SWS out of total sleep time (TST), *i.e.*, the SWS/TST ratio that has been collected in previous study (S7 Table) [40], among mammals. The results showed that λ was 1 for the *CRY1* and *PER1* genes, suggesting a strong phylogenetic signal. This revealed that significant positive associations between molecular evolution and the SWS/TST ratio were found for *CRY1* (*R*^2^ = 0.378, *p* = 0.004) and *PER1* (*R*^2^ = 0.266, *p* = 0.014), although no significant associations were found for other six genes (S1 Fig and S8 Table).

### Convergent and lineage-specific amino acid substitutions

To assess convergent evolution in USWS species, we first reconstructed ancestral sequences for the internal nodes of the species tree to determine amino acid substitutions that were shared between the three different groups of marine mammals based on the JTT-f_genes_ model [41]. Although we found no convergent amino acid changes in the distantly related species, one unique substitution shared by cetaceans and the manatee was detected in *NPAS2* (T699P) and two unique substitutions shared by cetaceans and the walrus were detected in *PER2* (K127R) and *PER3* (I636V) (Fig 1). Of these substitutions, the PolyPhen-2 [42], SIFT [43], and PROVEAN [44] algorithms predicted that the convergent changes identified in *NPAS2* and *PER3* would affect the protein function (S9 Table).

Cetaceans have evolved a unique sleep pattern, dominated by USWS and an almost complete absence of REM sleep. The eight cetacean circadian genes were subsequently found to contain 47 unique amino acid substitutions, in total, that are absent in any other mammals (S2 Fig). Specifically, 15 cetacean-specific mutations were found in transcriptional activators, including three on *CLOCK*, four on *BMAL1*, eight on *NPAS2*, respectively. Similarly, for transcriptional repressors, *CRY1* and *CRY2* were found to have one cetacean-specific amino acid change, respectively, whereas the three *PERIOD* genes contained a total of 30 cetacean-specific replacements, including 11 on *PER1*, 11 on *PER2*, 8 on *PER3*. Furthermore, 61.70% (29/47) of these cetacean-specific changes were predicted to have functional significance by the PolyPhen-2, SIFT and PROVEAN algorithms (S10 Table).

To characterize the functional significance of these identified molecular changes, we mapped them onto the secondary structures and then compared the predicted three-dimensional structures of the corresponding wild-type (WT) and mutant proteins. The results showed that the all convergent substitutions and 78.72% (37/47) of cetacean-specific substitutions were located within or near key features of circadian clock proteins (S11 Table). Combined with results of functional prediction (S10 Table), two convergent substitutions (T699P in *NPAS2* and I636V in *PER3*) and 24 out of 37 cetacean-specific substitutions were considered to have more severe functional consequences. Of these unique substitutions, 17 substitutions were found among three *PERIOD* genes, and one, five and one were found on *BMAL1*, *NPAS2*, and *CYR2*, respectively. For example, eight potentially damaging mutations in the *PER3* gene all lie in or very close to the PAS/PAC domain, nuclear export signals, and CSNK1E- binding domain, which are responsible for circadian clock protein interactions, nucleo-cytoplasmic translocation, and phosphorylation modification, respectively, and thus may have a great effect on repressor activity. In contrast, for more conserved circadian activators, the identified convergent and cetacean-specific substitutions were located within intrinsically disordered regions mainly rather than conserved sequence structures (S11 Table). Three-dimensional structure prediction further indicated that the eight cetacean-specific substitutions of the *PER3* gene caused noticeable changes in the structures of WT and mutant proteins (template modeling score, i.e., TM score < 0.5, S12 Table). In addition, we unexpectedly found that WT and cetacean-specific mutant-type CLOCK proteins (M724V, S752P, and T779A) showed significant structural differences (S12 Table). Furthermore, the structures between CLOCK proteins from the bottlenose dolphin (*T. truncatus*), Pacific walrus, manatee, and their terrestrial relatives were significantly different (S13 Table), although the *CLOCK* gene showed a relatively high sequence conservation (95.4% identity) next only to *BMAL1* and *CRY1* (S3 Table). Molecular docking analysis subsequently revealed that cetacean-specific mutations led to an increase in the binding strength of CLOCK protein to its interactor BMAL1 (S14 Table), while cetacean-specific mutant BMAL1 (D3E, L456P, H461R, and M466T) also showed stronger interactions with CLOCK than WT BMAL1(S14 Table).

### *In vitro* functional comparison of cetacean-specific changes in CLOCK and BMAL1

**Fig 2:**
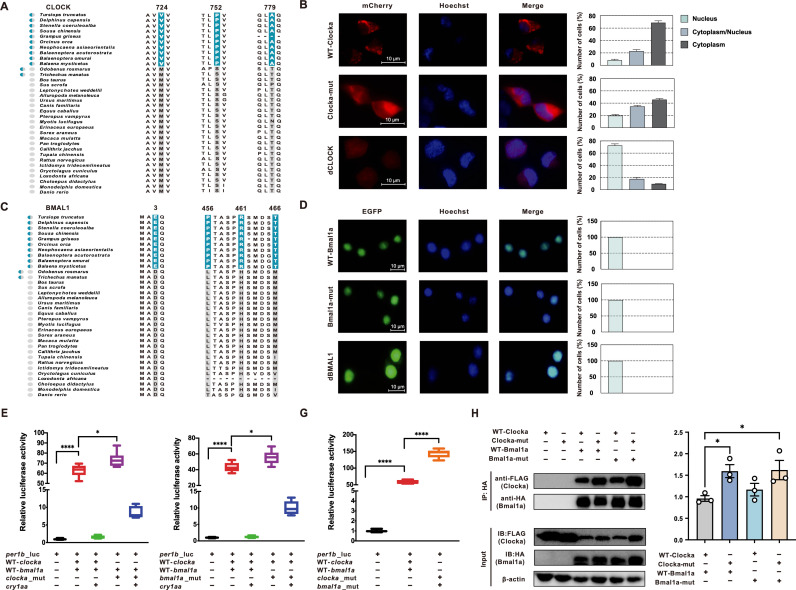
In vitro assays for the circadian transcriptional activators CLOCK and BMAL1. (A) Three cetacean-specific mutations in *CLOCK*. The amino acid substitutions in cetaceans are indicated in blue. (B) Differential subcellular localizations of WT-Clocka, Clocka-mut, and dCLOCK. (C) Four cetacean-specific mutations in *BMAL1*. The amino acid substitutions in cetaceans are indicated in blue. (D) The subcellular localization of WT-Bmal1a, Bmal1a-mut, and dBMAL1. Percentages of cells with fluorescence signals in nuclei only, cytoplasm only, or both were given on the right. (E to G) Clocka-mut (E), Bmal1a-mut (F), and the Clocka-mut/Bmal1a-mut complex (G) show increased transcriptional activation ability for the *per1b* promoter. (H) Lift, effect of cetacean-specific mutations on the interaction of Clocka and Bmal1a were evaluated by co-immunoprecipitation. Anti-HA agarose beads was used to precipitate HA-tagged WT-Bmal1a together or HA-tagged Bmal1a-mut with WT or mutant Flag-tagged Clocka. Immunoprecipitated proteins were further analyzed by western blotting with anti-HA and anti-FLAG antibody. Right, bar graph showing ImageJ densitometry of western blot from three independent experiments with single measurements, presented as mean arbitrary density units ± SEM relative to the WT-Bmal1a: WT-Clocka, while dots represent individual data points per experiment. *p < 0.05; ** *p* < 0.01; *** *p* < 0.001; **** *p* < 0.0001. Error bars represent ± SEM.

To evaluate the functional effects of the identified cetacean-specific replacements in *CLOCK* and *BMAL1* (Fig 2A and 2C), we first performed experiments on the subcellular localization and transcriptional activation *in vitro*. We used the cDNAs from zebrafish orthologs of mammalian *CLOCK* and *BMAL1* as the wild type (WT-*clocka* and WT-*bmal1a*). Then we obtained the mutant type, *clocka*-mut and *bmal1a*-mut by mutating the corresponding WT amino acid to cetacean-specific amino acid. Protein structure comparisons demonstrated that three cetacean-specific mutations had an obvious influence on the structure of zebrafish Clocka (TM score = 0.4994, S3 Fig), while Bmal1a-mut showed a conserved structure with WT-Bmal1a (S3 Fig). Molecular docking analysis further showed that the Clocka-mut and Bmal1a-mut complex had a higher number of hydrogen bonds as well as higher binding affinity compared to the WT-Clocka and WT-Bmal1a complex (S4 Fig). The localization results first showed that WT-*clocka* was predominantly localized in the cytoplasm, whereas bottlenose dolphin *CLOCK* (d*CLOCK*) was mostly located in the nucleus (Fig 2B). However, *clocka*-mut was found in both the cytoplasm and nucleus (Fig 2B). In contrast, *bmal1a*-mut and dolphin *BMAL1* (d*BMAL1*) had a similar cellular distribution to WT-*bmal1a* (Fig 2D). Furthermore, using the zebrafish *per1b* promoter-driven luciferase reporter, we found that both *clocka*-mut (*p* = 0.0032) and *bmal1a*-mut (*p* < 0.0001) possessed a stronger activation activity than WT groups (Fig 2E and F). The expression of zebrafish *cry1aa* significantly reduced the *clocka*-mut (*p* < 0.0001) and *bmal1a*-mut (*p* < 0.0001)-mediated activation of *per1b* promoter activity, indicating that the cetacean-specific mutations augmented the transcriptional activation function of *clocka* and *bmal1a* through a feedback loop mechanism. Importantly, the activation of *per1b* promoter activity in the co-expression mutant (expressing both *clocka*-mut and *bmal1a*-mut) was three times higher than that in the co-expression of WT groups (*p* < 0.0001, Fig 2G). In co-immunoprecipitation experiments, Clocka-mut showed a significantly increased interaction with WT-Bmal1a (*p* = 0.0184) and a greater tendency for binding to Bmal1a-mut (*p* = 0.1624) compared to WT-Clocka (Fig 2H). By contrast, Bmal1a-mut bound somewhat more strongly to WT-Clocka than WT-Bmal1a (*p* = 0.26844; Fig 2H). Furthermore, the interaction of Clocka-mut and Bmal1a-mut was also significantly higher than the interaction of WT-Clocka and WT-Bmal1a (*p* = 0.0471; Fig 2H), suggesting that cetacean-specific mutations indeed strengthened the interaction of Clocka and Bmal1a.

### *In vivo* functional evaluation of cetacean-specific changes in CLOCK and BMAL1

**Fig 3:**
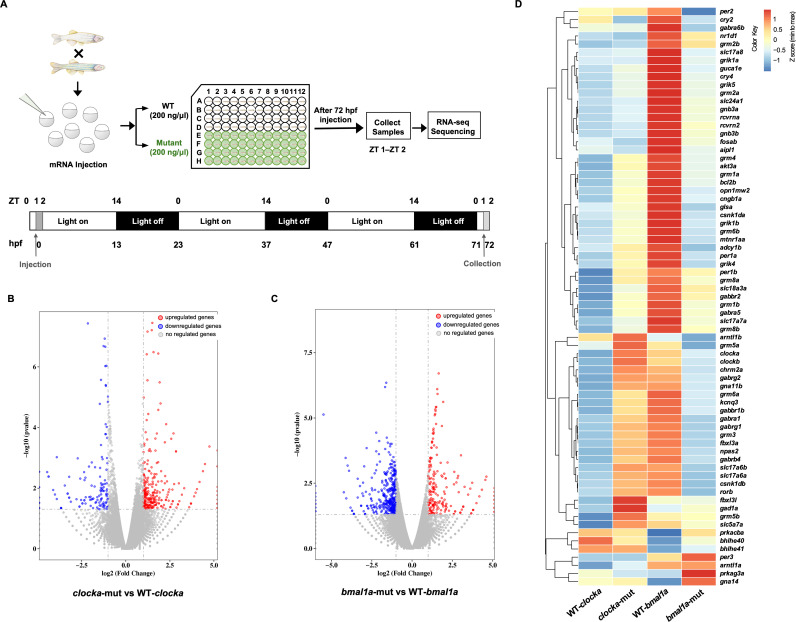
Transcriptome sequencing reveals changes in gene expression in zebrafish larvae overexpressing WT-*clocka*, *clocka*-mut, WT-*bmal1a*, and *bmal1a*-mut. (A) The schematic diagram shows the sample collection time of the mRNA-injected larvae. The transcriptome of the WT or mutant overexpression larvae and collected at 72 hpf, i.e., ZT 1–2 (10:00–11:00) on the third day after injection. (B) A volcano plot of differentially expressed genes between the *clocka*-mut group and the WT-*clocka* group. (C) A volcano plot of differentially expressed genes between the *bmal1a*-mut group and the WT-*bmal1a* group. (D) Heatmap of 71 genes related to sleep regulation in the WT-*clocka*, *clocka*-mut, WT-*bmal1a*, and *bmal1a*-mut groups.

To assess *in vivo* effects induced by cetacean mutations, we generated transgenic lines that transiently expressed either *clocka*-mut or WT-*clocka* in the stable transgenic zebrafish lines in the *clocka*^*−/−*^ background, and transgenic lines that transiently expressed either *bmal1a*-mut or WT-*bmal1a* in the stable *bmal1a*^*−/−*^ zebrafish. We first found that larvae of these transgenic zebrafish were healthy and had normal morphological architecture (S5 and S6 Figs), and all larvae were then collected for gene expression analysis at 72 hours postfertilization (hpf), which corresponded to a time point between zeitgeber time (ZT) 1 and ZT 2 when considering the light-dark cycle (Fig 3A). The subsequent transcriptome sequencing identified 407 differentially expressed genes (DEGs; 265 upregulated and 142 downregulated genes) (log_2_ fold change > 1, *p* < 0.05) in the *clocka*-mut group when compared with the WT*-clocka* group (Fig 3B). Furthermore, 590 DEGs (199 upregulated and 391 downregulated genes), were found in the *bmal1a-mut* when compared with the WT-*bmal1a* group (Fig 3C).

**Fig 4:**
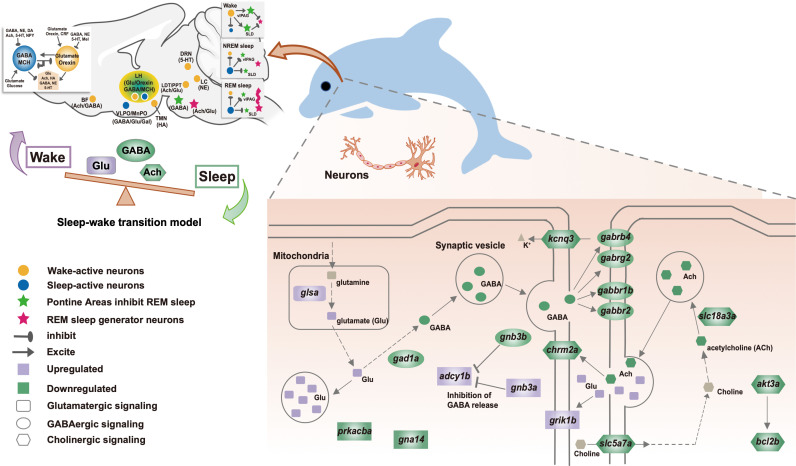
Gene expression alterations in the glutamatergic, GABAergic, and cholinergic pathways of cetaceans may help to inhibit sleep and promote wakefulness. Purple indicates genes with increased expression, and dark green genes with reduced expression in transcriptome analysis. A network map of neurons regulating waking, NREM and REM sleep is summarized based on the previous studies [134–136]. The abbreviations in the bracket indicate their corresponding neuronal types. Ach, acetylcholine; BF, basal forebrain; CRF, corticotrophin-releasing factor; DA, dopamine; DR, dorsal raphe; glu, glutamate; HA, histamine; LC, locus coeruleus; LH, lateral hypothalamus; MnPO, median preoptic area; NE, norepinephrine; Mel, melatonin; NPY, neuropeptide Y; SLD, sublateral dorsal nucleus; TMN, tuberomammillary nucleus; vlPAG, ventral lateral periaqueductal gray; 5-HT, serotonin.

We further performed Gene Ontology (GO) [45] and Kyoto Encyclopedia of Genes and Genomes (KEGG) [46] enrichment analysis to determine the function of these DEGs. The results showed that the 407 DEGs identified in the *clocka*-mut group compared with the WT-*clocka* group were enriched in genes associated with circadian rhythm, signal transduction, and synaptic functions (Figs 3D, S7 and S8). Among them, eight DEGs (i.e., *rorb*, *mtnr1aa*, *adcy1b*, *gnb3a*, *fosab*, *prkag3a*, *prkacba*, and *gna14*) were annotated as rhythm-related terms, including “circadian entrainment” and “circadian rhythm.” Eight other DEGs (*crx*, *opn1mw2*, *cngb1a*, *aipl1*, *guca1e*, *rcvrna*, *rcvrn2*, and *slc24a1*) were found to be directly related to the light sensitivity and light signal transduction, which were all upregulated with a fold change of 1.37–3.20. Furthermore, 14 DEGs were involved in excitatory and inhibitory neurotransmitter transmission, which may be broadly linked to sleep regulation. For example, four upregulated genes (*glsa*, *grik1b*, *gnb3a*, and *adcy1b*) and two downregulated genes (*gna14* and *prkacba*) were significantly enriched within “glutamatergic synapse.” In contrast, the GO and KEGG analyses of 590 DEGs for *bmal1a*-mut versus WT-*bmal1a* revealed marked alterations of genes involved in three main functional categories of DNA binding, neurotransmitter transport, and signal transduction (Figs 3D, S9 and S10). Notably, genes associated with “GABAergic synapse” and “cholinergic synapse” were significantly downregulated. Specifically, in the bmal1a-mut group, six DEGs related to the GABA neurotransmitter system, including *gad1a*, *gabrb4*, *gabbr1b*, *gabrg2*, *gabbr2*, and *gnb3b*, were downregulated by between 0.28-fold and 0.47-fold, and six DEGs (*chrm2a*, *kcnq3*, *slc18a3a*, *slc5a7a*, *bcl2b*, and *akt3a*) assigned to the cholinergic system were downregulated by between 0.33-fold and 0.44-fold. To investigate whether DEGs are subjected to circadian regulation, we further compared them with genes that were identified as circadian in larval zebrafish [47], human and mouse [48] circadian transcriptomic data sets from CircaKB database (https://cdsic.njau.edu.cn/CircaKB). The results first showed that 83 DEGs identified in the *clocka*-mut versus WT-*clocka* and 102 DEGs identified in the *bmal1a*-mut versus WT-*bmal1a*, were characterized as having a circadian expression profile (S11 Fig). KEGG enrichment analysis showed that 83 DEGs with a circadian expression profile were also enriched within “phototransduction,” “circadian rhythm,” and “glutamatergic synapse” (S12 Fig), while 102 circadian oscillating DEGs also showed an association with “circadian entrainment” and “cholinergic synapse,” except “GABAergic synapse” (S13 Fig). These results suggested that cetacean-specific changes in *CLOCK* and *BMAL1* genes may have an effect on circadian regulation of sleep (Fig 4).

**Fig 5:**
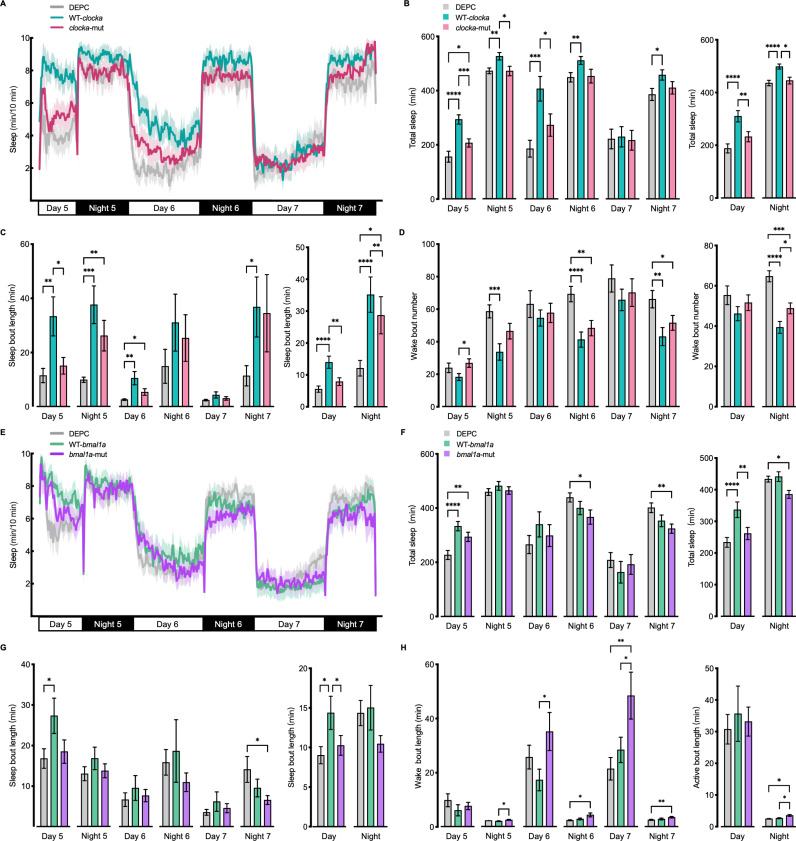
Cetacean-specific mutations in CLOCK and BMAL1 alter sleep/wake-related behaviors. (A-D) Sleep activity traces (A), total sleep time (B), sleep bout length (C), and wake bout number (D) were characterized in the DEPC-injected control (n = 32), WT-*clocka* overexpression (n = 32) and *clocka*-mut overexpression (n = 40) zebrafish larvae. (E-H) Sleep activity traces (E), total sleep time (F), sleep bout length (G), and wake bout number (H) were characterized in the DEPC-injected control (n = 62), WT-*bmal1* overexpression (n = 39) and *bmal1a*-mut overexpression (n = 46) zebrafish larvae. Behavioral recording was initiated on 5 dpf. n indicates the number of animals. *p < 0.05; ** *p* < 0.01; *** *p* < 0.001; **** *p* < 0.0001. Error bars represent ± SEM.

To determine whether cetacean-specific mutations affect sleep/wake behaviors, we then analyzed sleep in zebrafish overexpressing *clocka*-mut, WT-*clocka*, *bmal1a*-mut, and WT-*bmal1a* with a high-throughput behavioral assay, respectively. We observed that the overexpression of WT-*clocka* in wild-type zebrafish significantly increased total sleep time during both the day (*p* < 0.0001) and night (*p* < 0.0001) compared with diethylpyrocarbonate-treated (DEPC) water-injected control group (Fig 5, A and B). In contrast, sleep time in *clocka*-mut overexpression larvae was slightly longer than in control group (day, *p* = 0.4984; night, *p* = 0.1413) but obviously shorter than in WT-*clocka*-overexpressing group (day, *p* = 0.0041; night, *p* = 0.0151) (Fig 5, A and B). For example, the WT-*clocka* overexpression larvae had an almost 65% increase in the average amount of daytime sleep compared with the control group, whereas the *clocka*-mut overexpression larvae had only a 23% increase. Furthermore, analysis of sleep architecture further showed that the *clocka*-mut overexpression larvae had a significantly reduced duration of sleep bouts during the day (*p* = 0.0084) and night (*p* = 0.0054) (Fig 5C) and a significantly increased number of wake bouts at night (*p* = 0.0241) (Fig 5D) compared with the WT-*clocka* overexpression group. In addition, the *bmal1a*-mut overexpression larvae also showed a significant decrease in sleep during the day (*p* = 0.0099) and a trend to sleep less at night compared to the WT- *bmal1a* overexpression group (Fig 5, E and F), which was due to a significantly shorter duration of sleep bouts during the day (*p* = 0.0489) (Fig 5G) and a significantly longer duration of wake bouts at night (*p* = 0.0400) (Fig 5H).

## Discussion

Air-breathing mammals that sleep in water face many challenges, such as vulnerability to predation, thermoregulatory imbalance, and most of all, preventing the inhalation of water into the lungs while breathing. Accordingly, marine mammals, including cetaceans, sirenians, and pinnipeds (except true seals) have evolved USWS, an unusual sleep behavior in brain hemispheres undergo alternate periods of sleep and wakefulness, to resolve the contradictions between sleep and the various life-sustaining demands [7]. Notably, cetaceans are found to have only USWS, with no signs of REM sleep. However, the underlying molecular basis of USWS is currently unclear. Here, we reported a comparative analysis and functional experiments on canonical circadian clock genes to explore the molecular mechanism of the unique USWS observed in marine mammals. Significant signals indicating positive selection were mostly found in cetacean lineages only adapted to unihemispheric sleep, indicating that cetacean circadian clock genes may have undergone adaptive changes to satisfy the requirements for underwater sleep. *In vitro* and *in vivo* assays using the model species zebrafish further supported that the identified cetacean-specific mutations enhanced the transcriptional activation ability of *clocka* and *bmal1a*, as well as altering the expression of the sleep-related signaling pathways, and correspondingly reduced ability to maintain sleep, which might be beneficial for their special sleep pattern.

### Signatures of the adaptive changes in canonical circadian clock genes contributing to USWS

The timing and duration of sleep are greatly influenced by the circadian system, in which the canonical circadian clock genes play a crucial role [11,49]. Indeed, mutant phenotypes from animal studies suggest that CLOCK and BMAL1 are not only important for the maintenance of circadian rhythmicity, but also for the adjustment of the sleep-wake cycle [50]. Furthermore, the genetic variations in the *CRY* and *PER* genes have been closely linked to sleep disorders with disturbed sleep timing [51]. Lineage-specific selection analyses found extensive positive selection at *CLOCK*, *NPAS2*, *CRY1*, *PER2*, and *PER3* genes along cetacean lineages. Furthermore, cetacean-specific amino acid substitutions were also observed in these positively selected genes, 83.87% (26/31) were located within or near important functional structures, which might involve adaptive functional changes. For example, cetacean *PER3* were found to contain eight unique mutations, all of which were predicted to impact protein’s function and were situated in key domains determining the inhibitory activity of PER3 protein, including one in PAS-A domain, one in PAC domain, three in the nuclear export sequences, and three in CSNK1E-binding domain, respectively [52–54]. PER3 was reported to have a role in sleep–wake timing, as *Per3*-knockout mice exhibited reduced total sleep duration in the middle of the dark phase [55]. Therefore, significant positive selection and putative functional modifications in cetacean *PER3* may help to promote short and fragmented sleep activity to keep one side of the brain awake during USWS. By contrast, for circadian transcriptional activators, most specific mutations were detected within the intrinsically disordered regions, which have been known to serve critical roles in the regulation of circadian period and amplitude by modulating the interaction between the positive and negative constituents [56, 57]. Modification or variations in the disordered regions of the circadian repressors were shown to alters the period as well as the amplitude of the rhythms [58, 59]. For instance, CRY1 contains a significantly disordered C-terminal tail and genetic truncation of this tail led to an established case of human delayed phase sleep disorder [60]. In addition, CRY1 also competes with CLOCK for binding on the disordered transactivation domain on the C-terminal of BMAL1 to build a functional switch between activating and inactivating CLOCK/BMAL transcriptional activity [61]. However, molecular changes in disordered regions of circadian activators that have a direct impact on activation activity have been rarely reported. The unique mutations identified in cetacean *CLOCK* and *BMAL1* genes were predicted to facilitate interaction with each other, which could cause an obvious increase in transcriptional activity of the CLOCK/BMAL1 complex. This supports the potential importance of CLOCK/BMAL1-mediated transcription in cetacean sleep adaptation and possibly provides further novel evidence for the evolutionary adjustment of functions of intrinsic disorder in the circadian positive regulators.

Another critical evidence comes from the observation that cetacean-specific mutations caused functional changes in *CLOCK* and *BMAL1 in vitro* and *in vivo*. It is well-known that CLOCK protein is distributed in the nucleus and cytoplasm, and that nuclear CLOCK exerts transactivation activity [62, 63]. The subcellular localization analysis uncovered that WT-*clocka* was predominantly localized in the cytoplasm and dolphin *CLOCK* was predominantly located in the nucleus. Interestingly, for *clocka*-mut, nuclear localization was dominant over cytoplasmic localization, suggesting that the three cetacean-specific mutations have a significant effect on the cellular localization of CLOCK protein. Significantly, we also found that *clocka*-mut had significantly greater ability to activate *per1b* transcription than WT-*clocka*, indicating that cetacean *CLOCK* has evolved a strong transcriptional activation ability to regulate the expression of its downstream genes. Similarly, the four cetacean-specific mutations in *BMAL1* were also found to increase the transcriptional activation compared with WT-*bmal1a*. As expected, cetacean-specific mutant Clocka and Bmal1a also showed enhanced binding affinity than WT. The circadian clock is involved in stimulating wakefulness during the daytime by counteracting the increasing need for sleep during the waking period [64]. Previous studies showed that mice carrying *Clock* mutation exhibit reduced transcriptional activity of the CLOCK protein and behavioral abnormalities, including hyperactivity, increased arousal, and irregular sleep–wake rhythm [65, 66]. Moreover, the *Bmal1*-knockout mice, which have disrupted activation activity, became immediately arrhythmic in constant darkness and displayed a 1.5-hour increase in total sleep [67]. Therefore, it can be seen that the cetacean-specific mutations of *CLOCK* and *BMAL1* enhanced their transcriptional activation function, which may be conducive to the continuous stimulation of two alternate sides of the brain to maintain prolonged wakefulness during USWS. Our behavioral observations provided support for this that both the *clocka*-mut overexpression zebrafish and *bmal1a*-mut overexpression zebrafish exhibited significantly reduced sleep time and shorter duration of sleep bouts than the wild-type overexpression zebrafish, indicating that functional changes in cetacean *CLOCK* and *BMAL1* are likely to play a positive role in depressing whole-brain sleep need.

It has also been revealed the importance of paralogs of *CLOCK* and *BMAL1*, i.e., *NPAS2* and *BMAL2*, in tuning circadian timing. As a functional substitute for CLOCK [68], NPAS2 can dimerize with other circadian factors BMAL1 and BMAL2 to form transcriptionally active complexes [17] and compensate for the loss of CLOCK in peripheral cells as well as in the suprachiasmatic nucleus (SCN), the master circadian pacemaker [69]. We found that *NPAS2* was identified to be under positive selection in the common ancestor of all cetaceans and contains eight cetacean-specific amino acid substitutions. Six of these substitutions were predicted to cause functional alterations, with two substitutions (N131R and E246K) being located in the heme-containing PAS-A and PAS-B domain, respectively (S10 and S11 Tables). The two PAS domains bind to heme directly as a prosthetic group, thus controlling the heme status of NPAS2 and related DNA-binding activity [70]. The mutation (H119A or H171A) in the PAS-A domain of NPAS2 resulted in the loss of DNA binding to the E-box elements and a defect in the heterodimer formation with BMAL1 [71]. Similar to CLOCK, these substitutions in cetacean *NPAS2* may be beneficial in enhancing the DNA-binding and transcriptional activity. Furthermore, like BMAL1, BMAL2 can form heterodimers with CLOCK or NPAS2 to activate the E-box-dependent transcription [72]. It was shown that *BMAL2* is regulated by *BMAL1*, and constitutive expression of *BMAL2* can rescue the arrhythmic circadian phenotypes of *BMAL1*-knockout mice [73]. Our previous work revealed that two unique amino acid substitutions in cetacean *BMAL2* facilitated the expression of *PER2* and the related wakefulness-promoting effect by specifically increasing the response to an E-box-like enhancer [74]. We hypothesize that functional innovation in cetacean circadian transcriptional activators may contribute to sustain wakefulness in one hemisphere during USWS.

In addition to circadian factors, the regulation of sleep and wake requires the coordination of various neurotransmitters [26]. For example, the role of gamma-amino butyric acid (GABA) in sleep induction and maintenance is well accepted, while glutamate, as the major precursor of GABA, is an essential wake-promoting neurotransmitter [75, 76]. Among others, dopamine, noradrenaline, histamine, and serotonin act together for the generation and consolidation of wakefulness[77]. Conversely, acetylcholine, melatonin and several peptide factors such as adenosine are involved in initiating and promoting sleep [78]. Here, six DEGs related to glutamate, a key neurotransmitter for arousal maintenance [79], were identified in the stable *clocka*^*−/−*^ zebrafish line that transiently expressed *clocka*-mut. In particular, the *glsa* gene encoding glutaminase, which catalyzes the production of glutamate, and the *grik1b* gene encoding glutamate receptor 5, which is implicated in excitatory synaptic transmission, were independently upregulated 2.39-fold (*p* = 0.0051) and 3.04-fold (*p* = 0.0027) in the *clocka-*mut zebrafish compared with the control. These changes may suggest an increased accumulation of glutamate and following enhanced excitatory activation. By contrast, genes associated with GABA, a sleep-promoting neurotransmitter, were significant downregulated in the *bmal1a*-mut zebrafish, including *gad1a*, which encodes glutamate decarboxylase that converts glutamate into GABA, and four GABA receptor subunit encoding genes (*gabrb4*, *gabbr1b*, *gabrg2* and *gabbr2*). Previous studies revealed that mice deficient in individual GABA receptor subunits have abnormal sleep phenotypes that are characterized by a lack of electrocortical signatures of SWS and decreased theta activity in REM sleep [80, 81]. These findings lead us to suggest that the cetacean-specific mutations found in CLOCK and BMAL1 may contribute to suppressing the global sleep-promoting effects, sustaining wakefulness by elevating glutamate levels, and reducing conversion to GABA. Furthermore, eight genes related to light perception (e.g., *crx*, *opn1mw2*, *cngb1a*, *aipl1*, *guca1e*, *rcvrna*, *rcvrn2*, and *slc24a1*) were upregulated in the *clocka-*mut zebrafish. These genes also affect the timing of sleep, for example, mutations in human *CRX* and *AIPL1* were shown to cause inherited retinopathy and the associated symptoms of severe sleep abnormalities or insomnia [82]. In cetaceans, the contralateral eye to the waking hemisphere was almost always open during an episode of USWS [9], indicating that the light input during a period of USWS plays a role in maintaining the wakefulness of the contralateral hemisphere [6]. Altered light perception could affect the frequency of alternating USWS episodes, as revealed in a mathematical model of USWS [83].

### Positive role of canonical circadian clock genes in the inhibition of REM sleep

As fully aquatic mammals, cetaceans have a complete elimination or negligible amount of REM sleep to maintain temperature and avoid drowning [7], because muscle tone becomes absent and the temperature regulating machinery is suspended during REM sleep [40]. Our study provided two pieces of evidence supporting the contributions of canonical circadian clock genes to the loss of REM sleep in cetaceans. First, we found that cholinergic-related genes were significantly downregulated in the *bmal1a*-mut zebrafish compared to the wild-type zebrafish. The six downregulated cholinergic-related genes included the receptor-encoding gene *chrm2a* and the transport-related genes *slc18a3a* and *slc5a7a*. The cholinergic system is an important and conserved modulator of REM sleep initiation [84]. In zebrafish, the cholinergic system shows similar receptor components, genetic homology, and cellular distribution to mammals [85]. For example, telencephalic and tegmental cholinergic clusters in zebrafish were found to correspond to the mammalian cholinergic basal forebrain and mesopontine system [86], which engage in regulating the REM sleep switch [87, 88]. Furthermore, a recent study revealed that zebrafish have a sleep state named propagating wave sleep (PWS), which shares commonalities with mammalian REM sleep, including a total loss of muscle tone, increased variability in heart rate, etc. In particular, a cholinergic agonist, carbachol, was shown to promote PWS while muscarinic acetylcholine receptor antagonists inhibit PWS, similar to the effect of the cholinergic system on REM sleep in mice. Moreover, knocking out cholinergic receptor muscarinic genes, i.e., *Chrm1*, *Chrm2*, resulted in reduced or fragmented REM sleep in mice [89, 90]. Moreover, SLC18A3 is involved in releasing acetylcholine into the extracellular space [91], whereas SLC5A7 is involved in mediating choline uptake to control acetylcholine synthesis [92]. The diminished expression of these genes possibly implies the attenuation of the cholinergic system in cetaceans, which is supposed to blocking the maintenance of REM sleep. Second, our regression analyses showed that *CRY1* and *PER1* were significantly related to the evolution of the SWS/TST ratio in mammals, suggesting the key roles of these two genes in sleep regulation. In particular, one and seven cetacean-specific mutations were detected in *CRY1* and *PER1*, respectively. Previous studies revealed that both the *CRY1* and *PER1* genes were implicated in the regulation of REM sleep. Mice lacking both *Cry1* and *Cry2* exhibited altered sleep structure and there was no rebound in REM sleep after sleep deprivation compared with the wild-type [93]. Furthermore, one cetacean-specific replacement (Q532P) was found at the C-terminal region, encoded by exon 11, which has been demonstrated to be essential for regulating the affinity of CRY1 for the CLOCK:BMAL1 complex [94]. The deletion of exon 11 resulted in the enhanced repression activity of CRY1 and a lengthened circadian period in mice [60]. Similarly, two cetacean-specific replacements (A652T and D711G) were identified within the phosphorylated region of PER1, which mediates the nuclear localization, ubiquitination, and subsequent degradation of the PER1 protein [95]. The increase in PER1 level was coordinated with the need for sleep [27]. In mice with *Clock* missense mutation, the level of *Per1* expression was reduced and REM sleep rebound increased [50]. Potentially, the changes in the transcriptional repression function and expression of CRY1 and PER1 might contribute to preventing REM sleep in cetaceans; indeed, this should be investigated further. Overall, these results may provide evidence that the functional adaptations that have occurred in cetacean circadian clock genes are not only advantageous for adapting unihemispheric sleep but also for inhibiting REM sleep.

In addition, it could be speculated that maintaining slow accumulation of sleep need is essential for marine mammals to avoid damage by extended wake periods and to suppress bursts of REM sleep. We found three specific amino acid substitutions in *NPAS2* (T699P), *PER2* (K127R), and *PER3* (I636V) among the three different marine groups with USWS. Furthermore, *NPAS2* was inferred to have evolved under positive selection in manatee and the ancestor of cetaceans, and *PER3* was found to be positively selected in walrus and the ancestor of cetaceans, suggesting evidence of molecular convergence in marine mammals. It was shown that NPAS2 is functionally implicated in the homeostatic regulation of sleep and contributes to coupling waking to cortical *PER2* expression [96]. Several studies indicated that increased *PER2* expression in the cerebral cortex showed a significant association with elevated sleep need [93]. The sustained, high levels of PER2 expression may exert a negative influence on recovery sleep and the EEG oscillations during NREMS [97]. Mice homozygous for the *Npas2* deletion lacked rhythmic *PER2* expression in the cortex and exhibited less sleep during the late active period, a slower rate of sleep need accumulation, and a reduced compensatory rebound in NREM sleep after sleep deprivation [96,98]. Convergent signals detected in *NPAS2* and its target *PER2* may have some effect on this special interactions and wake-dependent expression increase, which coincides with our observation of specifically enhanced activation of cetacean-specific mutant *BMAL2* to *PER2* expression [74]. In particular, convergent K127R mutations was located at nuclear export signal of PER2 protein that is responsible for nucleocytoplasmic shuttling. The subcellular distribution of PER2 has been documented to affect its own degradation and thus played a role in synchronizing circadian rhythms of suprachiasmatic neurons and sleep homeostatic regulation [99,100]. These findings further reflected the significance of improved PER2 levels in relieving the growing sleep pressure in marine mammals. Additionally, the convergent site 636 of the *PER3* gene was found in the CSNK1E binding domain required for PER3 phosphorylation and subsequent degradation [101]. Mutations that impact the interaction of *CSNK1E* to PER proteins have been demonstrated to change the circadian period of behavior in mice and human [102,103]. PER3 has been thought to contribute to individual differences in homeostatic responses and timing of sleep by affecting circadian rhythmicity [55,104], whose polymorphisms are strongly associated with diurnal preference and delayed sleep phase syndrome [105]. Correspondingly, the convergent changes in the *NPAS2, PER2*, and *PER3* sequences may drive a similar and slow homeostatic accumulation in marine mammals to achieve advantages in wakefulness during USWS.

## Conclusion

Our study investigated the molecular genetic basis of sleep patterns in marine mammals. Strong signatures of positive selection were detected for five circadian genes (*CLOCK*, *NPAS2*, *CRY1*, *PER2*, and *PER3*) in cetaceans, which matched well with the evolution of the unique cetacean USWS. Functional assays uncovered that dolphin *CLOCK* had predominantly nuclear localization, whereas zebrafish *clocka* was mostly cytoplasmic, and that cetacean-specific mutations could also affect the subcellular location of *clocka*. Moreover, both cetacean-specific mutant *clocka* and *bmal1a* exhibited stronger transcriptional activation activity than zebrafish wild-type. These findings support the occurrence of adaptive changes in cetacean circadian clock genes. *In vivo* assays further indicated that the cetacean-specific mutant *clocka* and *bmal1a* may have an inhibitory effect on sleep demand by affecting glutamate and GABA signaling, and the downregulation of the cholinergic pathway is assumed to be related to the loss of REM sleep in cetaceans. Furthermore, convergent amino acid substitutions were found among the *NPAS2*, *PER2*, and *PER3* genes of cetaceans, the manatee, and the walrus, which was in line with the phenotypic convergence of sleep patterns in these three distinct marine groups. In summary, our findings suggest that most marine mammals have evolved an effective and convergent mechanism for sleep to better adapt and thrive in the complex habitat of the aquatic environment.

### Limitations

Although we revealed the adaptative features of canonical circadian clock genes and the potential role in the evolution of USWS, there remains a few limitations of our approach and *in vivo* models. Present study used zebrafish as model species and injection-based gene overexpression to determine the effect of cetacean-specific mutations on sleep reliably, reproducibly, and cost-effectively [106]. However, zebrafish exhibit a more complex circadian feedback loop than mammals due to teleost-specific whole genome duplication, which contains two *bmal1* genes (*bmal1a* and *1b*) and two *clock* genes (*clock1a* and *1b*) [107]. This may complicate our assessment, as it is difficult to eliminate the influence of their paralogs though the predominant roles of *bmal1a* and *clock1a* in circadian regulation have been supported by several studies [108–110]. Additionally, dose flexibility of microinjection may raise concerns that detected differences comes from the different expression levels of the transgenes rather than solely from the presence of the mutations. Similarly, a single sampling time-point limits our observations on dynamic changes of circadian regulatory networks. Besides, it is also hard to determine whether the circadian phase of clock genes expression changed at the molecular level based on a single sampling time-point, which may have an important effects on the expression patterns of rhythm related genes and pathways [111], although no phase advance or delay in sleep rhythm between WT and mutant groups. More research using gene-edited mice or stable transgenic zebrafish is needed in future to refine and better understand the association between functional modifications in canonical circadian clock genes and the generation of USWS.

## Materials and methods

### Ethics statement

This study was approved by the Animal Ethical and Welfare Committee of Nanjing Normal University (IACUC-20190101).

### Sequence acquisition and alignments

Orthologous sequences of 8 circadian clock genes from 31 mammals, of which 11 were marine mammals, bottlenose dolphin, killer whale (*Orcinus orca*), Yangtze River dolphin (*Lipotes vexillifer*), sperm whale, Yangtze finless porpoise (*Neophocaena asiaeorientalis*), minke whale (*Balaenoptera acutorostrata*), bowhead whale (*Balaena mysticetus*), Pacific walrus, Weddell seal (*Leptonychotes weddellii*), polar bear (*Ursus maritimus*), and West Indian manatee, and 20 terrestrial mammals, were downloaded from NCBI (http://www.ncbi.nlm.nih.gov), Ensembl (http://www.ensembl.org), and the Bowhead Whale Genome Resource (http://www.bowhead-whale.org/). For each gene, the longest transcript was kept in our analysis. The accession numbers for all circadian genes are listed in S2 Table. Furthermore, we amplified and sequenced the eight circadian clock genes from other nine cetaceans (eight toothed whales and one baleen whale): striped dolphin (*Stenella coeruleoalba*), saddleback dolphin (*Delphinus delphis*), Indo-pacific bottlenose dolphin, Indo-pacific humpbacked dolphin (*Sousa chinensis*), Risso’s dolphin (*Grampus griseus*), beluga whale, Blainville’s beaked whale (*Mesoplodon densirostris*), dwarf sperm whale, and Omura’s baleen whale (*B. omurai*). The samples of these nine cetaceans were collected from dead individuals in the wild and sampling was conducted systematically in accordance with all the ethical guidelines and legal requirements in China. Detailed information about cetacean samples is described in S15 Table. Genomic DNA extraction, polymerase chain reaction (PCR) amplification and sequencing were performed as previously described [112]. Additionally, the low quality or unannotated CDS in the database was further verified using BLASTn searches. Overall, circadian clock genes among 40 species from representative clades of mammals were analyzed in the present study. We then used two alignment methods, i.e., CLUSTAL and MUSCLE, as implemented in MEGA 11.0 [113] to align the nucleotide sequences of each gene and verified by visual inspection.

### Molecular evolution analysis

The nonsynonymous (*d*_N_) to synonymous (*d*_S_) substitution ratios (ω = *d*_N_/ *d*_S_) were used to evaluate selection pressure, with ω = 1, < 1, and > 1 indicating neutral evolution, purifying selection, and positive selection, respectively. To investigate the possible occurrence of positive selection in mammalian circadian clock genes, ω values were estimated using the codon-based maximum-likelihood (ML) models implemented in the CODEML program of the PAML ver. 4.10.6 package [35]. The well-supported mammalian phylogeny obtained from multiple references was used as the input tree in all analyses (Fig 1). Phylogenetic trees were also estimated from the nucleotide and amino acid sequences of eight circadian clock genes by ML and Bayesian inference (BI) approaches (S14 and S15 Figs). The gene trees were similar to the currently well-established phylogeny, with some minor differences only within Perissodactyla, Chiroptera and Eulipotyphla. Because the minor differences in the phylogeny did not result in any significant difference in the identification of positively selected sites [114], the results of selection detection based on the gene trees were similar to those obtained based on the well- accepted species tree, only the latter results was reported here.

To evaluate whether positive selection was restricted to USWS-specific lineages, we used branch-site model implemented in CODEML [115]. Branch-site models require the foreground branches (lineages tested to be under positive selection) and background branches (rest of the lineages) to be defined a priori. Each lineage across the mammalian phylogeny was used as the foreground branch, respectively, whereas the remaining branches were treated as background branches for each gene. The likelihood ratio test (LRT) with a χ^2^ distribution was applied to evaluate if models were statistically different from the null model at a threshold of *p* < 0.05. BEB analysis was used to identify positively selected sites with posterior probabilities of ≥ 0.80 [35]. The FDR correction for multiple tests was applied to the LRT *p* values for branch-site model analysis [34]. In addition, two independent tests in HyPhy were futher used to detect signals of positive selection: the aBSREL method which assumes that foreground branches may be subject to episodic positive selection at a proportion of sites [116], and the BUSTED method to test for positive selection of a gene at any site on foreground branches (at least one site on at least one branch) [38].

### Correlation analysis

PGLS regression methods were utilized to detect different variable relationships while accounting for phylogenetic relationships. PGLS incorporates phylogenetic information into generalized linear models to provide a powerful method for analyzing continuous data, and this has been utilized to test for correlations among the evolutionary models and the relationships among life-history traits. In the PGLS regression analysis, the value of the lambda (λ) was estimated using ML to optimally adjust the degree of phylogenetic correlation among data sets. The λ values can range from 0 to 1; λ = 0 indicates no phylogenetic signal whereas λ = l indicates a strong phylogenetic signal [117]. We used PGLS methods to explore the relationship between the evolutionary rate (*i.e.*, ω) of circadian clock genes and the SWS/TST ratio. The evolutionary rate root-to-tip ω was estimated using the two-ratio model implemented in the CODEML program of PAML 4.7 [35]. The data for mammalian SWS/TST ratios were obtained from the published data (S7 Table) [40]. PGLS regression analysis was performed using the Caper package in R (version 3.1.2) [39].

### Identification of convergent substitutions and shared specific amino acids

We first reconstructed the ancestral amino acids sequences of eight circadian clock genes using the CODEML program in PAML ver. 4.10.6 [35], and used the Zhang and Kumar’s method to detect convergent amino acid substitutions in independent pairs of USWS lineages [118]. Accounting for the noise resulting from the random amino acid substitutions of convergence, the JTT-f_genes_ model of amino acid substitution was used to estimate the expected number of molecular convergences in each protein alignment [41]. Finally, a Poisson test was performed to verify whether the observed number of convergent sites in each gene was significantly more than the number expected by random substitution. Next, FasParser2 [119] was used to detect unique amino acid substitutions based on sequence alignments, as convergent phenotypic characteristics can also arise from unique substitutions that have independently evolved in different species.

Furthermore, a substitution was defined as cetacean-specific for each amino acid site within the circadian clock protein if the amino acid in all cetaceans differed from that of all other species. Shared specific substitutions were identified by strict identity. To identify the shared specific amino acid mutations in cetaceans, in-house Perl scripts were employed to examine each column of the trimmed amino acid alignments. Three free online protein structure prediction programs, PolyPhen-2 (http://genetics.bwh.harvard.edu/pph2/) [42], SIFT (https://sift.bii.a-star.edu.sg/index.html) [43] and PROVEAN (http://provean.jcvi.org/index.php) [44], were then used to predict the impact of these conserved mutations using the default cutoff values. Human protein sequences were used as queries for prediction programs. The predictions “possibly damaging” or “probably damaging” by PolyPhen-2, “damaging” by SIFT, and “deleterious” by PROVEAN were all regarded as function-altering substitutions.

Three-dimensional protein structures were calculated with alphafold v2.3.1 using the “monomer_ptm” model [120]. The topological similarity of protein structures were assessed with template modeling score (TM-Score) [121]. Rigid protein–protein docking was performed using GRAMM-X (http://gramm.compbio.ku.edu/) [122]. PDBePISA (https://www.ebi.ac.uk/pdbe/pisa/) and PyMOL (Version 4.6.0) were used to investigate protein-protein interactions and further visual analysis.

### *In vitro* functional assays

We conducted cell-based functional assays to examine whether cetacean circadian clock genes resulted in functional differences and whether the identified cetacean specific site changes would result in functional innovation. It has been demonstrated that there are zebrafish contained two *clock* gene (*clocka*, and *clockb*) and two *bmal1* gene (*bmal1a*, and *bmal1b*) due to the teleost specific genome-wide duplication [123]. Of them, *clocka and bmal1a* have higher homology with mammalian orthologs (*clocka*: 72.01%, *bmal1a*: 86.75%) compared to their paralogs (*clockb*: 63.94%, *bmal1b*: 85.99%). Furthermore, growing evidence indicates that *clocka* and *bmal1a* may play a more dominant role in the zebrafish circadian circuit than their paralogs. First, the yeast two-hybrid system showed that Bmal1a rather than Bmal1b can heterodimerizes with Clocka and showed more efficient interaction than Bmal2, while Bmal1a exhibited a similar peak of rhythmic expression with Clocka in the brain [108]. Similarly, Clockb was found to contain only one PAS domain, which makes Clockb binding to Bmal1a or Bmalb less effective, and their transcription activities are less effective than Clock1a with Bmal1a or Bmalb counterparts [124]. More importantly, *clockb*^*-/-*^ mutant zebrafish still maintains locomotor rhythmicity, while *clocka*^*-/-*^ mutant zebrafish loses locomotor rhythmicity [110]. Thus, we chose *clocka* and *bmal1a* genes from zebrafish as control groups in this study. The full-length *CLOCK* and *BMAL1* gene sequences of the bottlenose dolphin (d*CLOCK* and d*BMAL1*) and zebrafish (*clocka* and *bmal1a*) were downloaded from the NCBI database. Additionally, cetacean-specific changes in both *CLOCK* (724V, 752P, and 779A) and *BMAL1* (3E, 456P, 461R, and 466T) genes were further confirmed by PCR amplification and direct sequencing. Dolphin tissue samples used in the present study were collected from dead individuals in the wild. The full-length CDS of two genes from dolphin were synthesized by Sangon Biotech (Shanghai) Co., Ltd; the full-length CDS of zebrafish *clocka* and *bmal1a* were obtained by PCR amplification of zebrafish cDNA derived from messenger RNA. These sequences were then individually cloned into pCS2-mCherry or pCS2-EGFP vector (Clontech), expressing CLOCK proteins fused with mCherry and BMAL1 proteins fused with EGFP at the C-terminus, respectively. Next, the Fast Site-Directed Mutagenesis Kit (Tiangen Biotech (Beijing) Co., Ltd.) was utilized for the manipulation of site-directed mutagenesis to construct pCS2-*clocka*-mut-mCherry and pCS2-*bmal1a*-mut-EGFP. All the full-length CDSs were re-amplified by PCR and inserted into pcDNA 3.1 vectors (Invitrogen) for the dual-luciferase reporter assay, respectively. Next, the FLAG tag and HA tag were inserted at the C-terminus of WT or mutant *clocka* and WT or mutant *bmal1a* via site directed mutagenesis for co-immunoprecipitation assays, respectively. The above constructs were verified by DNA sequencing.

HEK293T cells were cultured in DMEM supplemented with 10% fetal bovine serum. For the cellular localization studies, 2 × 10^5^ 293T cells were incubated in the 24-well plates with cell slide inserts on the day before transfection, and polyethylenimine (PEI) reagent was used to transfect DNA constructs in accordance with the manufacturer’s protocol. Fluorescence microscopy was performed on cells fixed with 4% PFA and the nuclei were stained with Hoechst 33342 (Yeasen). A minimum of 60 transfected cells from at least two independent experiments were analyzed for each construct. For the dual-luciferase reporter assay, 293T cells were plated the day before transfection at 2 × 10^5^ cells per well in 24-well plates. Cells were transfected with indicated vectors using PEI. At 48 h after transfection, the cells were lysed, and 20μl of lysate was analyzed for bioluminescence using the dual-luciferase reporter assay system (Promega). All experiments were performed at least three times, and the average values are presented. For immunoprecipitation, 293T cells were cultured in 10-cm Petri dishes until reaching 70–80% confluency and then transfected with plasmid vectors for 2 μg pcDNA-*clocka*-FLAG (WT or mutant) and 2 μg pcDNA-*bmal1a*-HA (WT or mutant). Two days after transfection, extracts were made by adding 400 μl commercial lysis buffer (Swiss Affinibody LifeScience AG) and 4 μl 100× protease inhibitor cocktail (Beyotime Biotechnology) to harvested cells for 1 hour on ice, and then centrifuged at 13,000 rpm for 15 min at 4 °C. In total, 10% of the cell extracts were retained for input. The supernatant was incubated with anti-HA nanobody agarose beads (KTSM1305, AlpalifeBio, Shenzhen, China) overnight at 4 °C in a rocking platform at low speed. After washing three times, the precipitates were resuspended in SDS–PAGE sample buffer, boiled at 95°C for 10 min, and run on a Ultragel SDS–PAGE gel (Applygen, Beijing, China). Immunoblotting was performed using HRP-conjugated anti-HA (HRP-81290, Proteintech, at 1:10,000 dilution) or mouse monoclonal anti-FLAG (F1804, Sigma, at 1:1000 dilution) antibodies, and an anti-mouse secondary antibody (SA00001-1, Proteintech, at 1: 10,000 dilution).

### Zebrafish culture

All zebrafish lines were housed on a 14 h/10 h light-dark schedule in dechlorinated water at 28.5 °C and routine husbandry was performed by the zebrafish facility of Nanjing Normal University. Embryos were obtained by natural crosses and were staged according to standard developmental conditions, and fertilized eggs were raised at 28.5 °C.

### *In vivo* functional assays

The constructed plasmids, including *mCherry*, WT-*clocka*, *clocka*-mut, WT-*bmal1a* and *bmal1a*-mut, were linearized with SacII and then transcribed into mRNAs using mMESSAGE mMACHINE™ SP6 Transcription Kit (Thermo Fisher Scientific, Waltham, MA, USA), respectively. mRNA products were diluted to 200 ng/μl with DEPC water for microinjection. The mRNA products were microinjected into the one-cell-stage zebrafish embryos using the ASI pressure injection system. The zebrafish embryos injected with WT or mutant mRNA were all collected at the 72 hpf developmental stage, i.e., ZT 1–ZT 2 (Fig 3A), when *clocka* showed an increased expression [125,126]. Moreover, previous studies suggest that circadian systems have played a regulatory role before 72 hpf; specifically that zebrafish embryos become light responsive around 5 hpf [127] while the onset of rhythms in the pineal gland occurs from 24 to 36 hpf [128,129] and then sleep and activity rhythms are gradually established [130,131]. Collected embryos were then stored in TRIzol reagent (Sangon Biotech Co., Ltd. Shanghai, China) for semiquantitative reverse transcription-polymerase chain reaction (RT-PCR) and transcriptome sequencing. Zebrafish lines that transiently expressing mCherry in *clocka*^*−/−*^ and in *bmal1a*^*−/−*^ zebrafish were used to examine the effects of transgenic system on zebrafish morphology and gene expression efficiency. Semiquantitative RT-PCR was performed using first-strand cDNA synthesized from total RNA samples (HiScript III 1st Strand cDNA Synthesis Kit, Vazyme Biotech co., Ltd) and Taq Pro Universal SYBR qPCR Master Mix (Vazyme Biotech co., Ltd). The results showed that both the *clocka* and *bmal1a* genes were highly expressed in the transgenic lines (S16 Fig).

### RNA-seq-based transcriptome analysis

Equal quantities of total RNAs from three replicate samples in each group (containing 15 zebrafish larvae) were prepared for RNA sequencing. The larvae were collected and snap-frozen in liquid nitrogen. The whole body of frozen larvae were ground to homogeneity with a pipet tip and stored at −80 °C. Total RNA was extracted using the TRIzol reagent following the manufacturer’s instructions. The quality and quantity of RNA were measured using agarose gel electrophoresis and a Nanodrop1000 (Thermo Fisher Scientific, Waltham, MA USA). RNA sequencing and analysis were performed by Novogene (Beijing, China), as previously described [132].

### Larval zebrafish behavioral tracking

For behavioral assays, the zebrafish larvae transiently overexpressing *clocka*-mut, WT-*clocka*, *bmal1a*-mut, and WT-*bmal1a* were generated on the wild-type zebrafish background (AB-type) as previously described. As a control group, AB-type zebrafish embryos were injected with DEPC water alone. At 5 days postfertilization (dpf), zebrafish larvae were placed into individual wells of a 48-round-well plate filled with 1.5 ml of embryo water per well and tracked for 3 consecutive days under a 14 h/10 h light-dark schedule (lights on, 09:00; lights off, 23:00) in ViewPoint ZebraBoxes of the automated video tracking system (Viewpoint Life Sciences, France). The 48-well plate was under illumination with white LEDs simulating the light-dark schedule, and fish movement was captured through an infrared camera. The sleep data were recorded and analyzed using ZebraLab software (Version 2.3.1, ViewPoint, France) with the following parameters: detection threshold, 20; burst, 25; freeze, 4; bin size, 60 s. The sleep and wake behaviors of each zebrafish larva were then analyzed using MATLAB (Version R2021a) scripts as described in David Prober’s lab [133].

### Statistical analysis

Statistical calculations were performed using the GraphPad Prism 8 software. The D’Agostino & Pearson normality and Shapiro–Wilk tests were used to check the data distribution. If data were normally distributed, N-way ANOVA (alpha = 0.05) was used with correction for multiple comparisons using Holm–Sidak multiple posthoc test. If non-parametric, the Kruskal–Wallis test was used with correction for multiple comparisons using Dunn–Sidak (alpha = 0.05). All experiments were independently repeated at least three times. All data were expressed as the mean ± SEM. The labels “ns,” “*,” “**,” “***”, and “****” indicate not significant, *p* < 0.05, *p* < 0.01, *p* < 0.001, and *p* < 0.0001, res*p*ectively.

### Data and resource availability

The raw transcriptome data have been deposited at NCBI under the project accession number PRJNA901278. Newly sequenced genes have been registered in GenBank under the reference numbers OR712815–OR712886. All data were included in supplementary materials for readers to validate.

## Supporting information

S1 Fig Regression analyses between root-to-tip ω and the SWS/TST ratio. (PDF)

S2 Fig Cetacean-specific amino acid substitutions identified in the eight canonical circadian clock gene.
(PDF)

S3 Fig Three-dimensional structure of WT-Clocka, Clocka-mut, WT-Bmal1a, and Bmal1a-mut proteins.
(PDF)

S4 Fig Surface diagram of the docking model and their interfacing residues for the WT-Clocka and WT-Bmal1a complex and the Clocka-mut and Bmal1a-mut complex.
WT-Clocka, yellow; WT-Bmal1a, bule; Clocka-mut, orange; WT-Bmal1a, steel bule; hydrogen bond interaction, dotted line.
(PDF)

S5 Fig No significant morphological changes in 72hpf *clocka* ^*-/-*^ zebrafish after injection of *mCherry*-mRNA, WT-*clocka*-mRNA and *clocka*-mut-mRNA, respectively.
(PDF)

S6 Fig No significant morphological changes in 72hpf *bmal1a* ^*-/-*^ zebrafish after injection of *mCherry*-mRNA, WT-*bmal1a*-mRNA and *bmal1a*-mut-mRNA, respectively.
(PDF)

S7 Fig GO enrichment of 407 DEGs in the *clocka*-mut overexpression group versus the WT-*clocka* group.
(PDF)

S8 Fig KEGG enrichment of 407 DEGs in the *clocka*-mut overexpression group versus the WT-*clocka* group.
(PDF)

S9 Fig GO enrichment of 590 DEGs in the *bmal1a*-mut overexpression group versus the WT-*bmal1a* group.
(PDF)

S10 Fig KEGG enrichment of 590 DEGs in the *bmal1a*-mut overexpression group versus the WT-*bmal1a* group.
(PDF)

S11 Fig Number of DEGs identified in the circadian oscillating gene sets from CircaKB database (https://cdsic.njau.edu.cn/CircaKB).
(PDF)

S12 Fig KEGG enrichment of 83 circadian oscillating DEGs in the *clocka*-mut overexpression group versus the WT-*clocka* group.
(PDF)

S13 Fig KEGG enrichment of 102 circadian oscillating DEGs in the *bmal1a*-mut overexpression group versus the WT-*bmal1a* group.
(PDF)

S14 Fig The phylogenetic topology of eight canonical circadian clock gene based on nucleotide sequences.
Numbers above the branches represent the ML bootstrap values and the Bayesian posterior probabilities.
(PDF)

S15 Fig The phylogenetic topology of eight canonical circadian clock gene based on amino acid sequences.
Numbers above the branches represent the ML bootstrap values and the Bayesian posterior probabilities.
(PDF)

S16 Fig qRT-PCR analysis of *clocka* (A) and *bmal1a* (B) in 72hpf zebrafish after transient overexpression. Statistical analysis was performed using student’s *t-*test. *p < 0.05; **p < 0.01; ***p < 0.0001.
(PDF)

S1 Table Statistics for amplified exons of eight circadian clock genes for nine cetaceans.
(DOCX)

S2 Table Sequence data used in present study.
(DOCX)

S3 Table The overall evolutionary distance between amino acid sequences for eight circadian clock genes.
(DOCX)

S4 Table Results of PAML branch-site model analysis of circadian genes in all-mammals dataset.
(DOCX)

S5 Table Summary of branch-site positive selection analysis using the aBSREL method implemented in HyPhy v2.5.58.
(DOCX)

S6 Table Summary of branch-site positive selection analysis using the BUSTED method implemented in HyPhy v2.5.58.
(DOCX)

S7 Table The SWS/TST ratios in mammals.
(DOCX)

S8 Table The PGLS analyses between the root-to-tip ω and SWS/TST in all mammals. (DOCX)

S9 Table Functional effect of USWS group-specific amino acid changes.
(DOCX)

S10 Table Functional effect of cetacean-specific amino acid replacements.
(DOCX)

S11 Table Identified site changes link to protein function.
(DOCX)

S12 Table Structural alignments for the WT and mutant.
(DOCX)

S13 Table Structural alignments for circadian clock proteins from representative species.
(DOCX)

S14 Table Molecular docking results.
(DOCX)

S15 Table Detail information on sample collection for PCR amplification and sequencing.
(DOCX)
