# Supplementary figures and images for: Evolution of canonical circadian clock genes underlies unique sleep strategies of marine mammals for secondary aquatic adaptation
Source: PLoS Genet. 2025 Mar 18;21(3):e1011598. doi: 10.1371/journal.pgen.1011598 (PMC11919277; doi:10.1371/journal.pgen.1011598)

***BMAL1***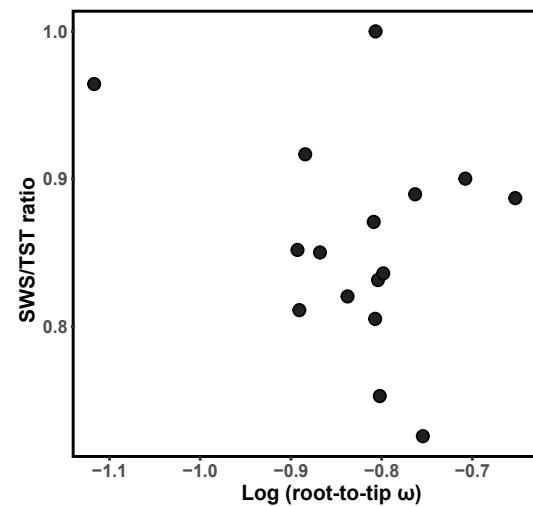***CLOCK***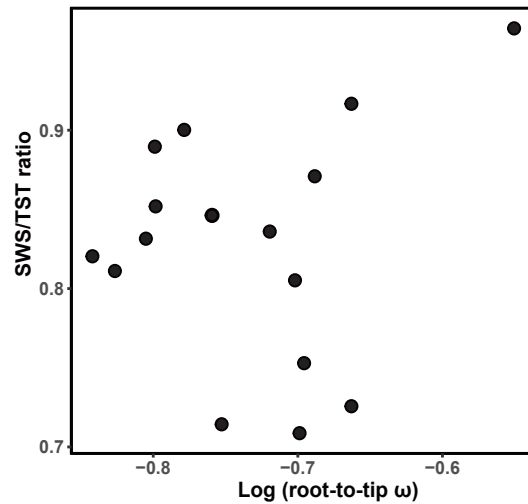***CRY1***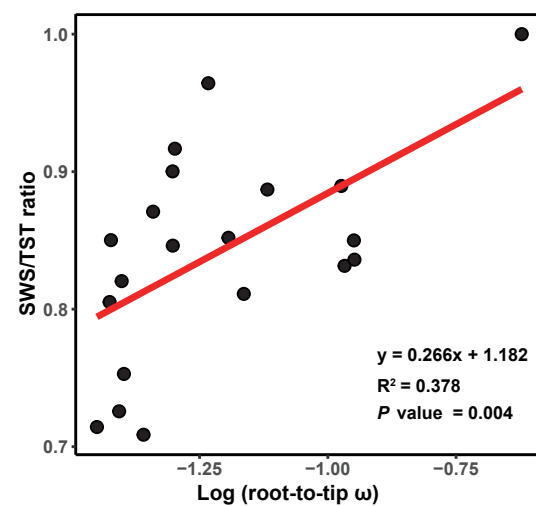***CRY2***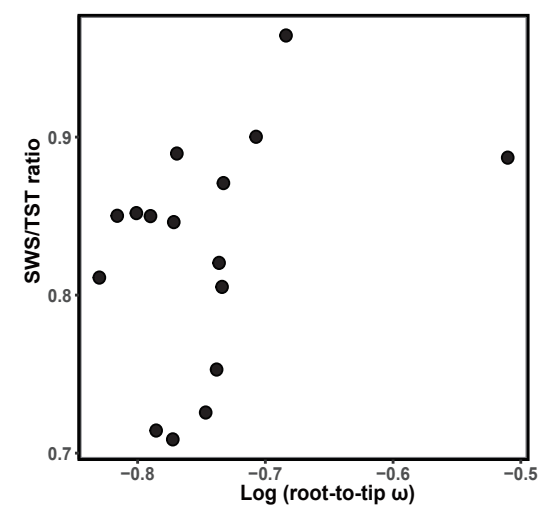***NPAS2***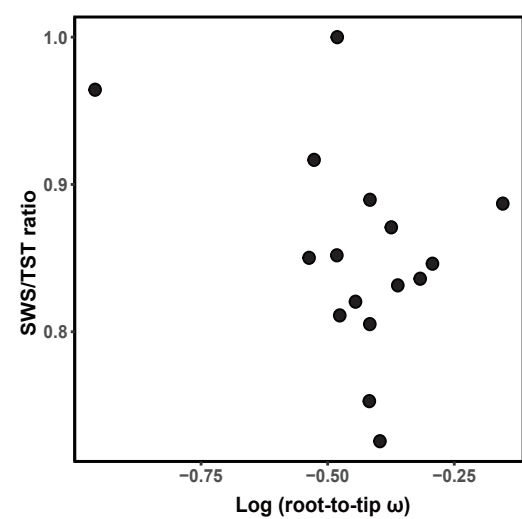***PER1***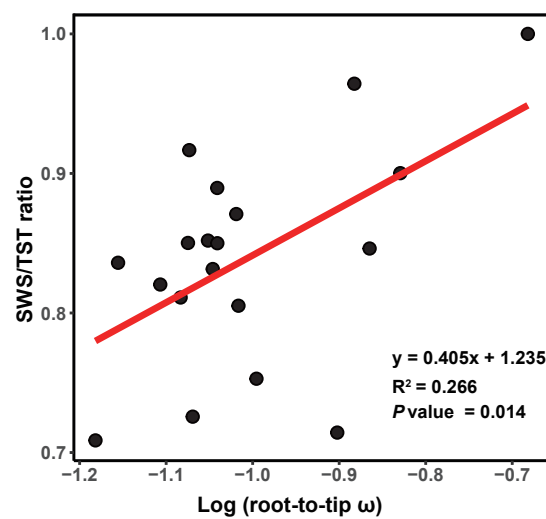***PER2***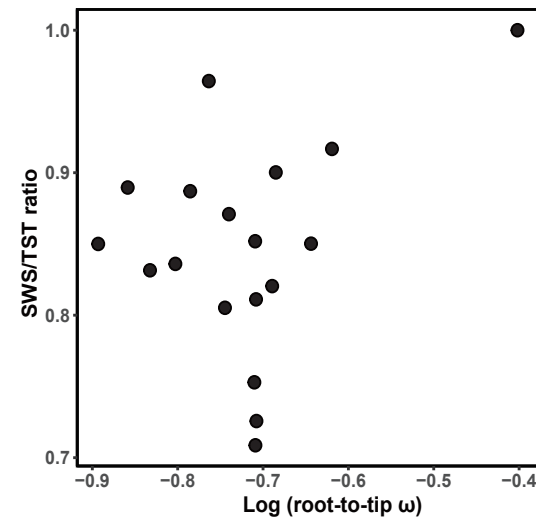***PER3***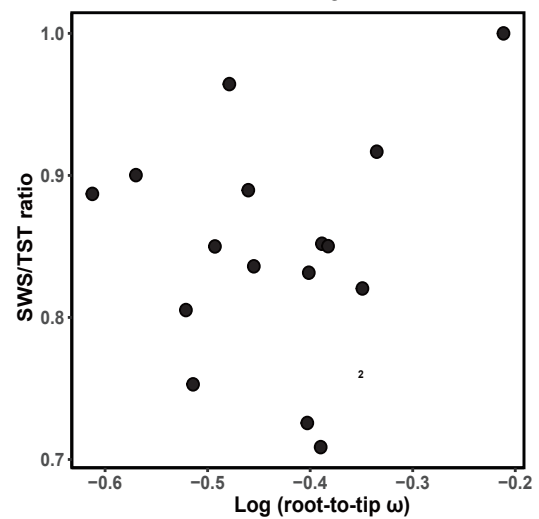

Supplement: S1 Fig — (PDF) [file pgen.1011598.s001.pdf]

**BMAL1**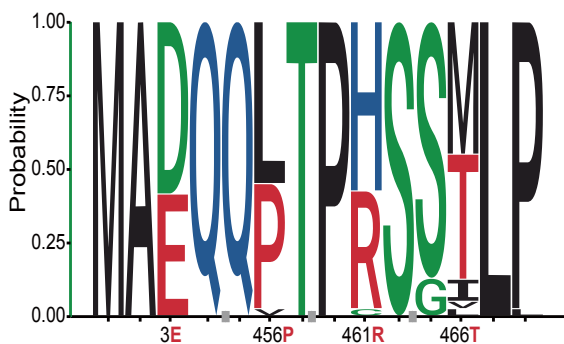**CLOCK**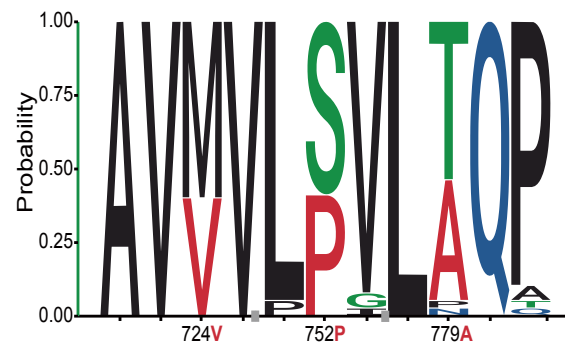**PER1**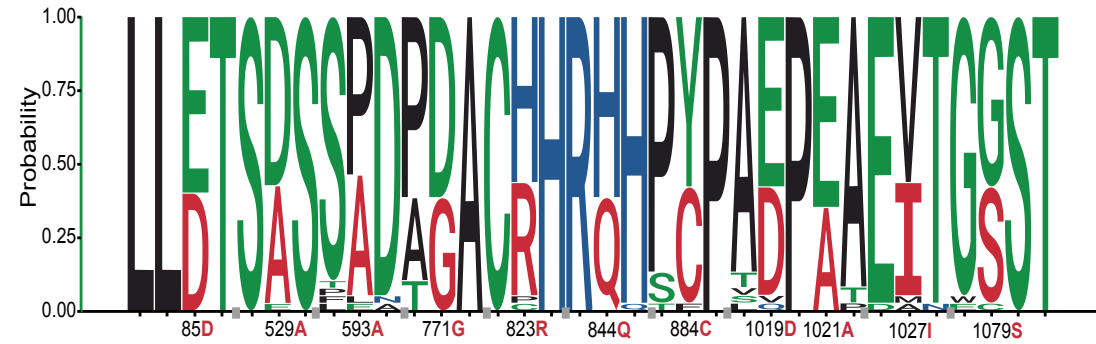**CRY1**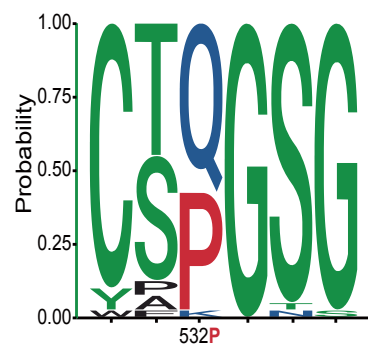**CRY2**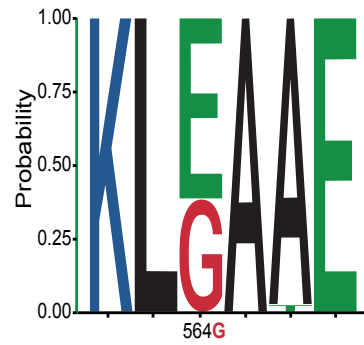**PER2**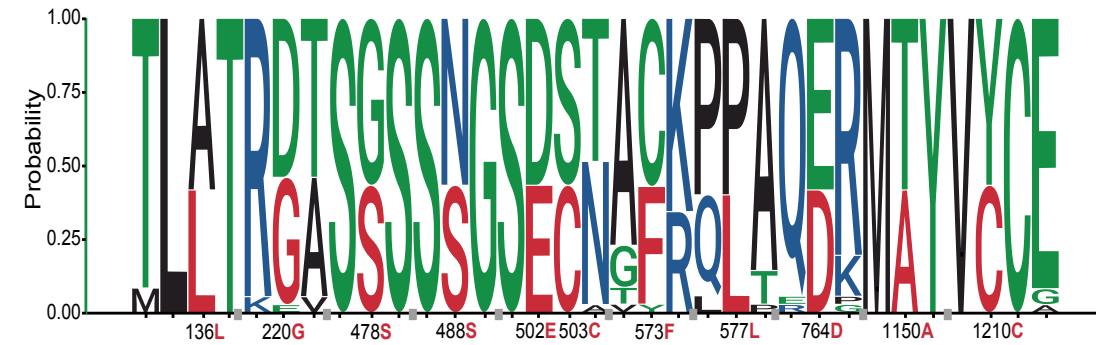**NPAS2**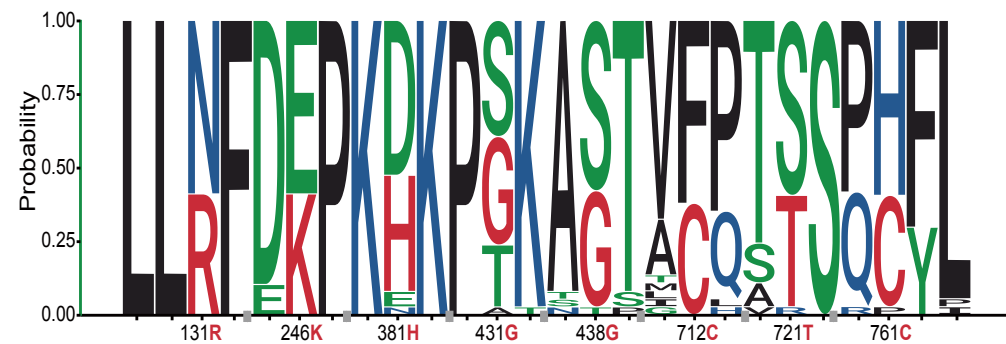**PER3**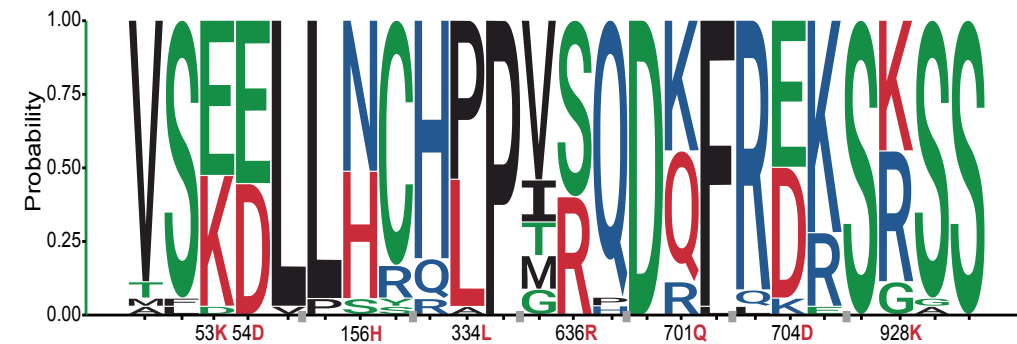

Supplement: S2 Fig — (PDF) [file pgen.1011598.s002.pdf]

**WT-Clocka**

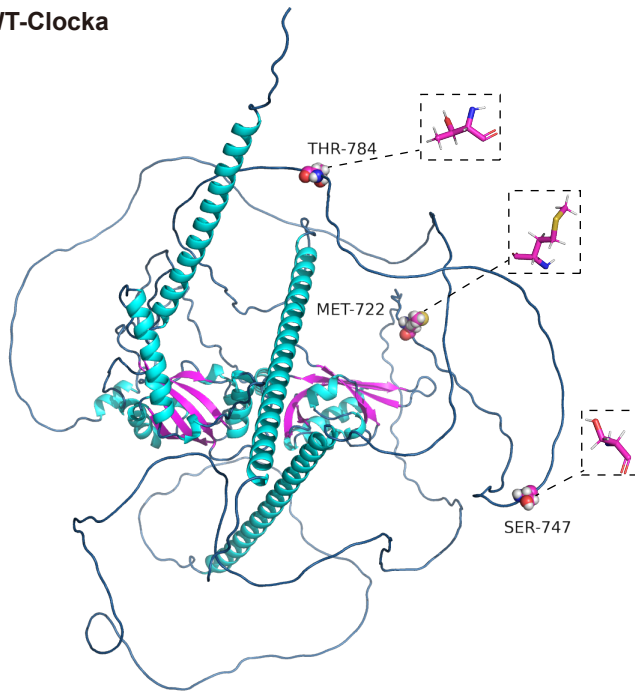

**Clocka-mut**

**TM-score = 0.4994**

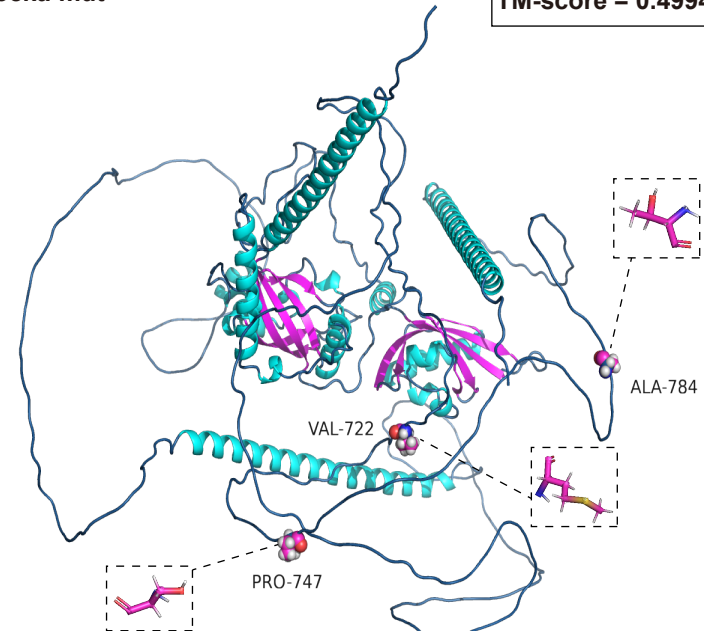

**WT-Bmal1a**

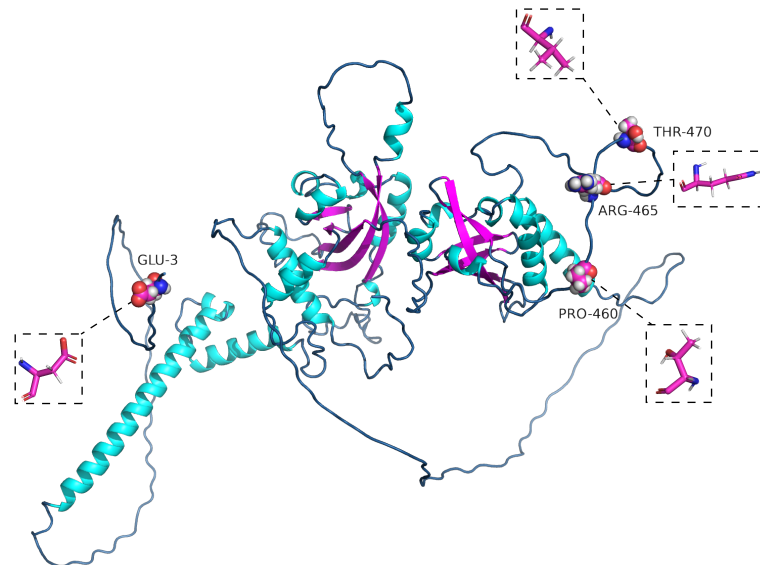

**Bmal1a-mut**

**TM-score = 0.7923**

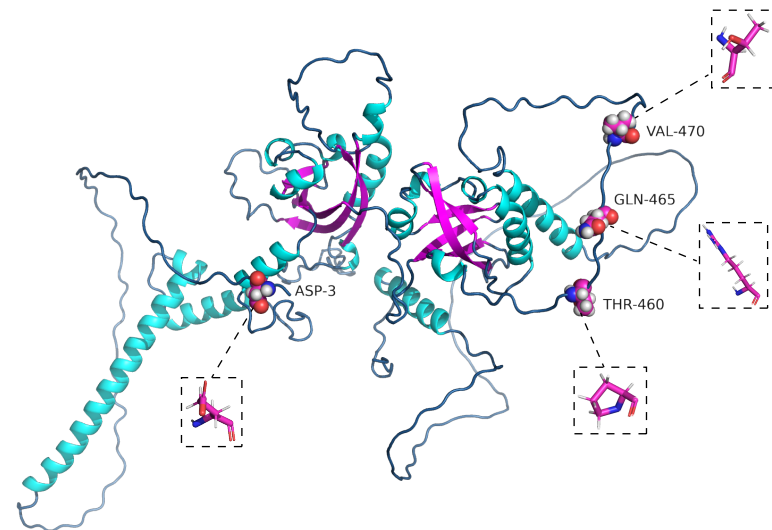

Supplement: S3 Fig — (PDF) [file pgen.1011598.s003.pdf]

***clocka*<sup>-/-</sup>**

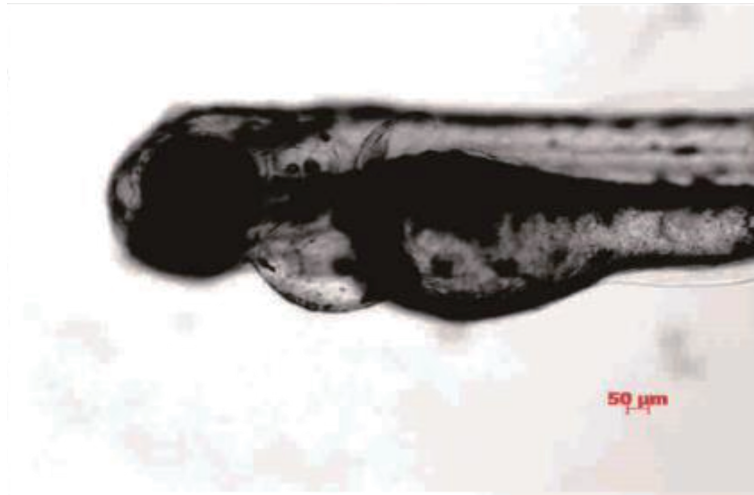

***clocka*<sup>-/-</sup> + *mcherry***

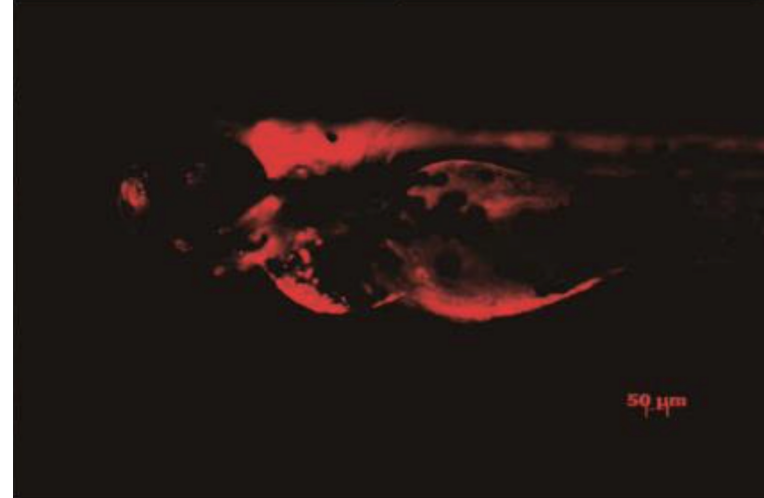

***clocka*<sup>-/-</sup> + WT-*clocka***

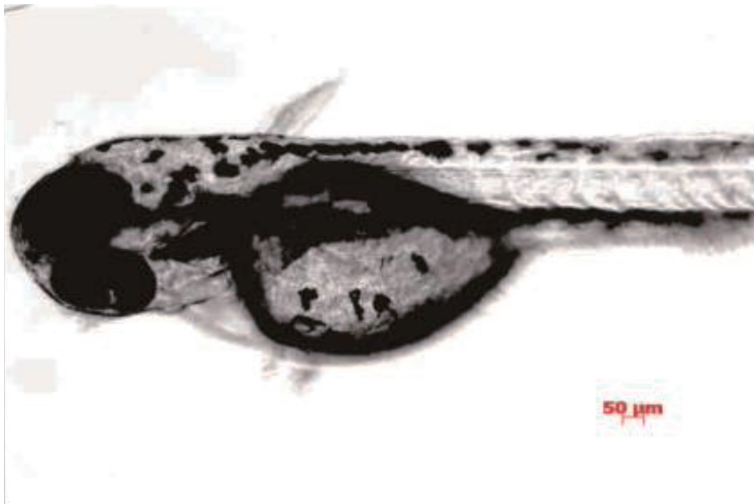

***clocka*<sup>-/-</sup> + *clocka*-mut**

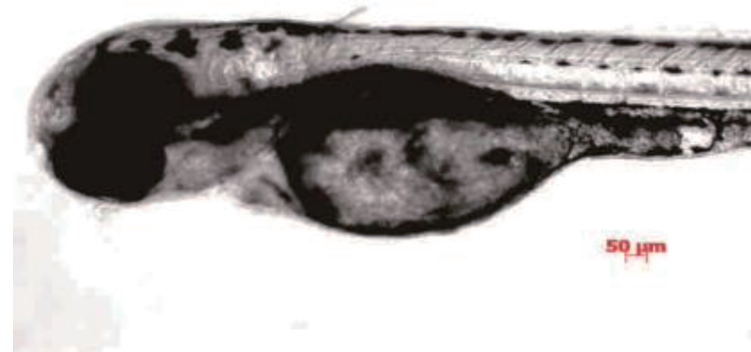

Supplement: S5 Fig — (PDF) [file pgen.1011598.s005.pdf]

***bmal1a*<sup>-/-</sup>**

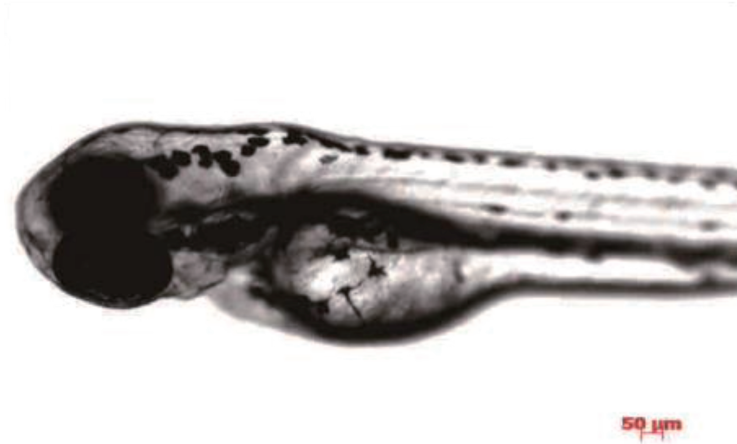

***bmal1a*<sup>-/-</sup> + mcherry**

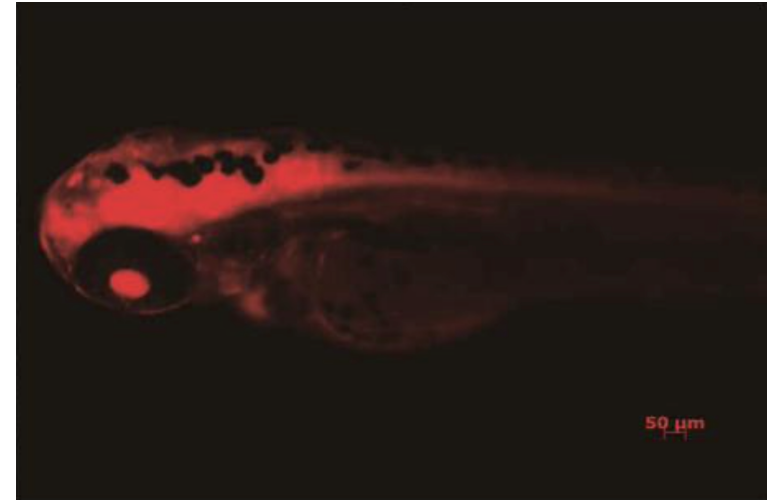

***bmal1a*<sup>-/-</sup> + WT-*bmal1a***

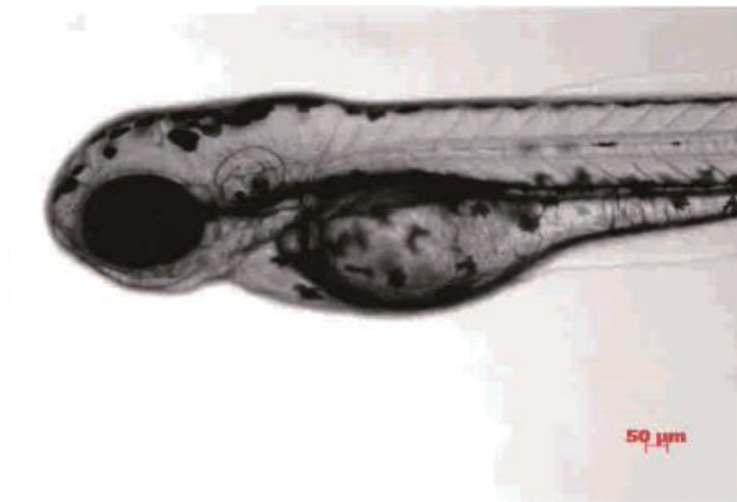

***bmal1a*<sup>-/-</sup> + *bmal1a*-mut**

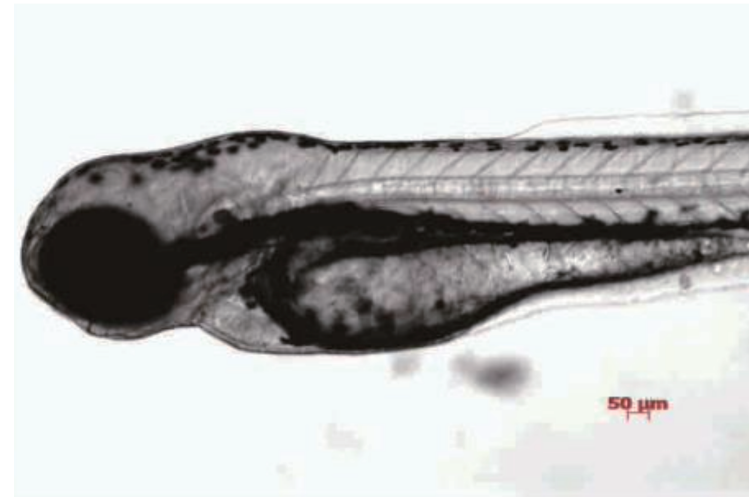

Supplement: S6 Fig — (PDF) [file pgen.1011598.s006.pdf]

# WT-clocka vs. clocka-mut

Pathway

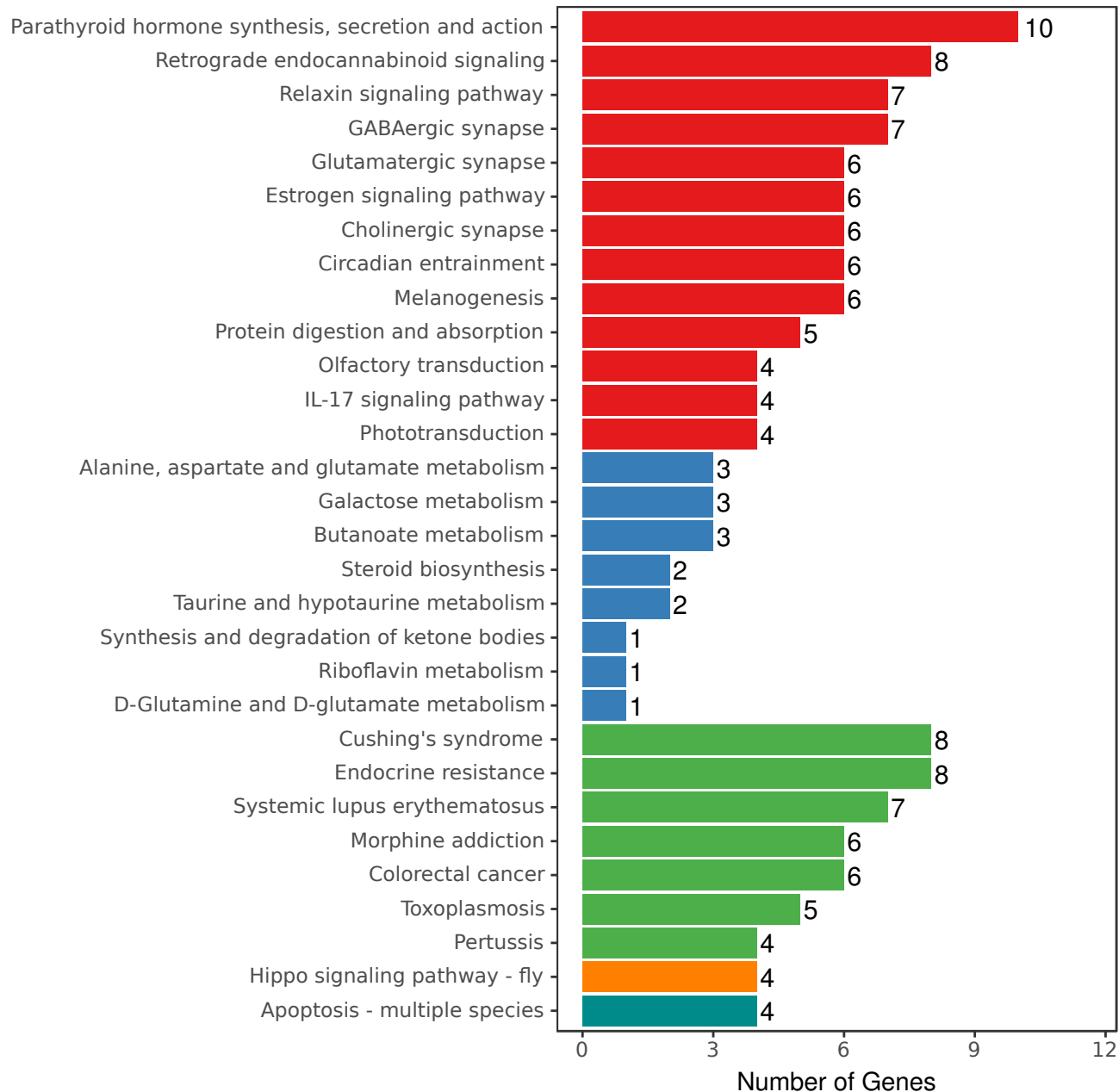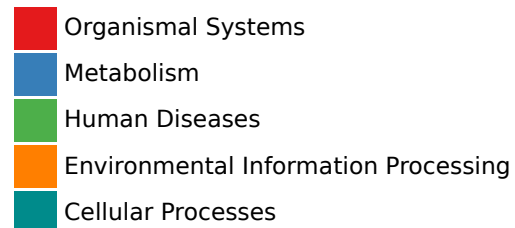

Supplement: S8 Fig — (PDF) [file pgen.1011598.s008.pdf]

# WT-bmal1a vs. bmal1a-mut

GO Term

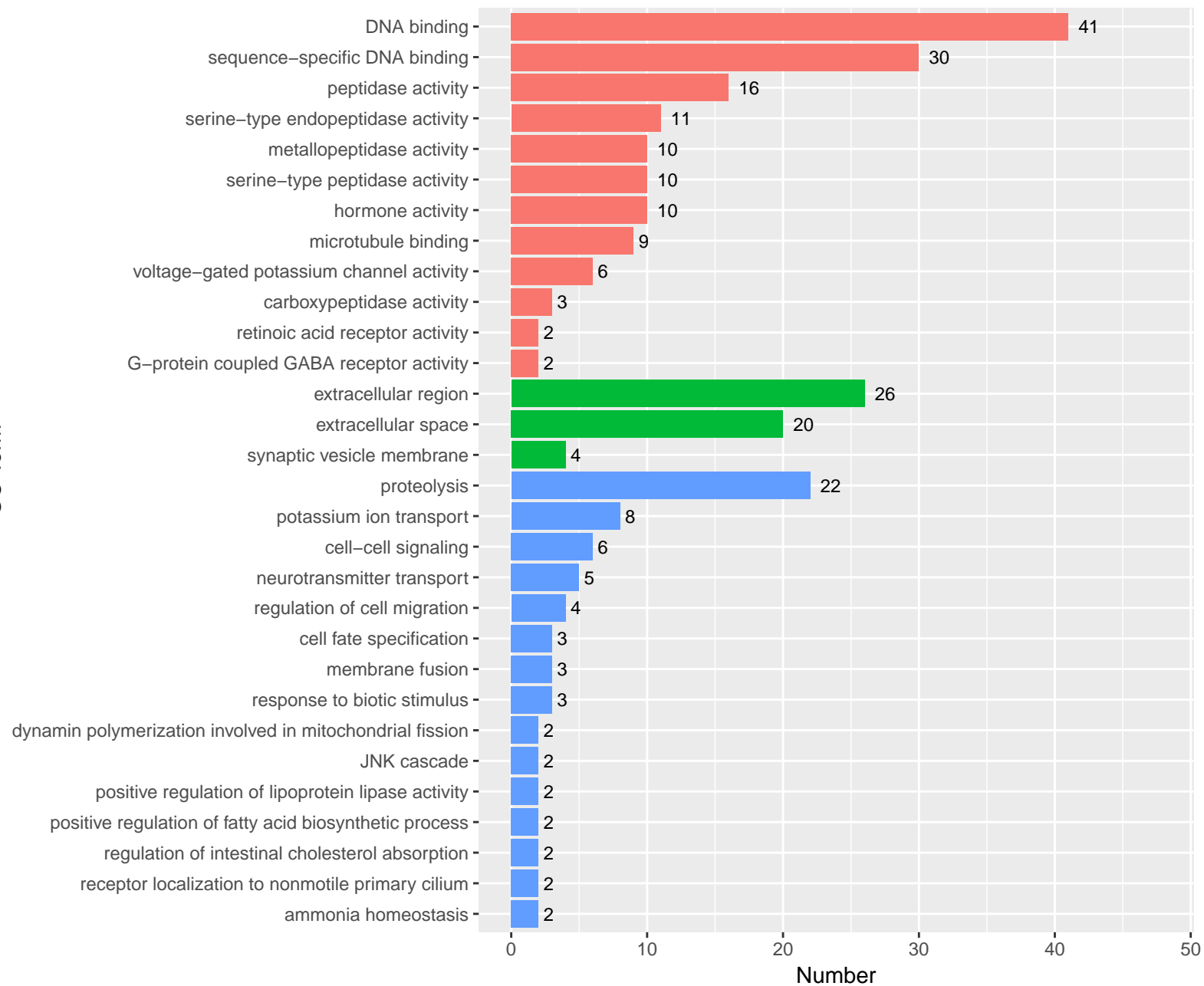

Ontology

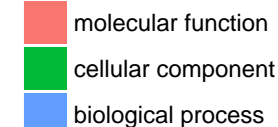

Supplement: S9 Fig — (PDF) [file pgen.1011598.s009.pdf]

# WT-bmal1a vs. bmal1a-mut

Pathway

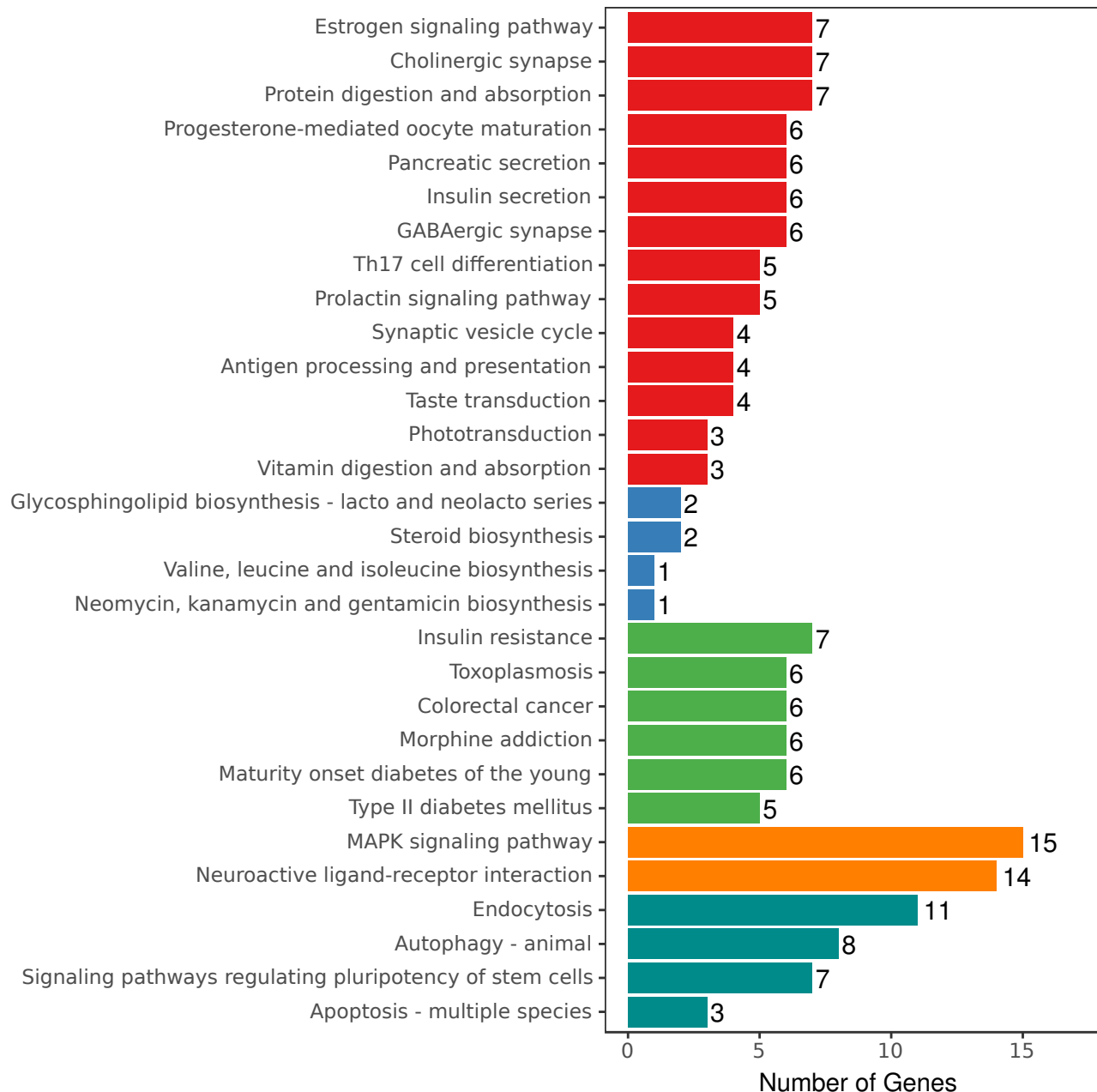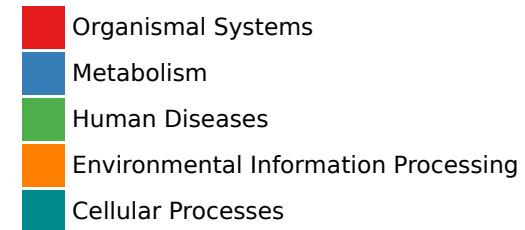

Supplement: S10 Fig — (PDF) [file pgen.1011598.s010.pdf]

407 DEGs for *clocka*-mut vs WT-*clocka*

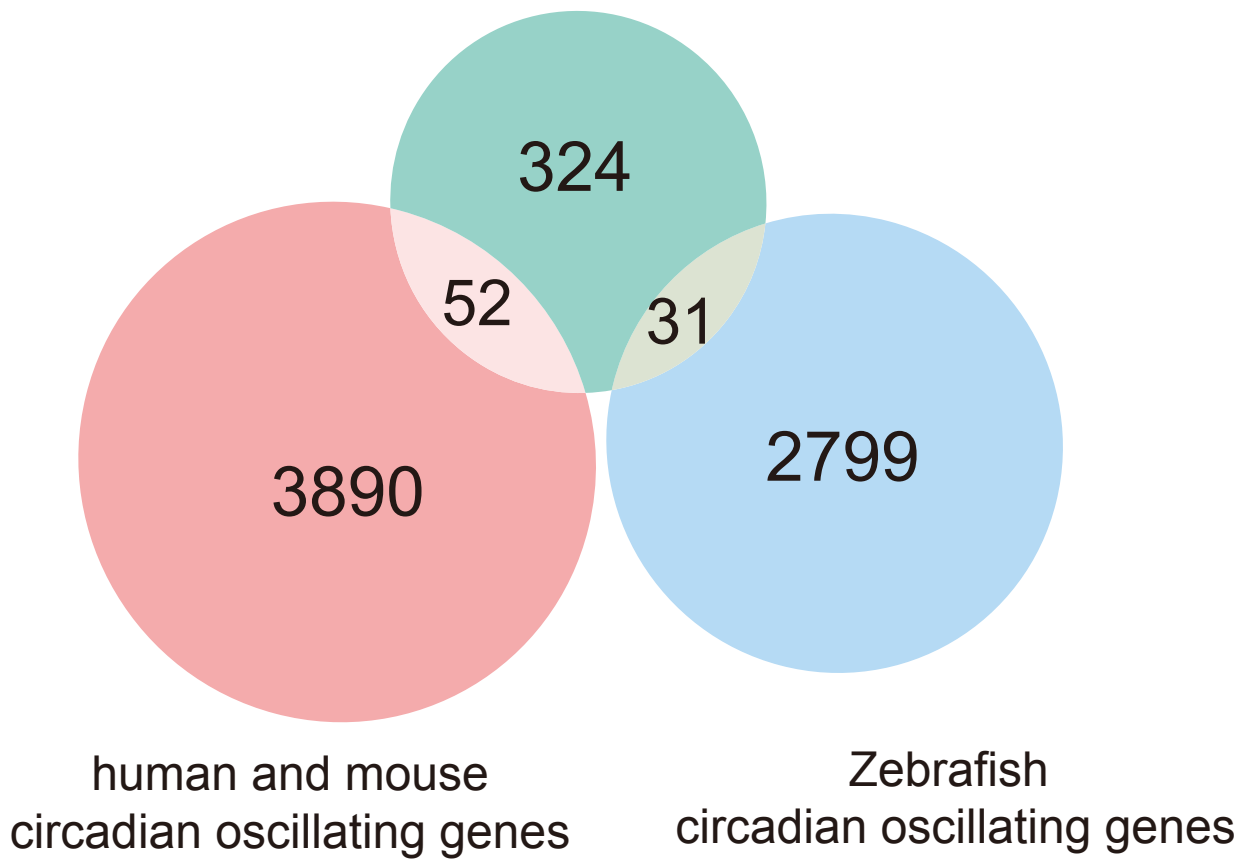

590 DEGs for *bmal1a*-mut vs WT-*bmal1a*

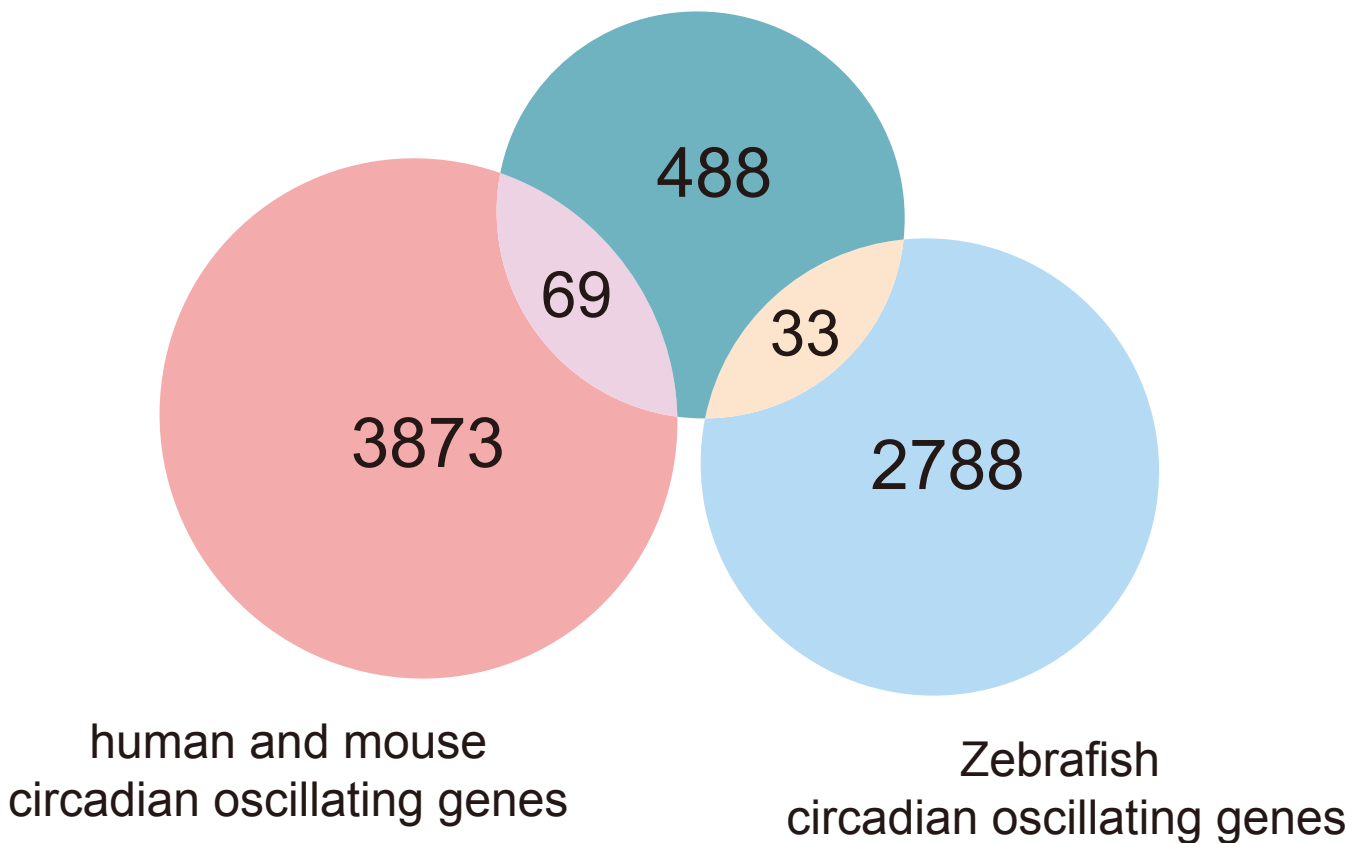

Supplement: S11 Fig — (PDF) [file pgen.1011598.s011.pdf]

# Top 25 of KEGG Enrichment

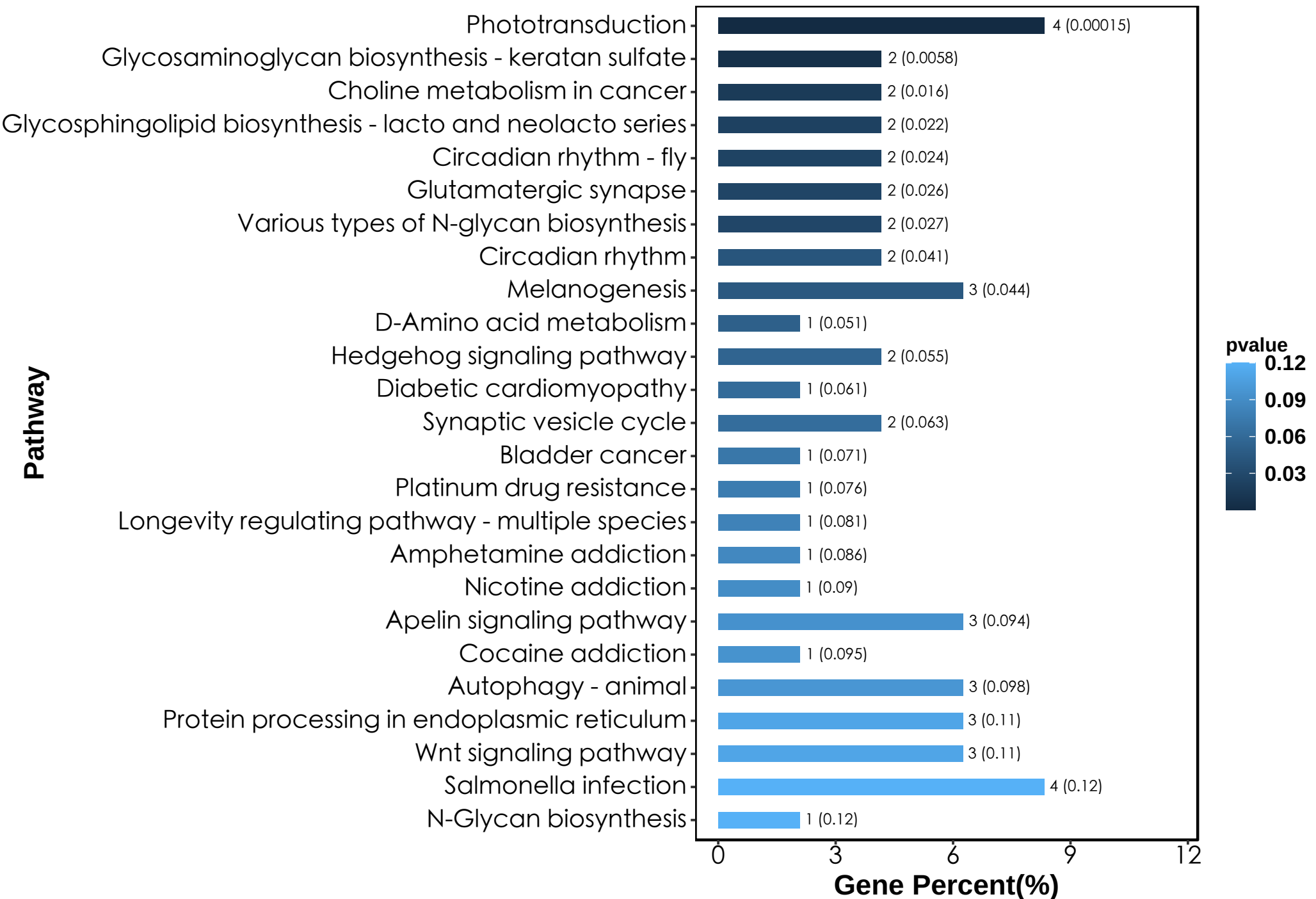

Supplement: S12 Fig — (PDF) [file pgen.1011598.s012.pdf]

# Top 25 of KEGG Enrichment

Pathway

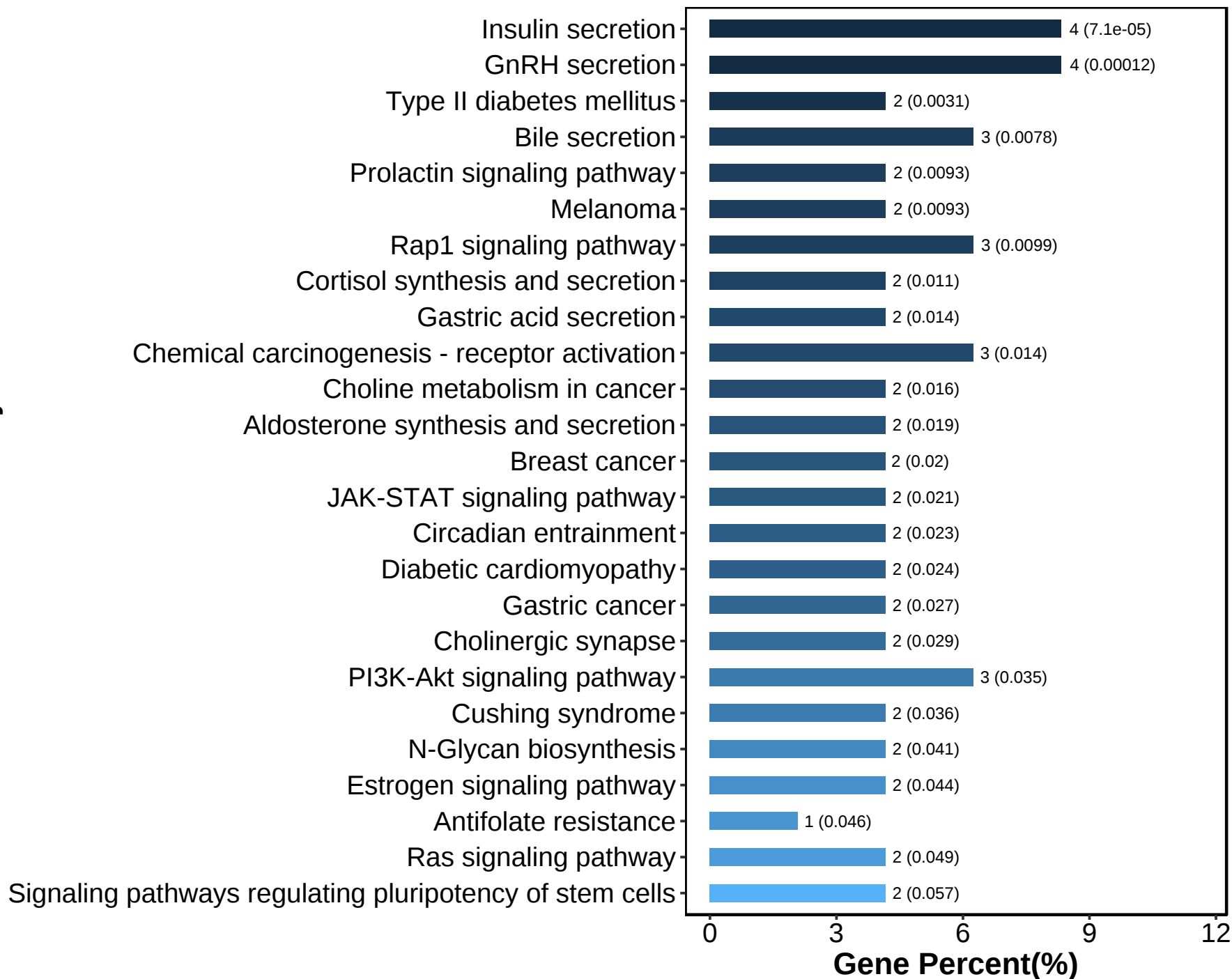

Supplement: S13 Fig — (PDF) [file pgen.1011598.s013.pdf]

BMAL1

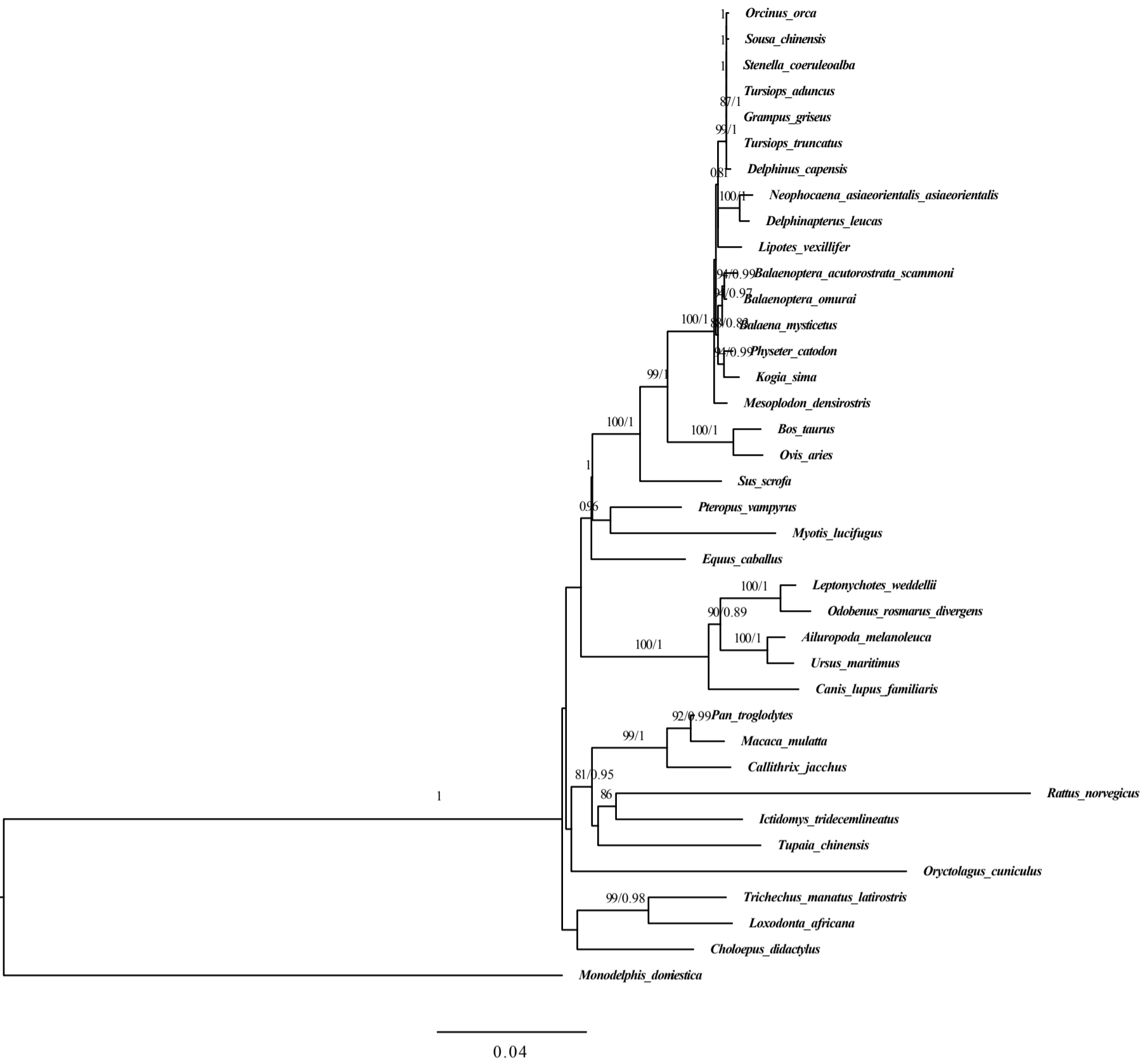

CLOCK

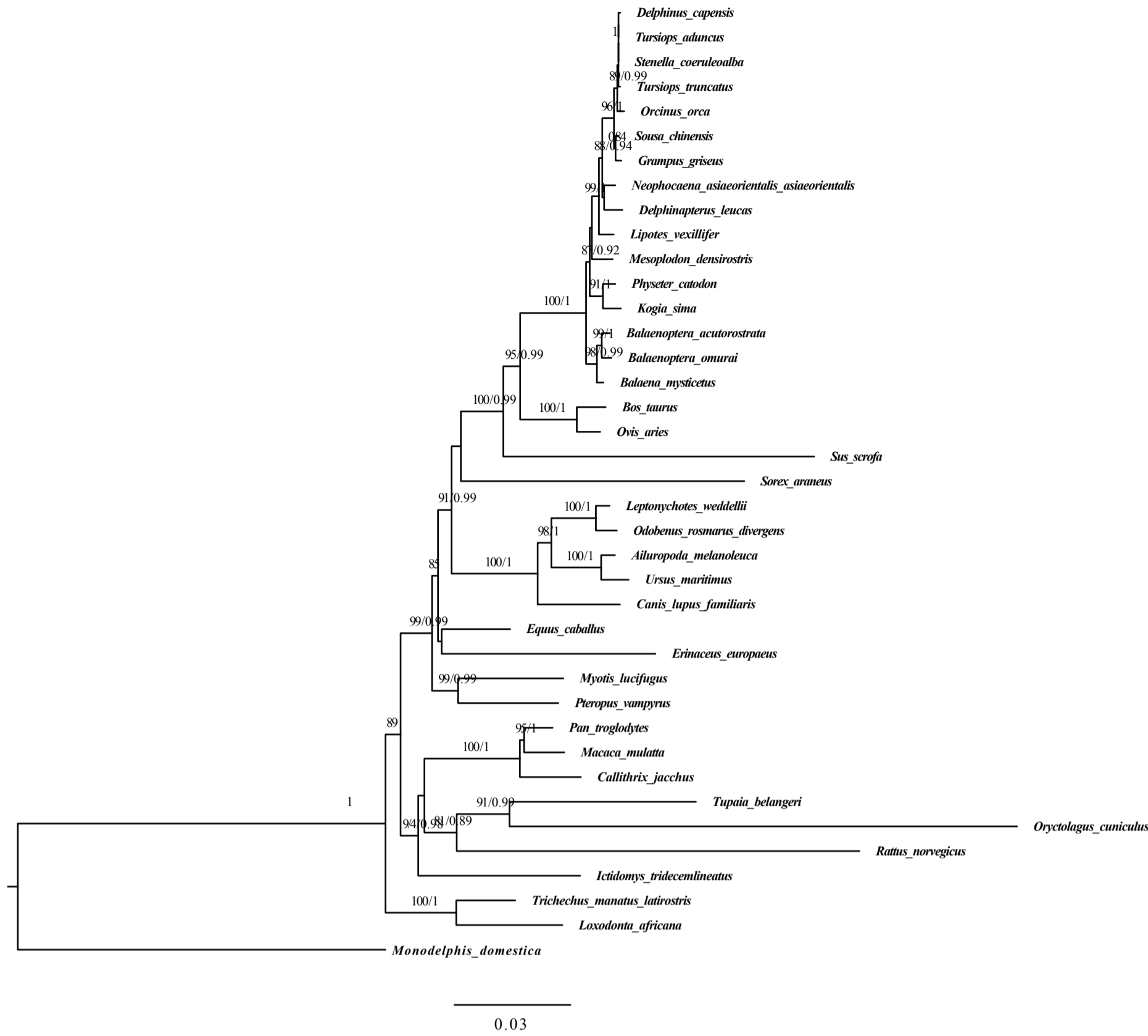

CRY1

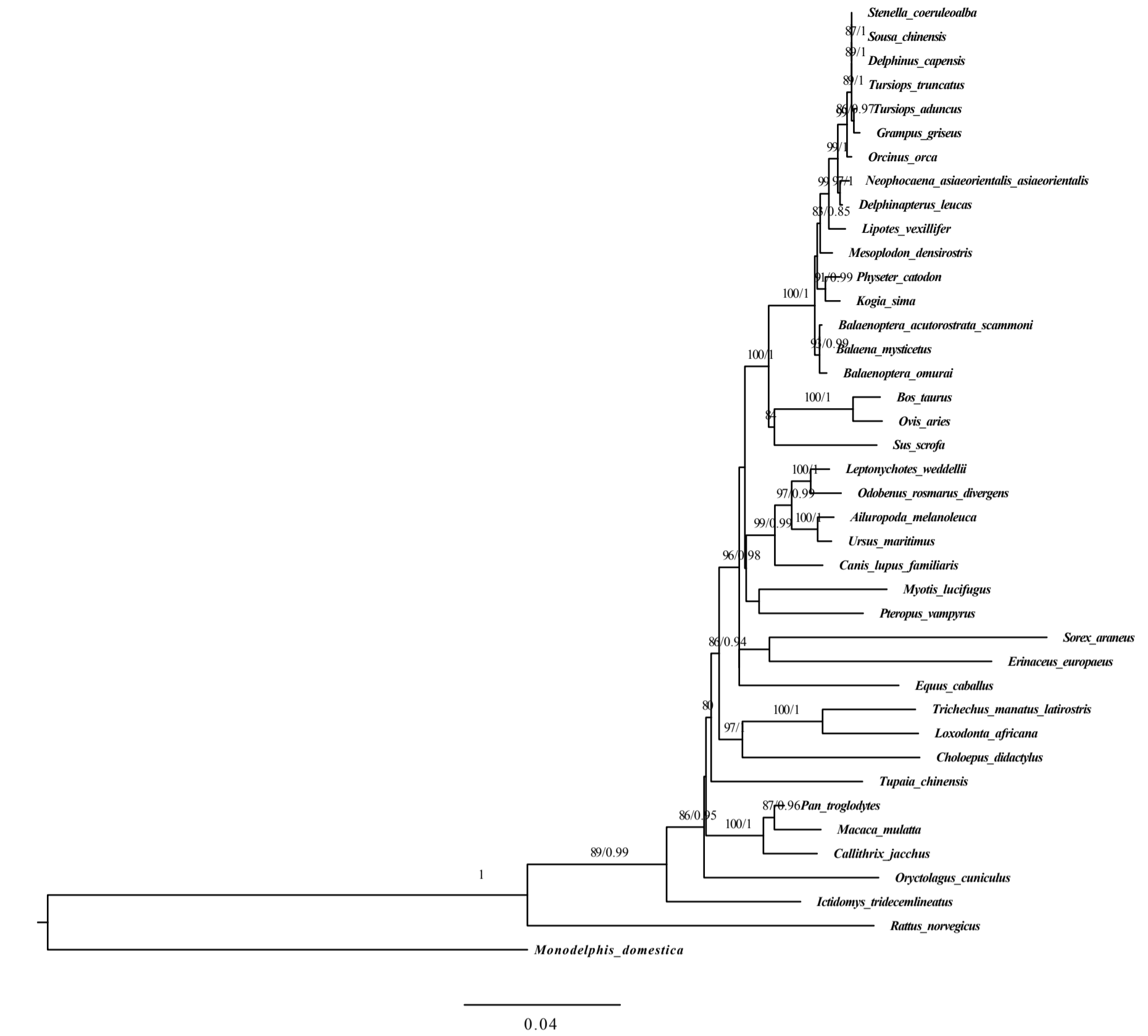

CRY2

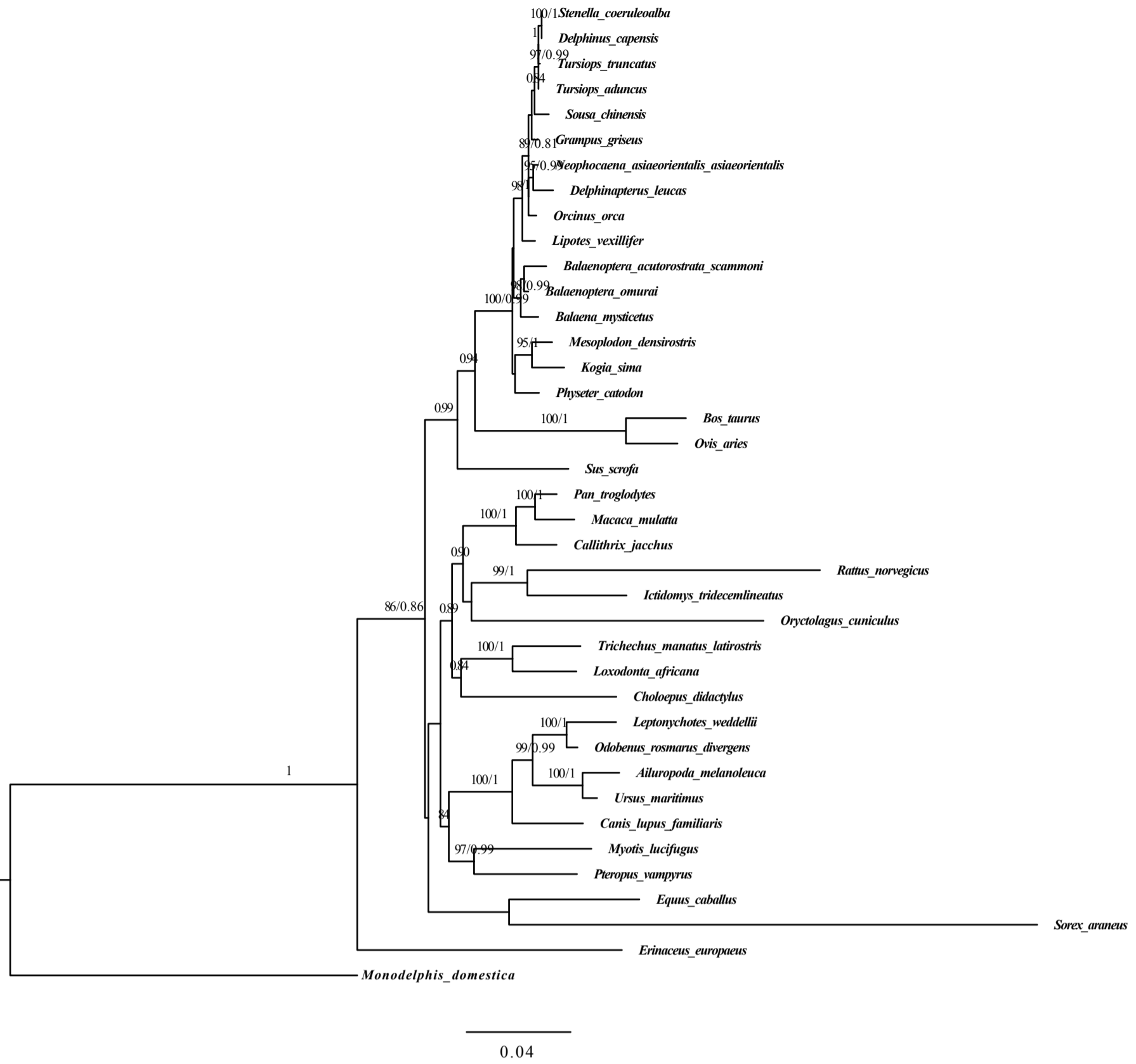

NPAS2

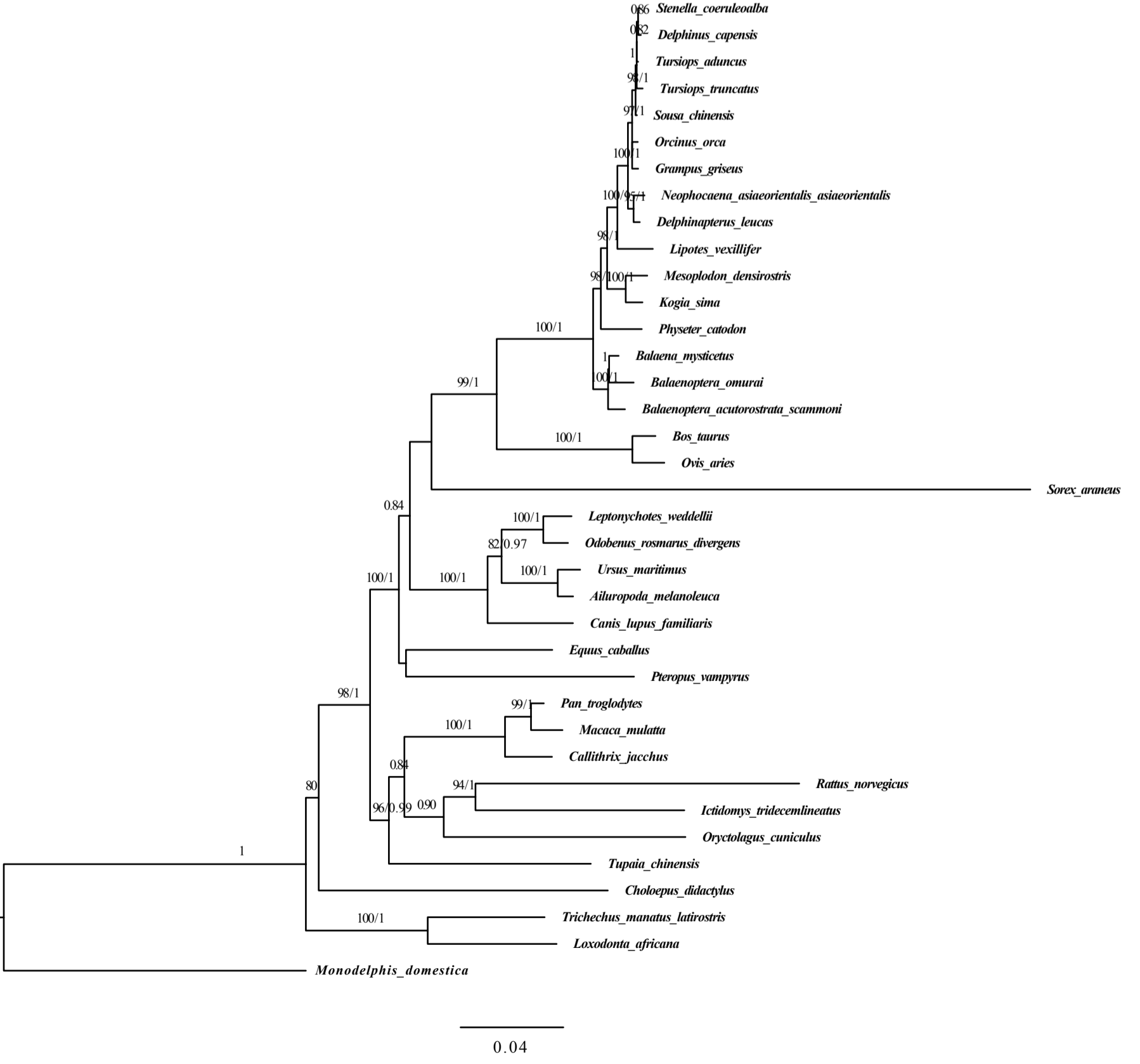

PER1

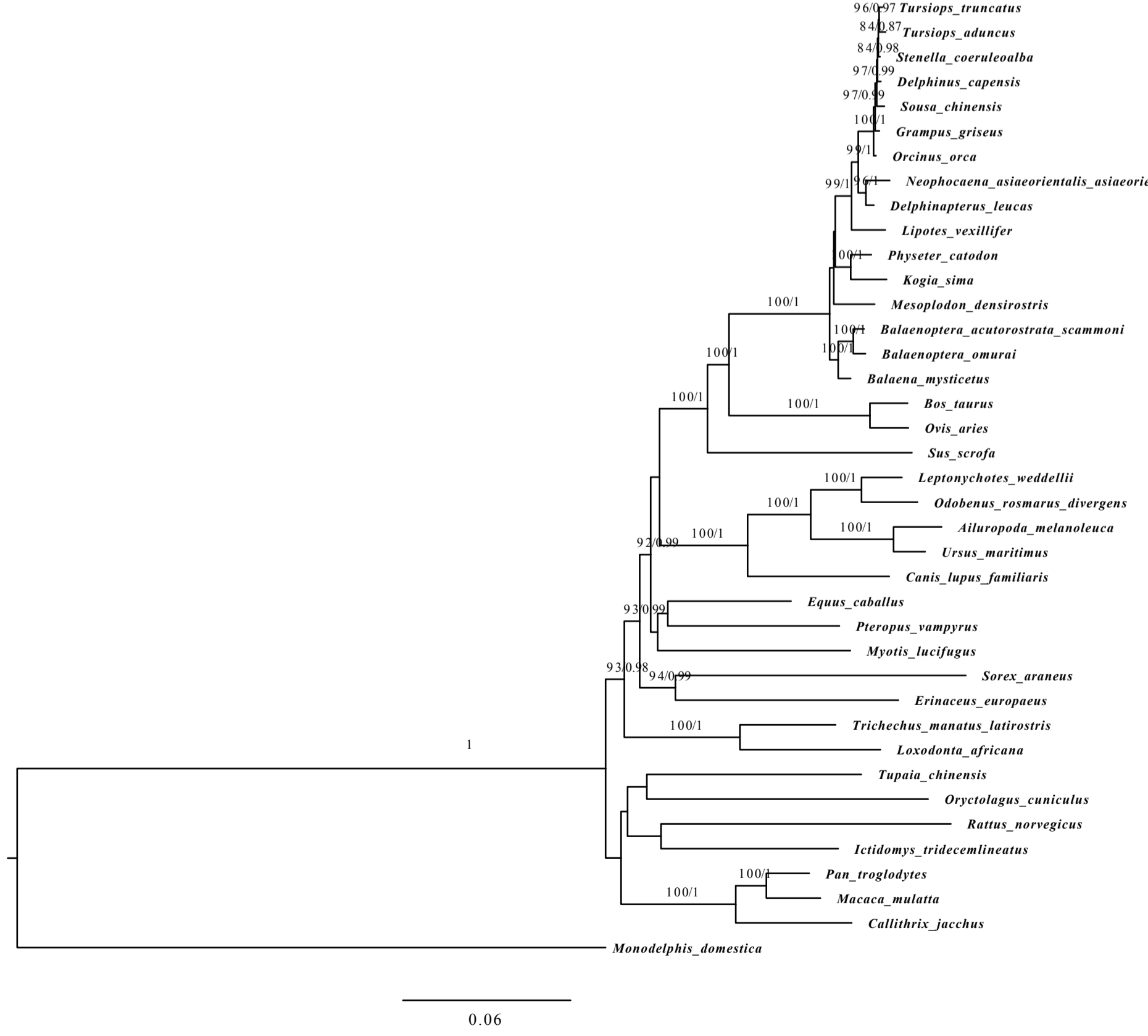

PER2

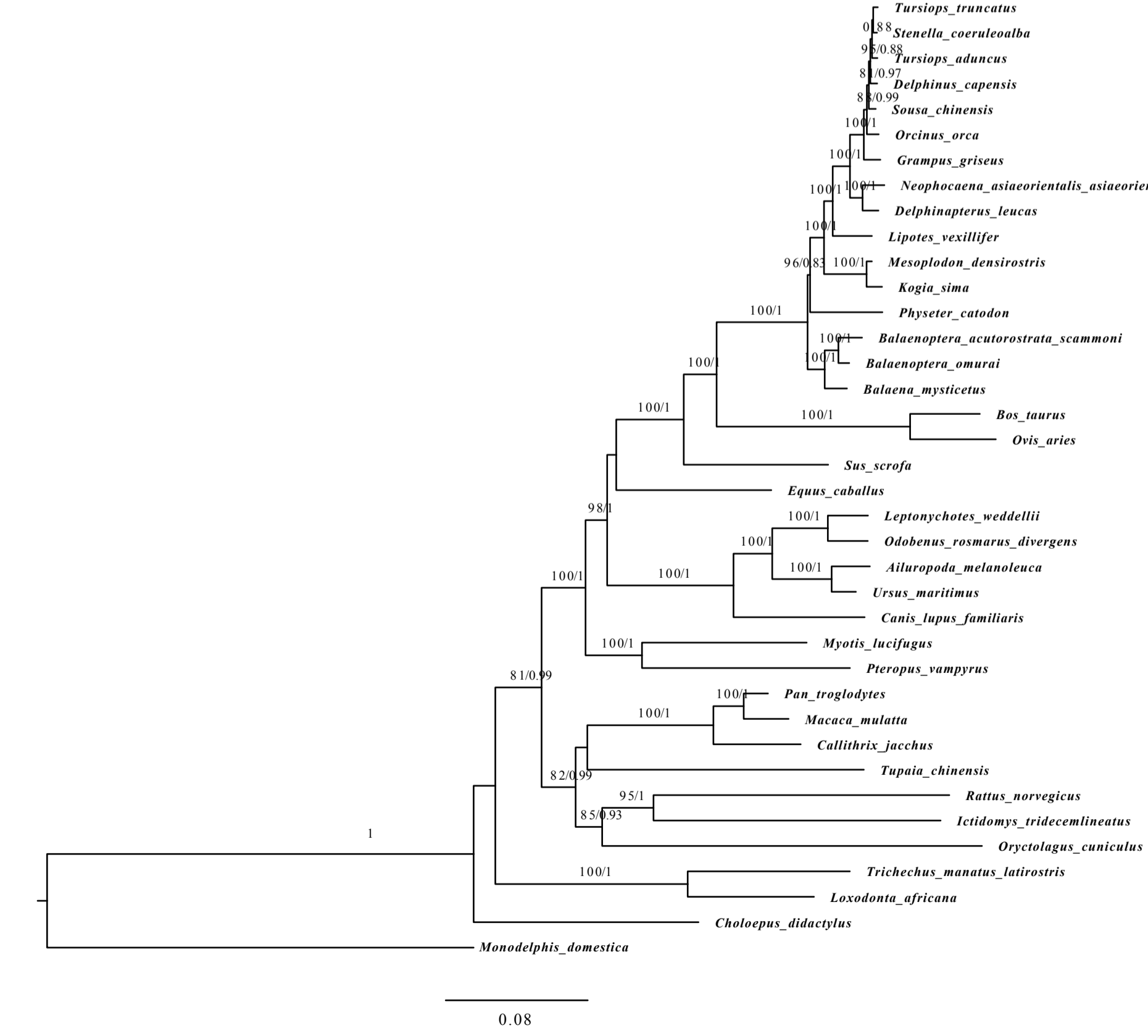

PER3

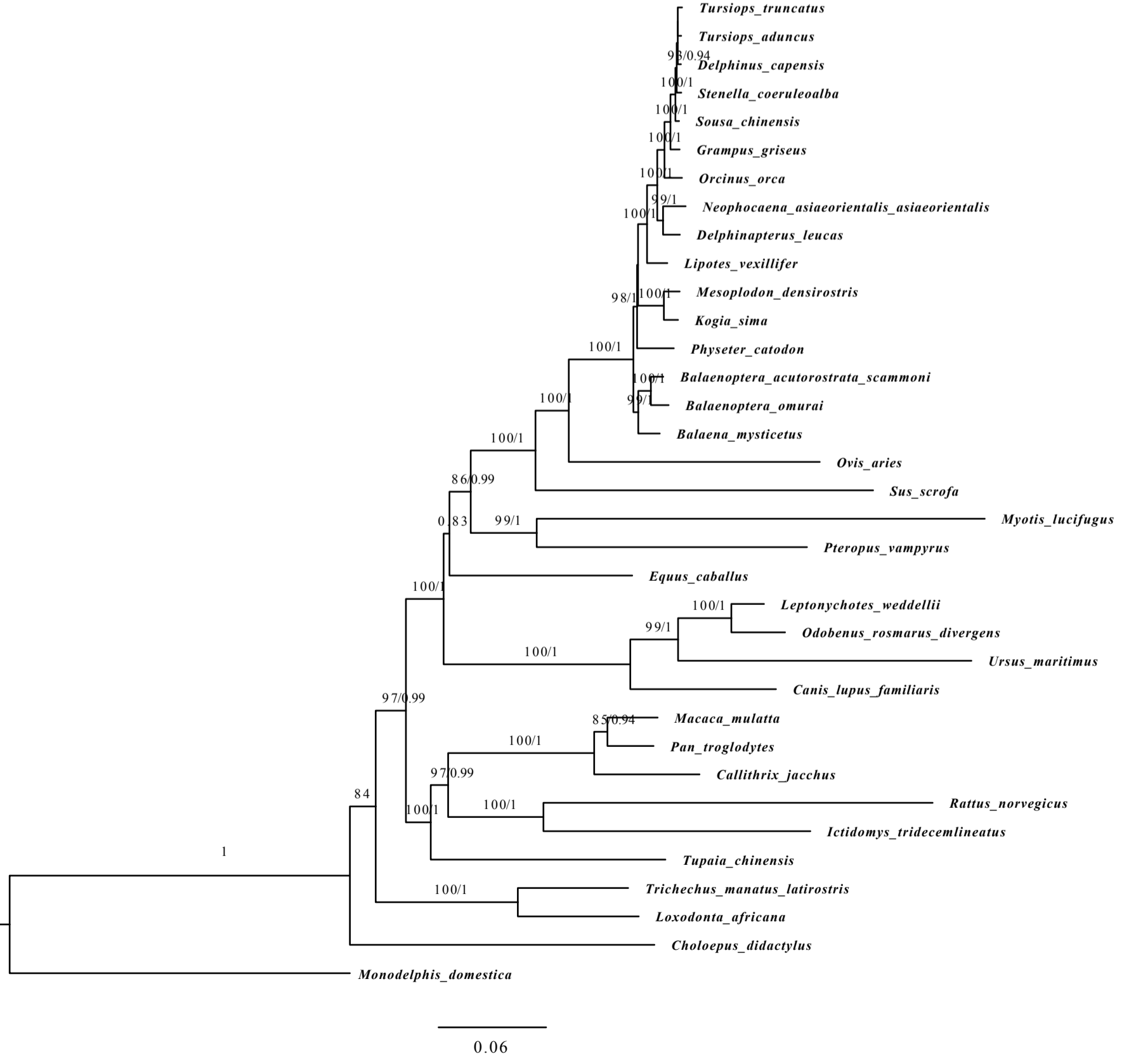

Supplement: S14 Fig — Numbers above the branches represent the ML bootstrap values and the Bayesian posterior probabilities. (PDF) [file pgen.1011598.s014.pdf]

***BMAL1***

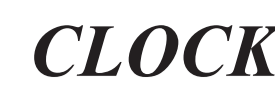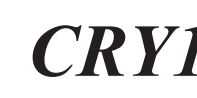

***CRY2***

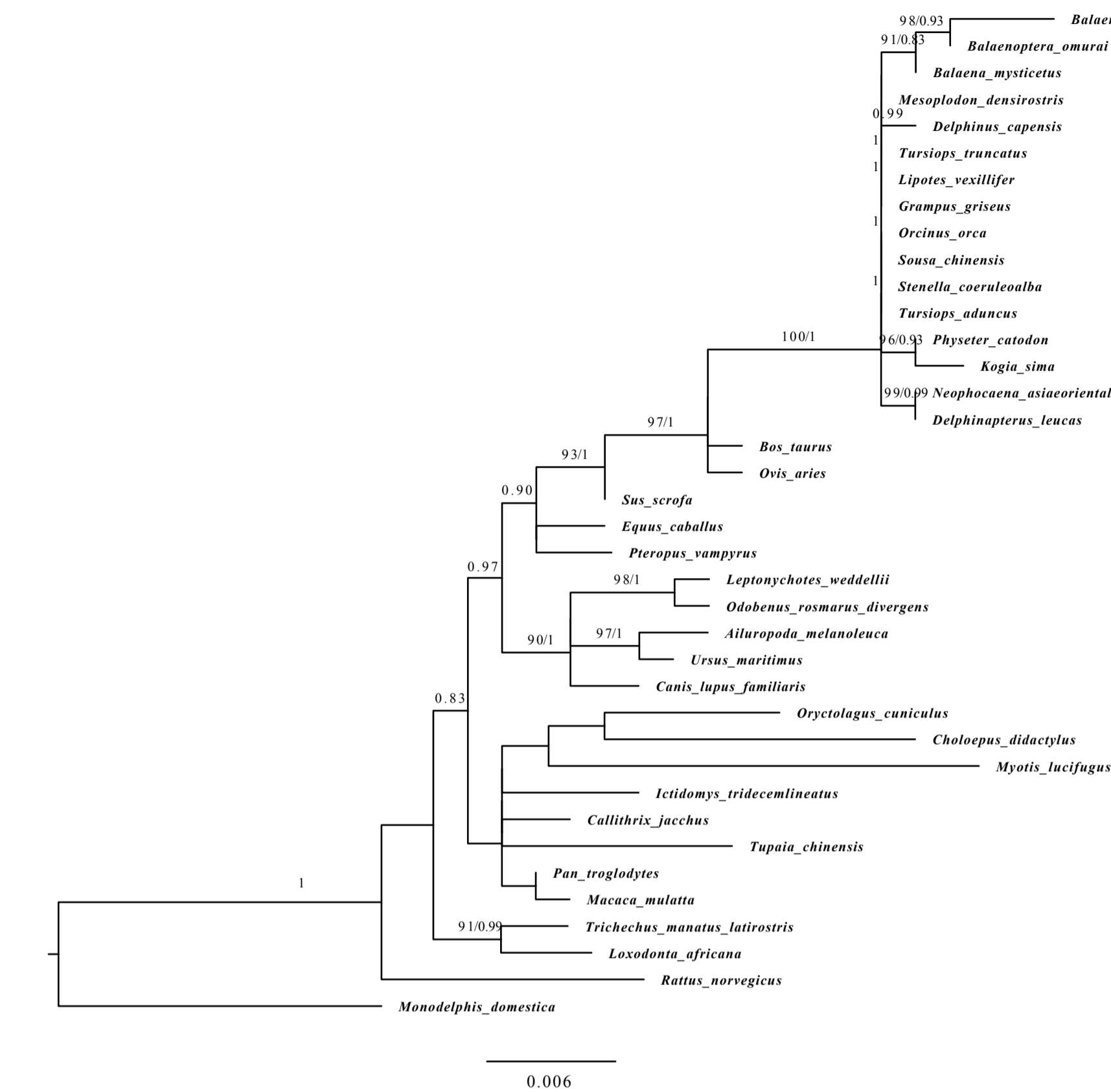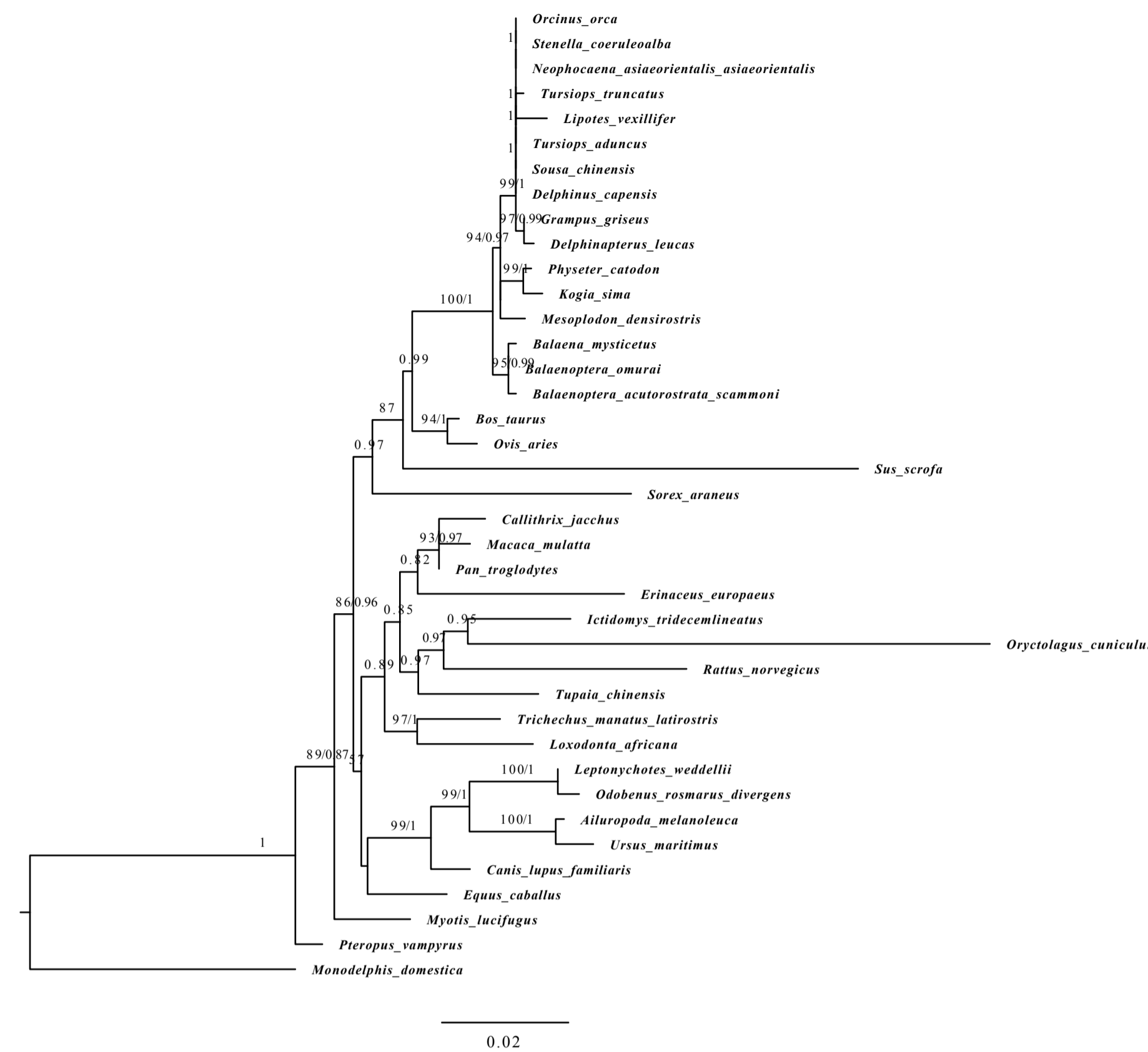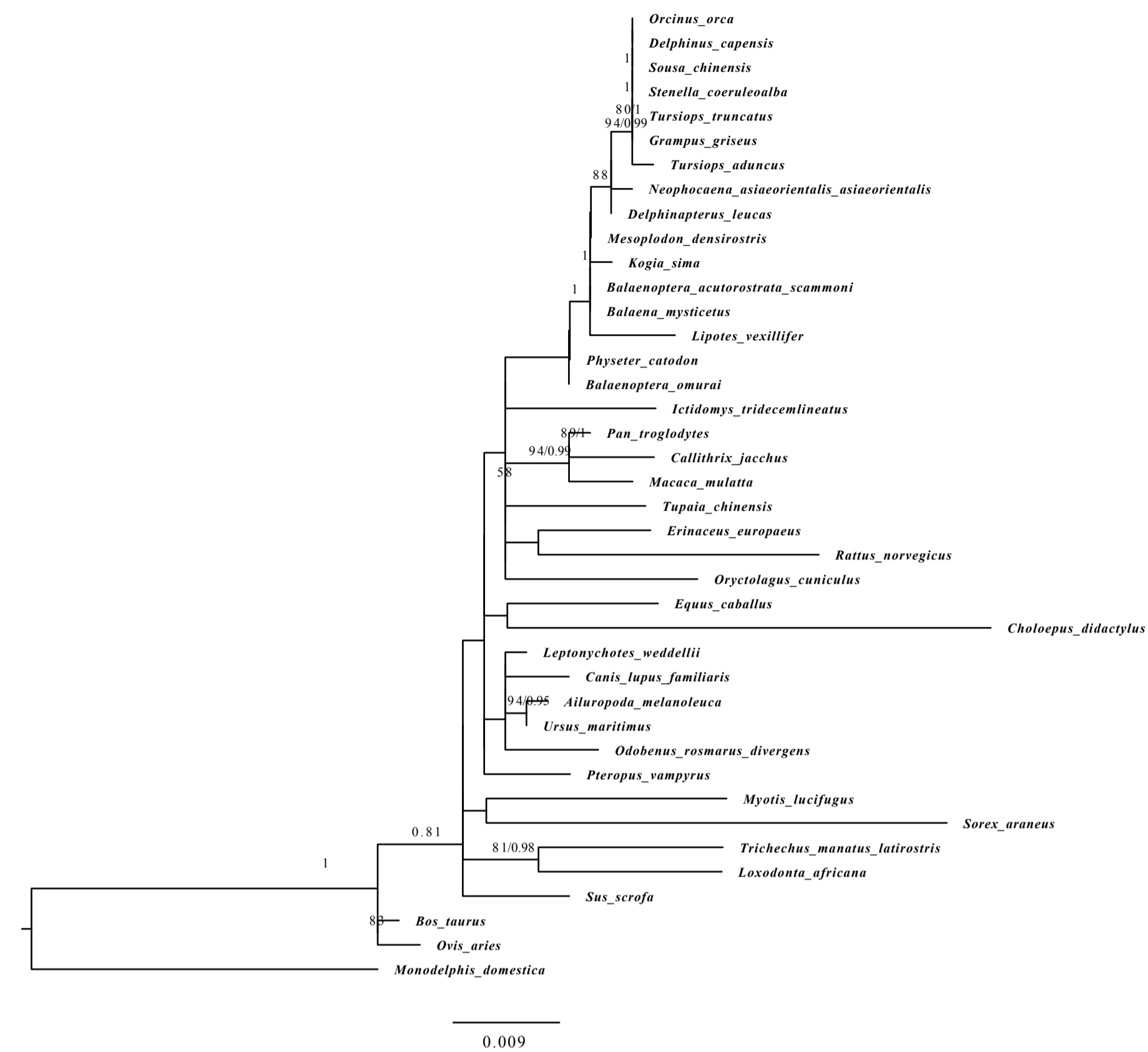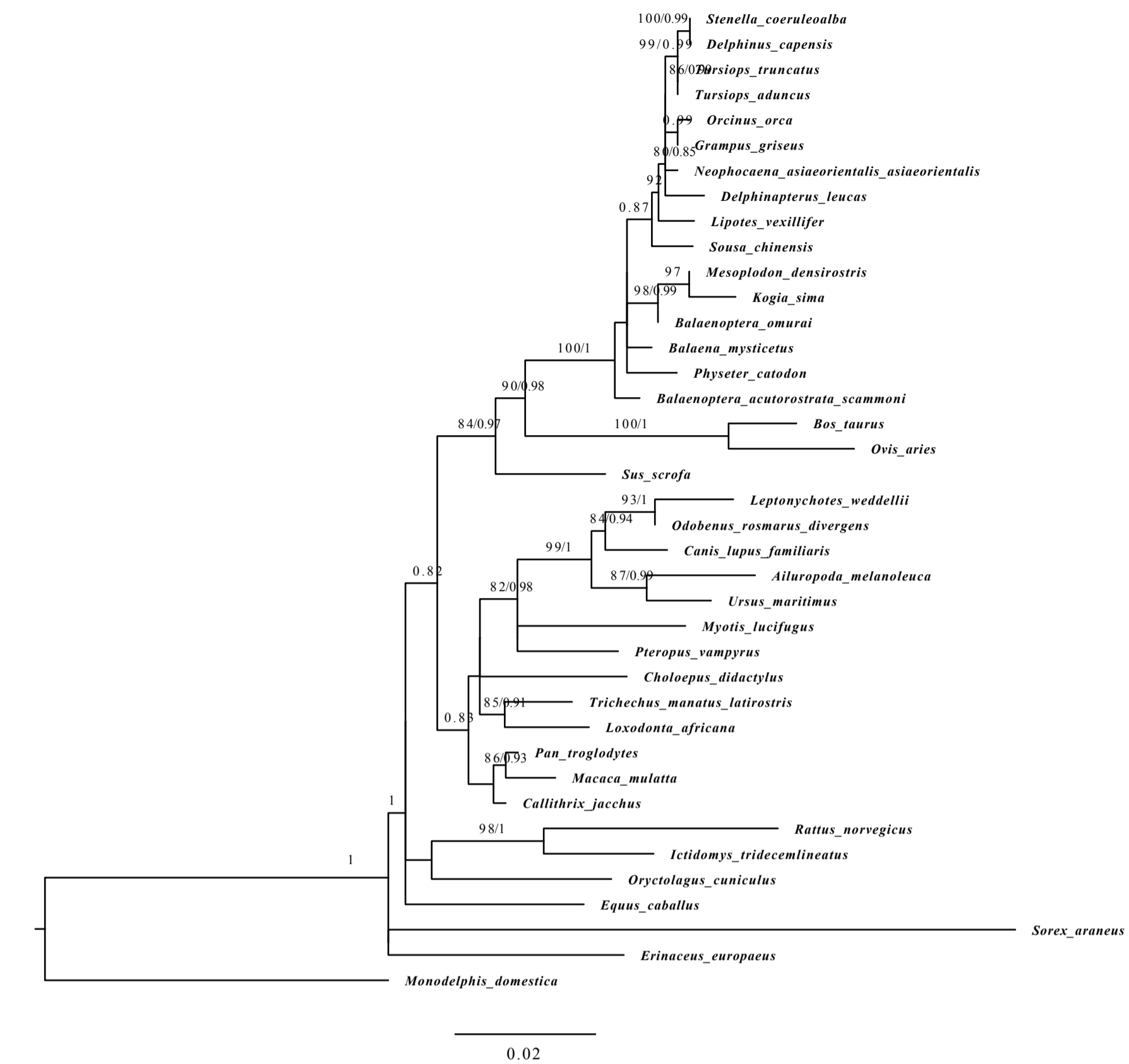

*NPAS2*

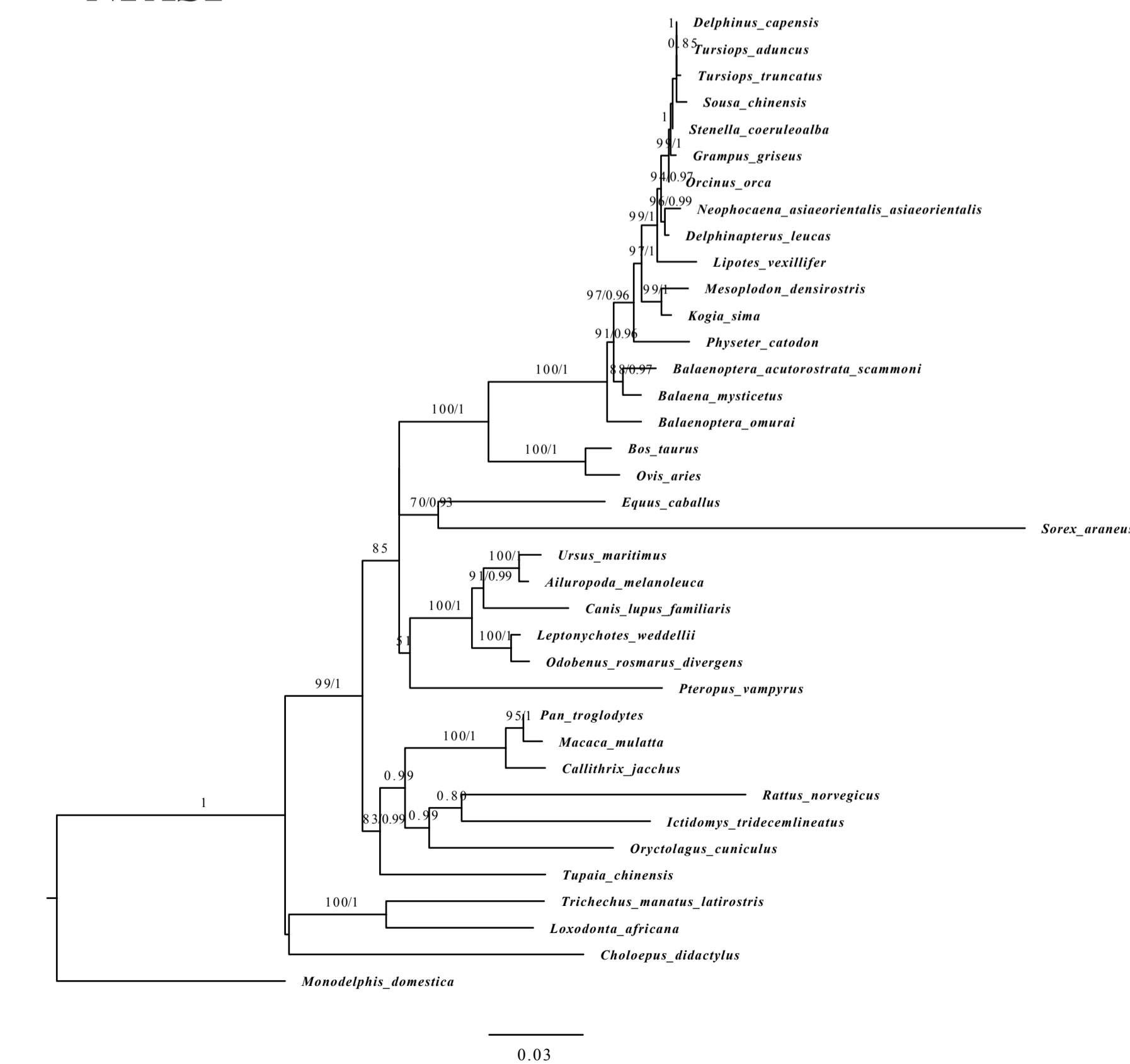

*PER*

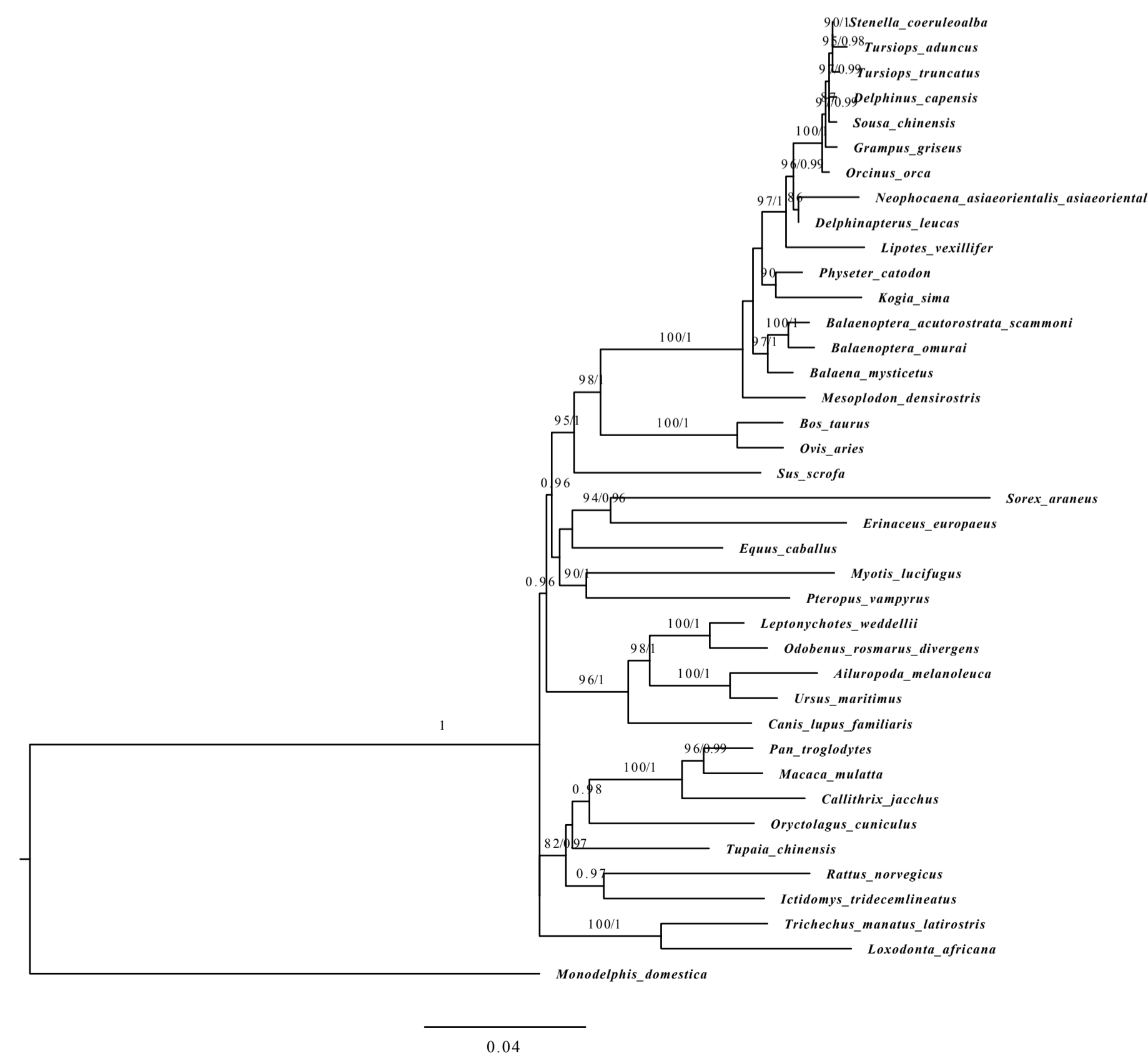

**PER2**

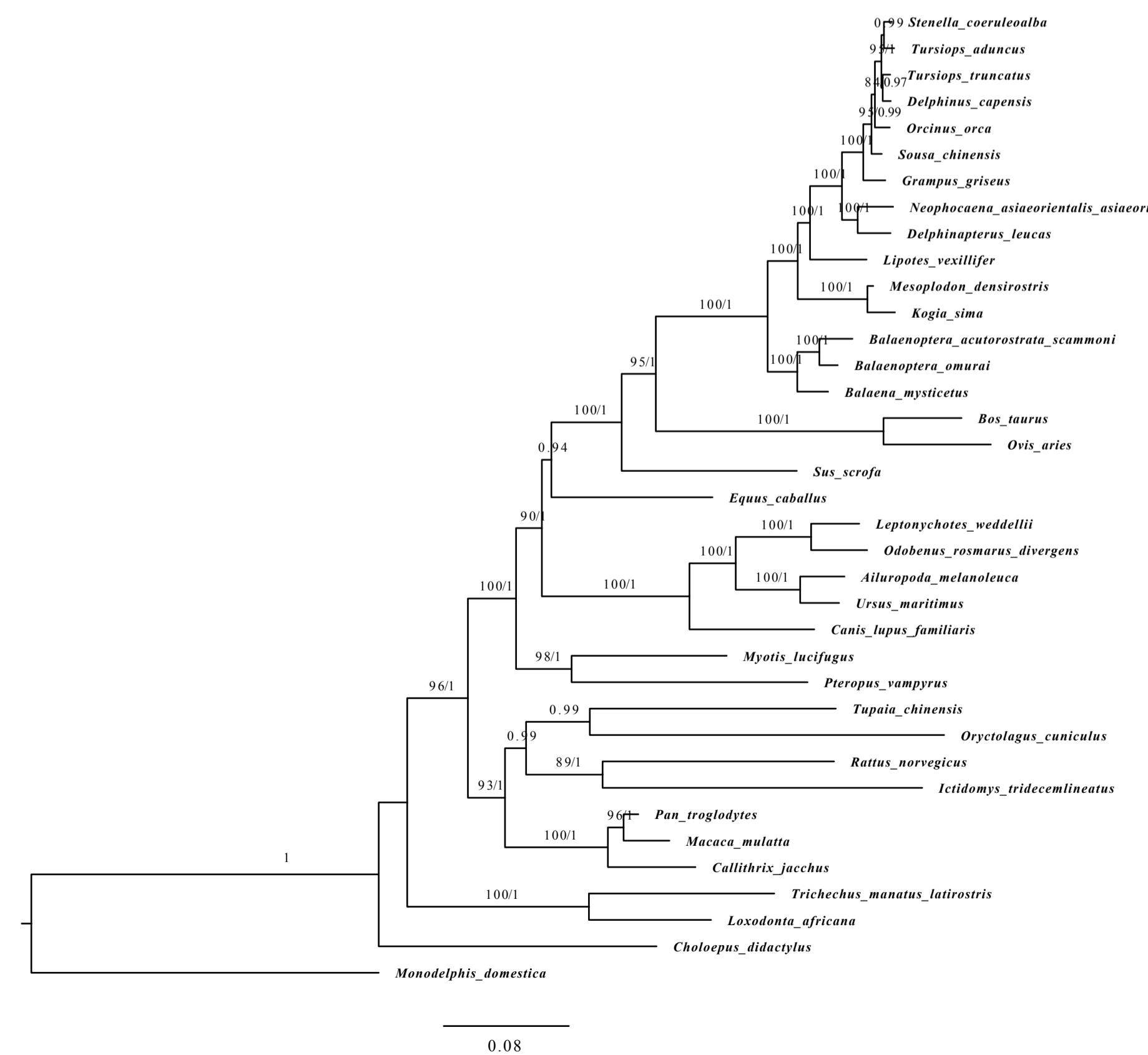

*PER3*

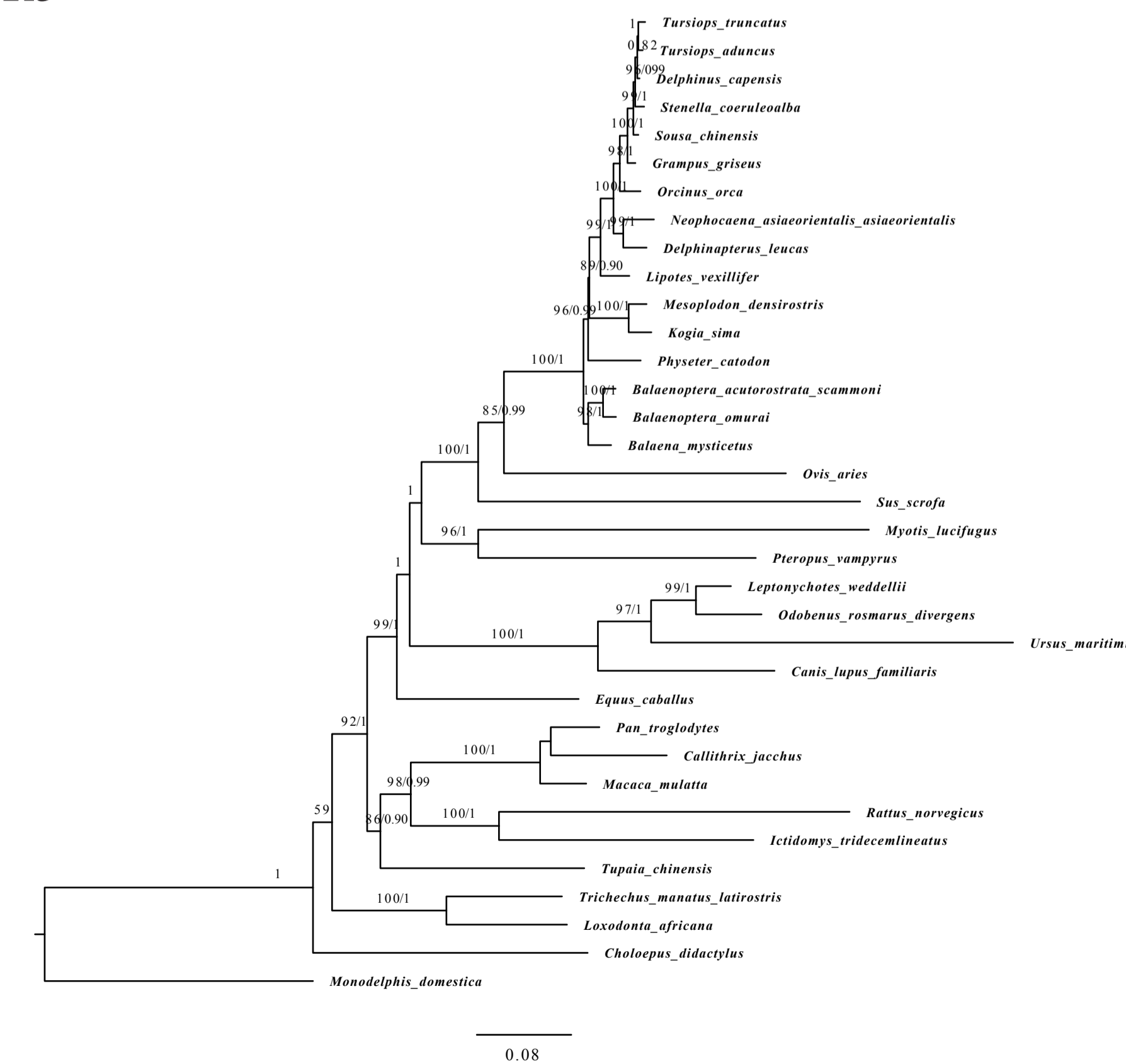

Supplement: S15 Fig — Numbers above the branches represent the ML bootstrap values and the Bayesian posterior probabilities. (PDF) [file pgen.1011598.s015.pdf]

**A**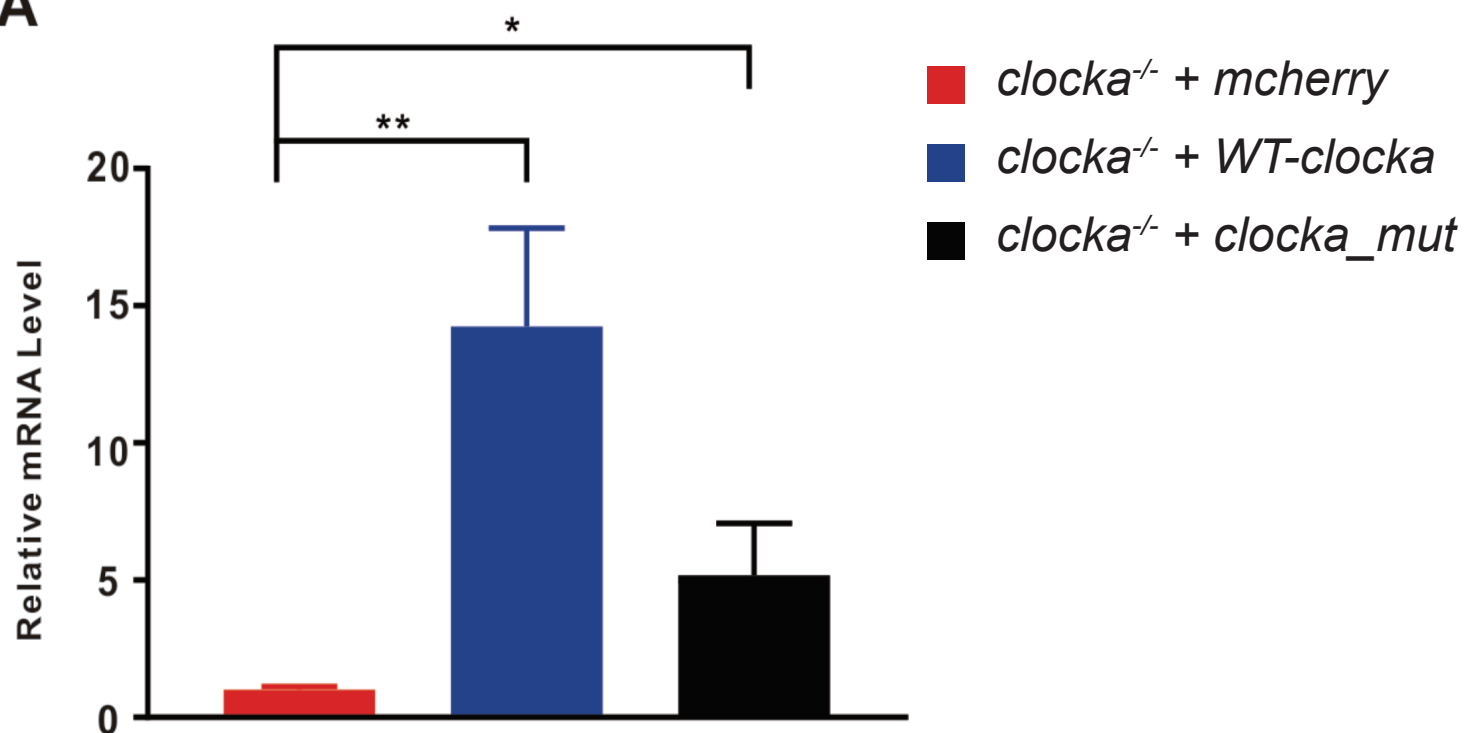**B**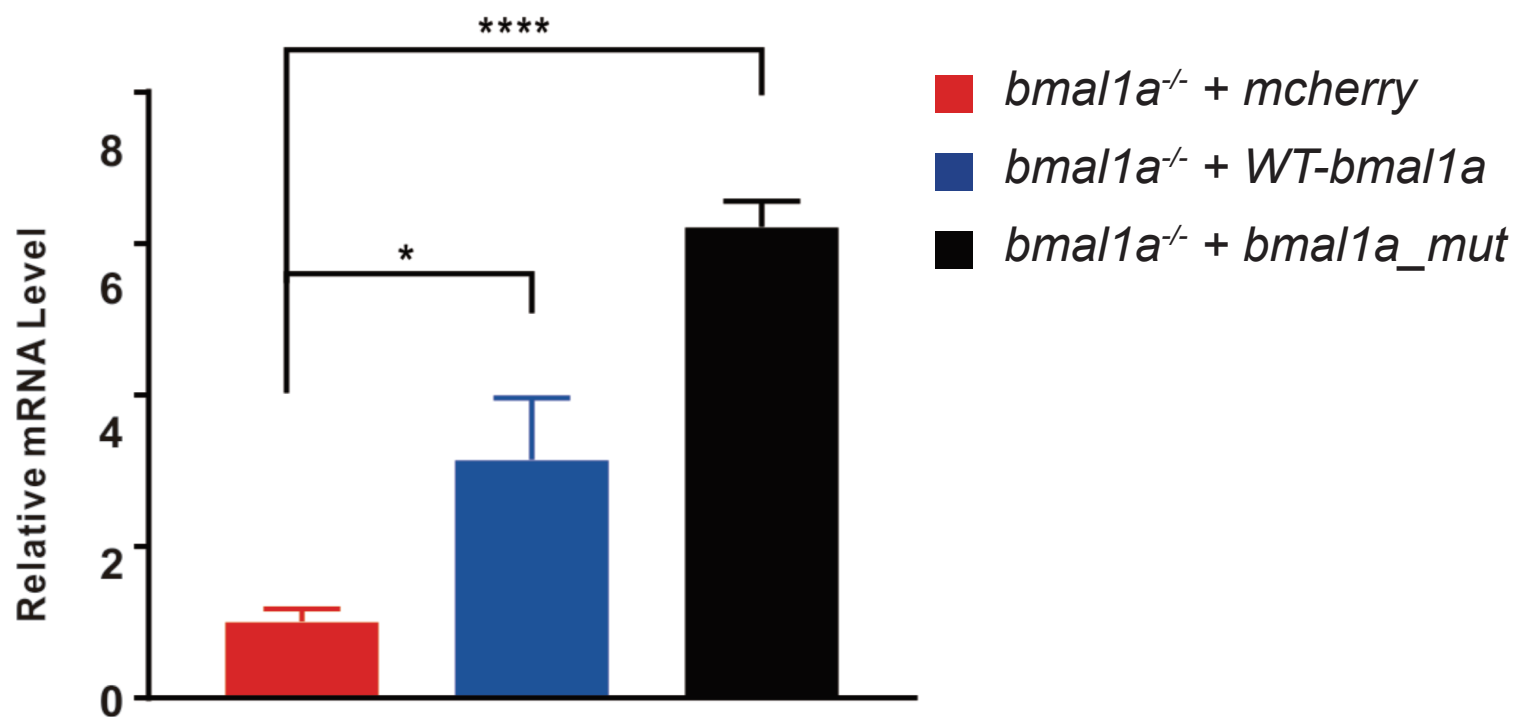

Supplement: S16 Fig — qRT-PCR analysis of clocka (A) and bmal1a (B) in 72hpf zebrafish after transient overexpression. Statistical analysis was performed using student’s t-test. *p < 0.05; **p < 0.01; ***p < 0.0001. (PDF) [file pgen.1011598.s016.pdf]
