# Supplementary material for: Evolution of canonical circadian clock genes underlies unique sleep strategies of marine mammals for secondary aquatic adaptation
Source: PLoS Genet. 2025 Mar 18;21(3):e1011598. doi: 10.1371/journal.pgen.1011598 (PMC11919277; doi:10.1371/journal.pgen.1011598)

WT-Clocka - WT-Bmal1a

Binging energy (Kcal/mol)	-25.6
Interface area (Å2)	4313.3
Numbers of Hydrogen bonds	13
Numbers of salt bridges	2

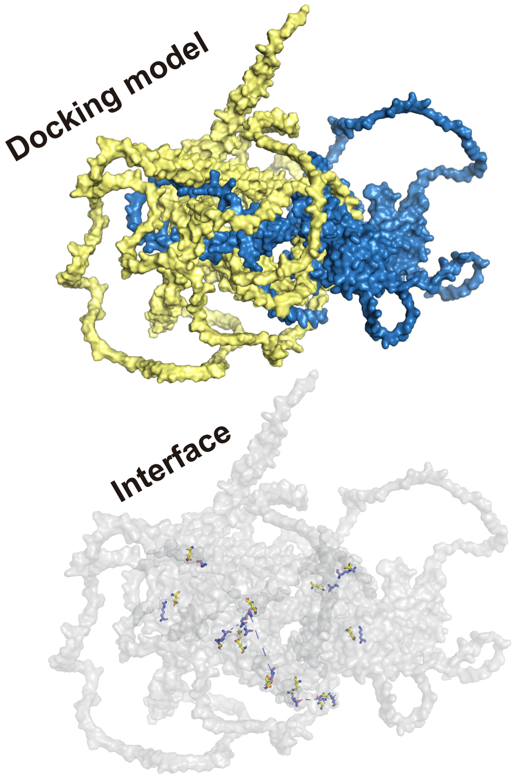

Interface Zoom

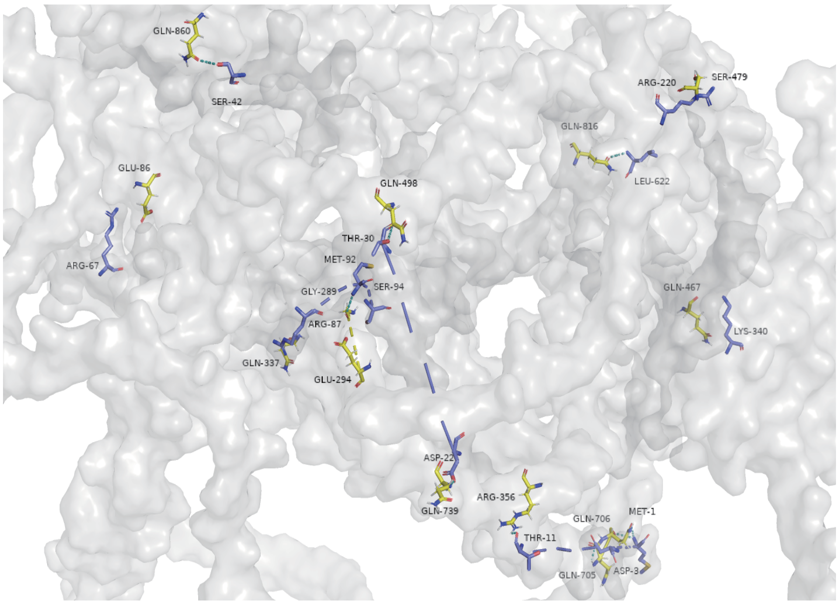

Clocka-mut - Bmal1a-mut

Binging energy (Kcal/mol)	-23.8
Interface area (Å2)	4712.8
Numbers of Hydrogen bonds	18
Numbers of salt bridges	10

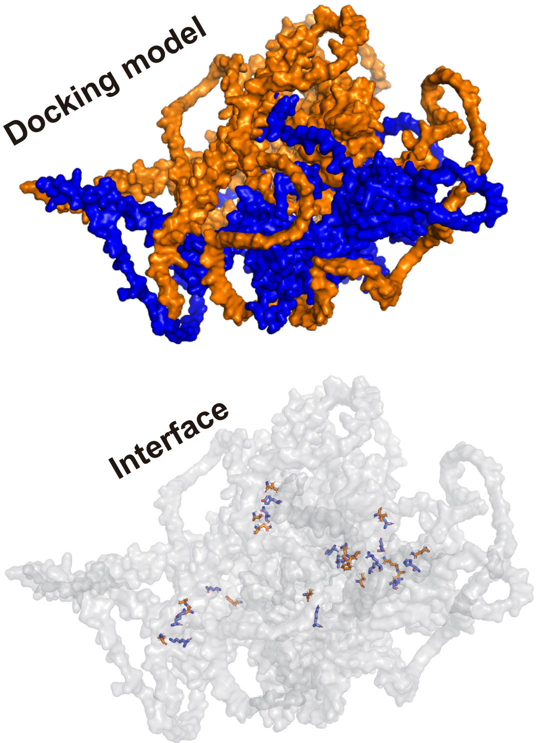

Interface Zoom

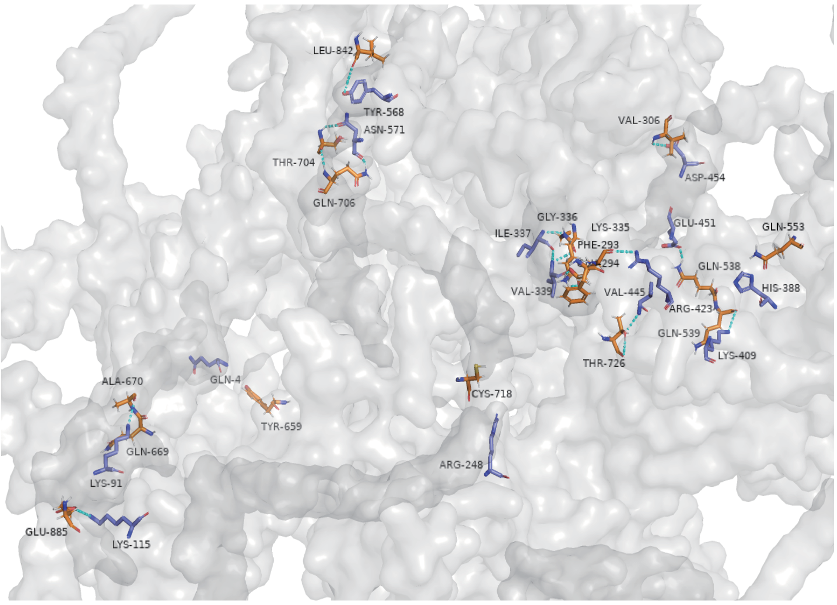

Supplement: S4 Fig — WT-Clocka, yellow; WT-Bmal1a, bule; Clocka-mut, orange; WT-Bmal1a, steel bule; hydrogen bond interaction, dotted line. (PDF) [file pgen.1011598.s004.pdf]
