# Supplementary material for: Evolution of canonical circadian clock genes underlies unique sleep strategies of marine mammals for secondary aquatic adaptation
Source: PLoS Genet. 2025 Mar 18;21(3):e1011598. doi: 10.1371/journal.pgen.1011598 (PMC11919277; doi:10.1371/journal.pgen.1011598)
Supplement: S7 Fig — (PDF) [file pgen.1011598.s007.pdf]

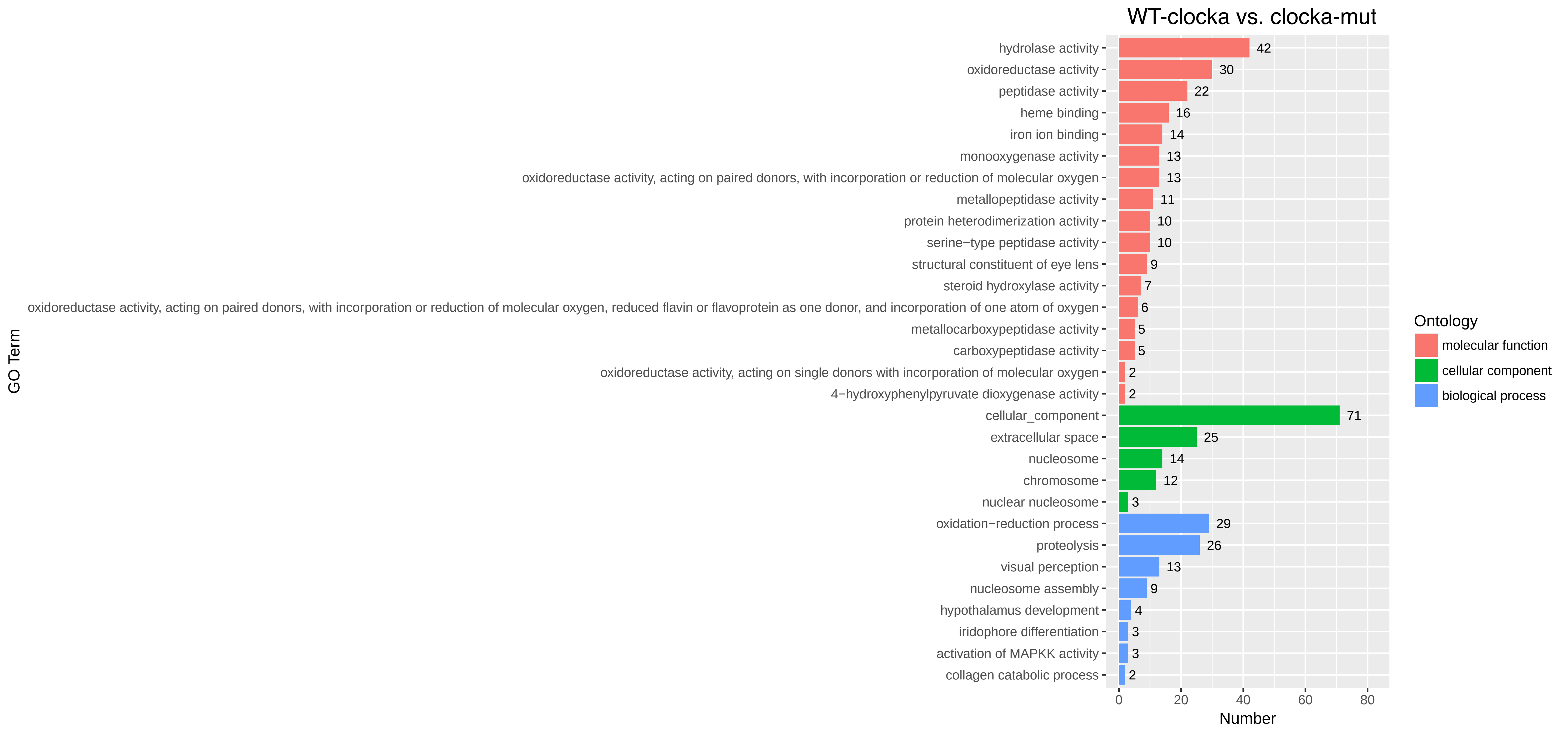

WT-clocka vs. clocka-mut
GO Term
Number
hydrolase activity 42
oxidoreductase activity 30
peptidase activity 22
heme binding 16
iron ion binding 14
monooxygenase activity 13
oxidoreductase activity, acting on paired donors, with incorporation or reduction of molecular oxygen 13
metallopeptidase activity 11
protein heterodimerization activity 10
serine−type peptidase activity 10
structural constituent of eye lens 9
steroid hydroxylase activity 7
oxidoreductase activity, acting on paired donors, with incorporation or reduction of molecular oxygen, reduced flavin or flavoprotein as one donor, and incorporation of one atom of oxygen 6
metallocarboxypeptidase activity 5
carboxypeptidase activity 5
oxidoreductase activity, acting on single donors with incorporation of molecular oxygen 2
4−hydroxyphenylpyruvate dioxygenase activity 2
cellular_component 71
extracellular space 25
nucleosome 14
chromosome 12
nuclear nucleosome 3
oxidation−reduction process 29
proteolysis 26
visual perception 13
nucleosome assembly 9
hypothalamus development 4
iridophore differentiation 3
activation of MAPKK activity 3
collagen catabolic process 2
0
20
40
60
80
Ontology
molecular function
cellular component
biological process
