# Supplementary material for: Evolution of canonical circadian clock genes underlies unique sleep strategies of marine mammals for secondary aquatic adaptation
Source: PLoS Genet. 2025 Mar 18;21(3):e1011598. doi: 10.1371/journal.pgen.1011598 (PMC11919277; doi:10.1371/journal.pgen.1011598)
Supplement: S1 Table — (DOCX) [file pgen.1011598.s017.docx]

Table S1 Statistics for amplified exons of eight circadian clock genes for nine cetaceans.

Note: tick represents successfully amplified, while cross in any genes represents no amplification despite numerous attempts.

| Species | ***BMAL1*** | | | | | | | | | | | | | | | |
| --- | --- | --- | --- | --- | --- | --- | --- | --- | --- | --- | --- | --- | --- | --- | --- | --- |
|  | Exon  1 | Exon  2 | Exon  3 | Exon  4 | Exon  5 | Exon  6 | Exon  7 | Exon  8 | Exon  9 | Exon  10 | Exon  11 | Exon  12 | Exon  13 | Exon  14 | Exon  15 | Exon  16 |
| *Stenella coeruleoalba* | √ | √ | √ | √ | √ | √ | √ | √ | √ | √ | √ | √ | √ | √ | √ | √ |
| *Delphinus capensis* | √ | √ | √ | √ | √ | √ | √ | √ | √ | √ | √ | √ | √ | √ | √ | √ |
| *Tursiops aduncus* | √ | √ | √ | √ | √ | √ | √ | √ | √ | √ | √ | √ | √ | √ | √ | √ |
| *Sousa chinensis* | √ | √ | √ | √ | √ | √ | √ | √ | √ | √ | √ | √ | √ | √ | √ | √ |
| *Grampus griseus* | √ | √ | √ | √ | √ | √ | √ | √ | √ | √ | √ | √ | √ | √ | √ | √ |
| *Delphinapterus leucas* | √ | √ | √ | √ | √ | √ | √ | X | √ | X | √ | √ | √ | √ | √ | √ |
| *Mesoplodon densirostris* | √ | √ | √ | √ | √ | √ | √ | √ | √ | √ | X | X | √ | √ | √ | √ |
| *Kogia sima* | √ | √ | √ | √ | √ | √ | √ | √ | X | √ | X | √ | √ | √ | X | √ |
| *Balaenoptera omurai* | √ | √ | √ | √ | √ | √ | √ | √ | √ | √ | √ | √ | √ | √ | X | √ |

| Species | ***CLOCK*** | | | | | | | | | | | | | | | | | | | |
| --- | --- | --- | --- | --- | --- | --- | --- | --- | --- | --- | --- | --- | --- | --- | --- | --- | --- | --- | --- | --- |
|  | Exon  1 | Exon  2 | Exon  3 | Exon  4 | Exon  5 | Exon  6 | Exon  7 | Exon  8 | Exon  9 | Exon  10 | Exon  11 | Exon  12 | Exon  13 | Exon  14 | Exon  15 | Exon  16 | Exon  17 | Exon  18 | Exon  19 | Exon  20 |
| *Stenella coeruleoalba* | √ | √ | √ | √ | √ | √ | √ | √ | √ | √ | √ | √ | √ | √ | √ | √ | √ | √ | √ | √ |
| *Delphinus capensis* | √ | √ | √ | √ | √ | √ | √ | √ | √ | √ | √ | √ | √ | √ | √ | √ | X | √ | √ | √ |
| *Tursiops aduncus* | √ | √ | √ | √ | √ | √ | √ | √ | √ | √ | √ | √ | √ | √ | X | √ | √ | √ | √ | √ |
| *Sousa chinensis* | X | √ | √ | √ | √ | √ | √ | √ | √ | √ | √ | √ | √ | √ | √ | √ | √ | √ | √ | √ |
| *Grampus griseus* | √ | √ | √ | √ | √ | √ | X | √ | √ | √ | √ | √ | √ | √ | √ | √ | √ | √ | √ | √ |
| *Delphinapterus leucas* | √ | √ | √ | √ | √ | √ | √ | √ | √ | √ | √ | √ | √ | √ | √ | √ | √ | √ | √ | √ |
| *Mesoplodon densirostris* | √ | √ | √ | √ | X | X | √ | √ | √ | √ | √ | √ | √ | √ | √ | √ | √ | √ | √ | √ |
| *Kogia sima* | √ | √ | √ | √ | √ | √ | X | X | √ | √ | √ | √ | √ | √ | √ | √ | √ | √ | √ | X |
| *Balaenoptera omurai* | √ | √ | √ | √ | √ | √ | √ | √ | √ | √ | √ | √ | √ | √ | √ | √ | √ | √ | √ | √ |

| Species | ***NPAS2*** | | | | | | | | | | | | | | | | | | | |
| --- | --- | --- | --- | --- | --- | --- | --- | --- | --- | --- | --- | --- | --- | --- | --- | --- | --- | --- | --- | --- |
|  | Exon  1 | Exon  2 | Exon  3 | Exon  4 | Exon  5 | Exon  6 | Exon  7 | Exon  8 | Exon  9 | Exon  10 | Exon  11 | Exon  12 | Exon  13 | Exon  14 | Exon  15 | Exon  16 | Exon  17 | Exon  18 | Exon  19 | Exon  20 |
| *Stenella coeruleoalba* | X | √ | √ | √ | √ | √ | √ | √ | √ | √ | √ | √ | √ | √ | √ | √ | X | √ | √ | √ |
| *Delphinus capensis* | X | √ | √ | √ | √ | √ | √ | √ | √ | √ | √ | √ | √ | √ | √ | √ | √ | √ | √ | √ |
| *Tursiops aduncus* | X | √ | √ | √ | √ | √ | √ | √ | X | √ | √ | √ | √ | √ | √ | √ | √ | √ | √ | √ |
| *Sousa chinensis* | X | √ | √ | √ | √ | √ | √ | √ | √ | √ | √ | √ | √ | √ | √ | √ | √ | √ | √ | √ |
| *Grampus griseus* | X | √ | X | √ | √ | √ | √ | √ | X | √ | √ | √ | √ | √ | √ | √ | √ | √ | √ | √ |
| *Delphinapterus leucas* | X | √ | √ | √ | √ | √ | √ | √ | √ | √ | √ | √ | √ | √ | √ | √ | √ | √ | √ | √ |
| *Mesoplodon densirostris* | X | √ | √ | √ | √ | √ | X | √ | X | √ | √ | √ | X | √ | √ | √ | √ | √ | √ | √ |
| *Kogia sima* | X | √ | √ | √ | √ | √ | √ | √ | X | √ | √ | √ | X | √ | √ | √ | √ | √ | √ | √ |
| *Balaenoptera omurai* | X | √ | √ | √ | √ | √ | √ | √ | √ | √ | √ | X | √ | √ | √ | √ | √ | √ | √ | √ |

| Species | ***CRY1*** | | | | | | | | | | | | |
| --- | --- | --- | --- | --- | --- | --- | --- | --- | --- | --- | --- | --- | --- |
|  | Exon  1 | Exon  2 | Exon  3 | Exon  4 | Exon  5 | Exon  6 | Exon  7 | Exon  8 | Exon  9 | Exon  10 | Exon  11 | Exon  12 |  |
| *Stenella coeruleoalba* | √ | √ | √ | √ | √ | √ | √ | √ | √ | √ | √ | √ |  |
| *Delphinus capensis* | √ | √ | √ | √ | √ | √ | √ | √ | √ | √ | √ | √ |  |
| *Tursiops aduncus* | √ | √ | √ | √ | √ | √ | √ | √ | √ | √ | √ | √ |  |
| *Sousa chinensis* | √ | √ | √ | √ | √ | √ | √ | √ | √ | √ | √ | √ |  |
| *Grampus griseus* | √ | √ | √ | √ | √ | √ | X | √ | √ | √ | √ | √ |  |
| *Delphinapterus leucas* | √ | √ | √ | √ | √ | √ | √ | √ | √ | √ | √ | √ |  |
| *Mesoplodon densirostris* | √ | √ | √ | √ | √ | √ | √ | √ | √ | √ | √ | √ |  |
| *Kogia sima* | √ | √ | √ | √ | √ | √ | √ | X | √ | √ | √ | √ |  |
| *Balaenoptera omurai* | √ | √ | √ | √ | X | √ | √ | √ | √ | √ | √ | √ |  |

| Species | ***CRY2*** | | | | | | | | | | | |
| --- | --- | --- | --- | --- | --- | --- | --- | --- | --- | --- | --- | --- |
|  | Exon  1 | Exon  2 | Exon  3 | Exon  4 | Exon  5 | Exon  6 | Exon  7 | Exon  8 | Exon  9 | Exon  10 | Exon  11 |  |
| *Stenella coeruleoalba* | √ | √ | √ | √ | √ | √ | √ | √ | √ | √ | √ |  |
| *Delphinus capensis* | √ | √ | √ | √ | √ | √ | X | √ | √ | √ | √ |  |
| *Tursiops aduncus* | √ | √ | √ | √ | √ | √ | √ | √ | √ | √ | √ |  |
| *Sousa chinensis* | √ | √ | √ | √ | √ | √ | X | √ | √ | √ | √ |  |
| *Grampus griseus* | √ | √ | √ | √ | √ | √ | √ | √ | √ | √ | √ |  |
| *Delphinapterus leucas* | √ | √ | √ | √ | √ | √ | √ | √ | √ | √ | √ |  |
| *Mesoplodon densirostris* | √ | √ | √ | √ | √ | √ | √ | √ | √ | √ | √ |  |
| *Kogia sima* | √ | √ | √ | √ | √ | √ | X | X | X | √ | √ |  |
| *Balaenoptera omurai* | √ | √ | √ | √ | √ | √ | √ | √ | √ | √ | X |  |

| Species | ***PER1*** | | | | | | | | | | | | | | | | | | | | | |
| --- | --- | --- | --- | --- | --- | --- | --- | --- | --- | --- | --- | --- | --- | --- | --- | --- | --- | --- | --- | --- | --- | --- |
|  | Exon  1 | Exon  2 | Exon  3 | Exon  4 | Exon  5 | Exon  6 | Exon  7 | Exon  8 | Exon  9 | Exon  10 | Exon  11 | Exon  12 | Exon  13 | Exon  14 | Exon  15 | Exon  16 | Exon  17 | Exon  18 | Exon  19 | Exon  20 | Exon  21 | Exon  22 |
| *Stenella coeruleoalba* | √ | √ | √ | √ | √ | X | √ | √ | √ | √ | √ | √ | X | √ | X | √ | √ | √ | √ | √ | √ | √ |
| *Delphinus capensis* | √ | √ | √ | √ | √ | √ | √ | √ | √ | √ | √ | √ | X | √ | X | √ | √ | √ | √ | √ | √ | √ |
| *Tursiops aduncus* | √ | √ | √ | √ | √ | √ | √ | √ | √ | √ | √ | √ | X | √ | √ | √ | √ | √ | √ | √ | √ | √ |
| *Sousa chinensis* | √ | √ | √ | √ | √ | √ | √ | √ | √ | √ | √ | √ | X | √ | X | √ | √ | √ | √ | √ | √ | √ |
| *Grampus griseus* | √ | √ | √ | √ | √ | X | √ | √ | √ | √ | √ | √ | X | √ | X | √ | √ | √ | √ | √ | √ | √ |
| *Delphinapterus leucas* | X | √ | √ | √ | √ | √ | X | X | √ | √ | √ | √ | √ | √ | √ | √ | X | √ | √ | √ | √ | √ |
| *Mesoplodon densirostris* | √ | √ | √ | √ | X | √ | √ | √ | √ | √ | √ | X | √ | √ | √ | X | √ | X | √ | √ | √ | X |
| *Kogia sima* | √ | √ | √ | √ | √ | √ | X | X | √ | √ | √ | √ | X | √ | X | √ | X | √ | X | √ | √ | √ |
| *Balaenoptera omurai* | √ | √ | √ | √ | √ | X | √ | √ | X | √ | √ | √ | X | √ | √ | X | √ | X | √ | √ | √ | √ |

| Species | ***PER2*** | | | | | | | | | | | | | | | | | | | | | |
| --- | --- | --- | --- | --- | --- | --- | --- | --- | --- | --- | --- | --- | --- | --- | --- | --- | --- | --- | --- | --- | --- | --- |
|  | Exon  1 | Exon  2 | Exon  3 | Exon  4 | Exon  5 | Exon  6 | Exon  7 | Exon  8 | Exon  9 | Exon  10 | Exon  11 | Exon  12 | Exon  13 | Exon  14 | Exon  15 | Exon  16 | Exon  17 | Exon  18 | Exon  19 | Exon  20 | Exon  21 | Exon  22 |
| *Stenella coeruleoalba* | √ | √ | √ | √ | √ | √ | √ | √ | √ | X | X | √ | √ | √ | √ | √ | √ | √ | √ | √ | √ | √ |
| *Delphinus capensis* | √ | √ | √ | √ | √ | √ | √ | √ | √ | √ | √ | √ | √ | √ | √ | √ | √ | √ | √ | X | √ | √ |
| *Tursiops aduncus* | √ | √ | √ | √ | √ | X | √ | √ | √ | √ | √ | √ | √ | √ | √ | √ | √ | √ | √ | √ | √ | √ |
| *Sousa chinensis* | √ | √ | √ | √ | √ | √ | √ | √ | √ | √ | √ | √ | √ | √ | √ | √ | √ | √ | √ | √ | √ | √ |
| *Grampus griseus* | √ | √ | √ | √ | √ | √ | √ | √ | √ | X | √ | √ | √ | X | √ | √ | √ | √ | √ | √ | √ | √ |
| *Delphinapterus leucas* | √ | √ | √ | √ | √ | √ | √ | √ | √ | √ | √ | √ | √ | √ | √ | √ | √ | √ | √ | X | X | X |
| *Mesoplodon densirostris* | √ | √ | √ | √ | X | √ | √ | √ | √ | √ | √ | X | X | √ | √ | √ | √ | X | √ | √ | √ | X |
| *Kogia sima* | √ | √ | √ | √ | √ | √ | √ | X | X | X | X | √ | √ | X | √ | √ | √ | √ | √ | √ | √ | √ |
| *Balaenoptera omurai* | √ | √ | X | X | √ | √ | √ | X | √ | √ | √ | √ | √ | √ | √ | √ | √ | √ | √ | √ | X | √ |

| Species | ***PER3*** | | | | | | | | | | | | | | | | | | | | | |
| --- | --- | --- | --- | --- | --- | --- | --- | --- | --- | --- | --- | --- | --- | --- | --- | --- | --- | --- | --- | --- | --- | --- |
|  | Exon  1 | Exon  2 | Exon  3 | Exon  4 | Exon  5 | Exon  6 | Exon  7 | Exon  8 | Exon  9 | Exon  10 | Exon  11 | Exon  12 | Exon  13 | Exon  14 | Exon  15 | Exon  16 | Exon  17 | Exon  18 | Exon  19 | Exon  20 | Exon  21 |  |
| *Stenella coeruleoalba* | √ | √ | √ | √ | √ | √ | √ | √ | √ | √ | √ | √ | √ | √ | √ | X | √ | √ | √ | X | √ |  |
| *Delphinus capensis* | √ | √ | √ | √ | √ | √ | √ | √ | √ | √ | √ | √ | √ | √ | √ | √ | √ | √ | √ | X | √ |  |
| *Tursiops aduncus* | √ | √ | √ | √ | √ | √ | √ | √ | √ | √ | √ | √ | √ | √ | √ | √ | √ | √ | √ | X | √ |  |
| *Sousa chinensis* | √ | √ | √ | √ | √ | √ | √ | √ | √ | √ | √ | √ | √ | √ | √ | √ | √ | X | X | X | √ |  |
| *Grampus griseus* | √ | √ | √ | √ | √ | √ | √ | √ | √ | √ | √ | √ | √ | √ | √ | √ | √ | √ | √ | X | √ |  |
| *Delphinapterus leucas* | √ | √ | √ | √ | √ | √ | √ | √ | √ | √ | √ | √ | √ | √ | √ | √ | √ | √ | X | X | X |  |
| *Mesoplodon densirostris* | √ | √ | √ | √ | √ | √ | X | √ | √ | √ | √ | √ | √ | √ | √ | √ | √ | X | √ | X | X |  |
| *Kogia sima* | √ | √ | √ | √ | √ | √ | √ | √ | √ | √ | X | X | √ | √ | X | √ | √ | X | √ | X | √ |  |
| *Balaenoptera omurai* | √ | √ | √ | √ | √ | √ | √ | √ | √ | √ | √ | √ | √ | √ | X | √ | √ | √ | √ | X | √ |  |
