# Supplementary material for: Evolution of canonical circadian clock genes underlies unique sleep strategies of marine mammals for secondary aquatic adaptation
Source: PLoS Genet. 2025 Mar 18;21(3):e1011598. doi: 10.1371/journal.pgen.1011598 (PMC11919277; doi:10.1371/journal.pgen.1011598)
Supplement: S2 Table — (DOCX) [file pgen.1011598.s018.docx]

Table S2 Sequence data used in present study, including taxonomy and accession numbers or Ensembl ID.

| **Classification** | **Species name** | **Common name** | **Accession number** | | | | | | | |
| --- | --- | --- | --- | --- | --- | --- | --- | --- | --- | --- |
|  |  |  | ***CLOCK*** | ***BMAL1*** | ***NPAS2*** | ***PER1*** | ***PER2*** | ***PER3*** | ***CRY1*** | ***CRY2*** |
| Cetartiodactyla | *Tursiops truncatus* | Common bottlenose dolphin | ENSTTRT00000000960 | ENSTTRT00000013930 | XM_033838601.1 | XM_033848465.1 | XM_004326218.1 | XM_019930926.2 | XM_033866171.1 | XM_004311837.1 |
|  | *Lipotes vexillifer* | Yangtze River dolphin | XM_007450264.1 | XM_007471417.1 | XM_007468334.1 | XM_007457808.1 | NW_006779703.1 | XM_007453306.1 | XM_007462867.1 | XM_007455797.1 |
|  | *Physeter catodon* | Sperm whale | XM_007121139.1 | XM_007116653.1 | XM_007121340.1 | XM_007126609.1 | XM_024124725.3 | XM_007106513.1 | XM_024127403.3 | XM_007124976.1 |
|  | *Balaenoptera acutorostrata* | Minke whale | XM_007168433.1 | XM_007174899.1 | XM_057558518.1 | XM_007166362.1 | XM_057551296.1 | XM_007170049.1 | XM_007165725.1 | XM_007181123.1 |
|  | *Balaena mysticetus* | Bowhead whale | bmy_09828 | bmy_04014 | scaffold_489 | scaffold_43 | scaffold_1633 | scaffold_396 | scaffold_262 | scaffold_127 |
|  | *Orcinus orca* | Killer whale | XM_004268260.2 | XM_012536501.1 | XM_012535937.1 | XM_012532111.1 | XM_004262543.1 | NW_004438465.1 | XM_004269411.1 | XM_004264040.2 |
|  | *Neophocaena asiaeorientalis* | Yangtze finless porpoise | XM_024746683.1 | XM_024756984.1 | XM_024744027.1 | XM_024748425.1 | XM_024752771.1 | XM_024749934.1 | XM_024732200.1 | XM_024748921.1 |
|  | *Stenella coeruleoalba* | Striped dolphin | Amplified | Amplified | Amplified | Amplified | Amplified | Amplified | Amplified | Amplified |
|  | *Delphinus capensis* | Long-beaked commom dolphin | Amplified | Amplified | Amplified | Amplified | Amplified | Amplified | Amplified | Amplified |
|  | *Tursiops aduncus* | Indo-pacific bottlenose dolphin | Amplified | Amplified | Amplified | Amplified | Amplified | Amplified | Amplified | Amplified |
|  | *Sousa chinensis* | Indo-pacific humpbacked dolphin | Amplified | Amplified | Amplified | Amplified | Amplified | Amplified | Amplified | Amplified |
|  | *Grampus griseus* | Risso's dolphin | Amplified | Amplified | Amplified | Amplified | Amplified | Amplified | Amplified | Amplified |
|  | *Delphinapterus leucas* | Beluga whale | Amplified  XM_022563942.2 | Amplified  XM_022555707.2 | Amplified | Amplified  XM_022595687.2 | Amplified  XM_022568118.1 | Amplified  XM_022598308.2 | Amplified | Amplified |
|  | *Mesoplodon densirostris* | Blainville's beaked whale | Amplified  XM_060109516.1 | Amplified  XM_060103338.1 | Amplified  XM_060117277.1 | Amplified  XM_060081490.1 | Amplified  XM_060106124.1 | Amplified  XM_060088905.1 | Amplified  XM_060112001.1 | Amplified  XM_060104161.1 |
|  | *Kogia sima* | Dwarf sperm whale | Amplified | Amplified | Amplified | Amplified | Amplified | Amplified | Amplified | Amplified |
|  | *Balaenoptera omurai* | Omura's whale | Amplified | Amplified | Amplified | Amplified | Amplified | Amplified | Amplified | Amplified |
|  | *Bos taurus* | Cow | ENSBTAT00000061480 | XM_010812471.2 | NM_001083763.1 | XM_015458725.1 | NM_001192317.1 | XM_059875624.1 | NM_001105415.1 | ENSBTAT00000028277 |
|  | *Ovis aries* | Sheep | ENSOART00000001733 | XM_012095273.2 | XM_004006132.3 | XM_012122516.2 | XM_012134485.2 | XM_060396123.1 | XM_012159444.2 | NM_001129736.1 |
|  | *Sus scrofa* | Pig | XM_021101318.1 | NC_010444.3 | XM_021087167.1 | ENSSSCG00000017983 | XM_021074887.1 | XM_021095271.1 | ENSSSCG00000000164 | XM_021083144.1 |
| Perissodactyla | *Equus caballus* | Horse | ENSECAG00000006331 | ENSECAT00000013236 | XM_023618546.1 | ENSECAG00000013291 | XM_023642708.1 | XM_005607555.3 | ENSECAG00000014833 | XM_023653937.1 |
| Carnivora | *Leptonychotes weddellii* | Seal | XM_006752468.2 | NW_006385075.1 | NW_006383595.1 | NW_006383983.1 | XM_031020744.1 | XM_031017959.1 | XM_006731050.1 | NW_006387418.1 |
|  | *Odobenus rosmarus* | Walrus | XM_012560971.1 | NW_004450296.1 | NW_004450425.1 | XM_004398471.2 | NW_004450751.1 | XM_012566125.1 | XM_004412550.1 | NW_004450361.1 |
|  | *Ailuropoda melanoleuca* | Giant panda | ENSAMEG00000018420 | ENSAMET00000002595 | NW_003217607.1 | XM_034646956.1 | ENSAMEG00000014831 | XM_034672038.1 | ENSAMEG00000002720 | XM_019802824.2 |
|  | *Ursus maritimus* | Polar bear | XM_008701486.1 | XM_008711954.1 | XM_008700418.1 | XM_040629987.1 | XM_008690278.1 | XM_015144956.1 | XM_008705646.1 | XM_040638995.1 |
|  | *Canis familiaris* | Dog | ENSCAFG00000002140 | ENSCAFT00000044767 | ENSCAFG00000002223 | ENSCAFG00000016984 | ENSCAFG00000012433 | XM_038666585.1 | ENSCAFG00000001797 | ENSCAFG00000009379 |
| Chiroptera | *Pteropus vampyrus* | Ying fox | ENSPVAG00000008960 | XM_011374161.2 | XM_011383733.2 | XM_011358911.2 | XM_023522928.1 | XM_023535591.1 | ENSPVAG00000001590 | ENSPVAG00000003527 |
|  | *Myotis lucifugus* | Little brown bat | ENSMLUG00000010007 | ENSMLUT00000008520 | - | ENSMLUG00000003612 | ENSMLUG00000017783 | XM_023744252.1 | ENSMLUG00000016284 | ENSMLUG00000014733 |
| Eulipotyphla | *Sorex araneus* | [Shrew](http://asia.ensembl.org/Sorex_araneus) | ENSSARG00000002584 | XM_055141351.1 | XM_055120125.1 | XM_004398471.2 | - | - | XM_055118515.1 | XM_055142546.1 |
|  | *Erinaceus europaeus* | Hedgehog | XM_060188041.1 | XM_060177126.1 | XM_060187436.1 | XM_016188301.2 | - | - | XM_060194345.1 | XM_007519095.3 |
| Primates | *Pan troglodytes* | Chimpanzee | ENSPTRG00000016072 | NC_006478.4 | ENSPTRG00000012287 | XM_016932463.3 | XM_016950824.3 | ENSPTRG00000000099 | ENSPTRG00000005396 | ENSPTRG00000003537 |
|  | *Macaca mulatta* | Rhesus macaque | XM_015138678.2 | NC_027906.1 | ENSMMUG00000009871 | ENSMMUG00000021798 | XM_028831291.1 | XM_028840252.1 | ENSMMUG00000021808 | ENSMMUG00000002453 |
|  | *Callithrix jacchus* | Marmoset | ENSCJAG00000018261 | ENSCJAT00000019941 | ENSCJAG00000021202 | XM_035299970.2 | ENSCJAG00000000428 | ENSCJAG00000000288 | ENSCJAG00000015099 | ENSCJAG00000011551 |
|  | *Tupaia chinensis* | Chinese tree shrew | XM_014583885.2 | XM_014584763.2 | XM_014583397.2 | XM_006151752.3 | XM_006151940.1 | XM_014586595.2 | XM_006170352.2 | XM_006157843.3 |
| Rodentia | *Rattus norvegicus* | [Rat](http://asia.ensembl.org/Rattus_norvegicus) | ENSRNOG00000002175 | ENSRNOT00000046313 | ENSRNOG00000013408 | ENSRNOG00000007387 | NM_031678.2 | NM_023978.2 | NM_198750.2 | ENSRNOG00000007478 |
|  | *Ictidomys tridecemlineatus* | [Squirrel](http://asia.ensembl.org/Ictidomys_tridecemlineatus) | ENSSTOG00000016179 | ENSSTOT00000026164 | ENSSTOG00000008934 | ENSSTOG00000005366 | ENSSTOG00000008549 | ENSSTOG00000014007 | ENSSTOG00000020498 | ENSSTOG00000011521 |
| Lagomorpha | *Oryctolagus cuniculus* | [Rabbit](http://asia.ensembl.org/Oryctolagus_cuniculus) | XM_051820257.1 | XM_051851658.1 | XM_051835536.1 | XM_051825198.1 | XM_017339603.2 | - | ENSOCUG00000013721 | ENSOCUG00000025433 |
| Xenarthra | *Choloepus didactylus* | two-toed sloth | XM_037829837.1 | XM_037839901.1 | XM_037806899.1 | XM_037808461.1 | XM_037849250.1 | XM_037828621.1 | XM_037846569.1 | XM_037839049.1 |
| Afrotheria | *Trichechus manatus* | Manatee | XM_023737328.1 | NW_004443942.1 | NW_004443940.1 | NW_004443976.1 | XM_023740850.1 | XM_023739377.1 | NW_004443963.1 | NW_004444127.1 |
|  | *Loxodonta africana* | African elephant | ENSLAFG00000009558 | ENSLAFT00000037306 | ENSLAFG00000016336 | ENSLAFG00000012222 | ENSLAFG00000010995 | XM_023552103.1 | XM_003405313.3 | XM_003412109.3 |
| Marsupialia | *Monodelphis domestica* | short-tailed opossum | XM_007496439.3 | XM_056802039.1 | XM_007501137.3 | XM_007483139.3 | XM_056819961 | XM_007492108.2 | XM_056798810.1 | XM_007497374.3 |
