# Supplementary material for: Evolution of canonical circadian clock genes underlies unique sleep strategies of marine mammals for secondary aquatic adaptation
Source: PLoS Genet. 2025 Mar 18;21(3):e1011598. doi: 10.1371/journal.pgen.1011598 (PMC11919277; doi:10.1371/journal.pgen.1011598)
Supplement: S3 Table — (DOCX) [file pgen.1011598.s019.docx]

Table S3 The overall evolutionary distance between amino acid sequences for eight circadian clock genes.

| **Gene ^a^** | **Overall arerage distance** | **Maximum sequence similarity**  **(%)** | **Minimum sequence similarity**  **(%)** |
| --- | --- | --- | --- |
| *BMAL1* | 0.020 | 93.57 | 100.00 |
| *CRY1* | 0.022 | 91.26 | 100.00 |
| *CLOCK* | 0.046 | 84.92 | 100.00 |
| *CRY2* | 0.047 | 85.48 | 100.00 |
| *PER1* | 0.087 | 77.48 | 99.92 |
| *NPAS2* | 0.116 | 73.07 | 100.00 |
| *PER2* | 0.261 | 57.69 | 98.99 |
| *PER3* | 0.310 | 40.87 | 99.42 |

^a^ Rank the following in order of evolutionary distance smallest to largest.
