# Supplementary material for: Evolution of canonical circadian clock genes underlies unique sleep strategies of marine mammals for secondary aquatic adaptation
Source: PLoS Genet. 2025 Mar 18;21(3):e1011598. doi: 10.1371/journal.pgen.1011598 (PMC11919277; doi:10.1371/journal.pgen.1011598)
Supplement: S4 Table — (DOCX) [file pgen.1011598.s020.docx]

Table S4 Results of PAML branch-site model analysis of circadian genes in all-mammals dataset.

| **Genes** | **Branch-site model** | **-lnL** | | **2ΔlnL** | ***p* value** | | **Adjusted**  ***p* value** | **ω value** | **Positively selected sites^a^** |
| --- | --- | --- | --- | --- | --- | --- | --- | --- | --- |
| *CLOCK* | Branch (*Delphinapterus leucas*) | | | | |  | |  |  |
|  | Null | 14346.188 | |  |  | |  | ω_0_ = 0.051, ω_1_ = 1,  ω_2_ = 1 |  |
|  | Alternative | 14342.256 | | 7.864 | **0.005** | | 0.403 | ω_0_ = 0.051, ω_1_ = 1,  ω_2_ = 1.905 | 598(0.933) |
| *NPAS2* | Branch (LCA of Mysticeti) | | | | |  | |  |  |
|  | Null | 18967.861 | |  |  | |  | ω_0_ = 0.070, ω_1_ = 1,  ω_2_ = 1 |  |
|  | Alternative | 18965.866 | | 3.990 | **0.046** | | 1.000 | ω_0_ = 0.070, ω_1_ = 1,  ω_2_ = 28.005 |  |
|  | Branch (LCA of Cetacea) | | | | |  | |  |  |
|  | Null | 18967.300 | |  |  | |  | ω_0_ = 0.070, ω_1_ = 1,  ω_2_ = 1 |  |
|  | Alternative | 18965.099 | | 4.402 | **0.036** | | 1.000 | ω_0_ = 0.070, ω_1_ = 1,  ω_2_ = 6.445 | 131(0.985)* 748( 0.986)* |
| *CRY1* | Branch (*Tursiops aduncus*) | | | | |  | |  |  |
|  | Null | 8934.197 | |  |  | |  | ω_0_ = 0.023, ω_1_ = 1,  ω_2_ = 1 |  |
|  | Alternative | 8930.447 | | 7.501 | **0.006** | | 0.421 | ω_0_ = 0.023, ω_1_ = 1,  ω_2_ = 2.976 | 503(0.972)* |
| *PER1* | Branch (*Equus caballus*) | | | | |  | |  |  |
|  | Null | 30571.258 | |  |  | |  | ω_0_ = 0.057, ω_1_ = 1,  ω_2_ = 1 |  |
|  | Alternative | 30569.048 | | 4.421 | **0.036** | | 1.000 | ω_0_ = 0.057, ω_1_ = 1,  ω_2_ = 12.312 | 809(0.982)* 1069(0.971)* |
|  | Branch (LCA of Placentalia) | | | | |  | |  |  |
|  | Null | 30573.456 | |  |  | |  | ω_0_ = 0.056, ω_1_ = 1,  ω_2_ = 1 |  |
|  | Alternative | 30576.073 | | 5.235 | **0.022** | | 0.908 | ω_0_ = 0.057, ω_1_ = 1,  ω_2_ = 2.241 |  |
| *PER2* | Branch (*T. aduncus*) | |  | | |  | |  |  |
|  | Null | 40229.028 | |  |  | |  | ω_0_ = 0.132, ω_1_ = 1,  ω_2_ = 1 |  |
|  | Alternative | 40225.131 | | 5.106 | **0.024** | | 0.916 | ω_0_ = 0.132, ω_1_ = 1,  ω_2_ = 8.184 |  |
|  | Branch (*Stenella coeruleoalba*) | | | | |  | |  |  |
|  | Null | 40224.579 | |  |  | |  | ω_0_ = 0.132, ω_1_ = 1,  ω_2_ = 1 |  |
|  | Alternative | 40222.026 | | 7.793 | **0.005** | | 0.403 | ω_0_ = 0.132, ω_1_ = 1,  ω_2_ = 10.363 |  |
|  | Branch (*Loxodonta africana*) | | | | |  | |  |  |
|  | Null | 40226.359 | |  |  | |  | ω_0_ = 0.131, ω_1_ = 1,  ω_2_ = 1 |  |
|  | Alternative | 40223.155 | | 6.409 | **0.011** | | 0.582 | ω_0_ = 0.131, ω_1_ = 1,  ω_2_ = 9.303 | 346(0.911) 561(0.877) 936(0.884) |
| *PER3* | Branch (*Odobenus rosmarus*) | | | | |  | |  |  |
|  | Null | 37993.672 | |  |  | |  | ω_0_ = 0.192, ω_1_ = 1,  ω_2_ = 1 |  |
|  | Alternative | 37988.593 | | 10.157 | **0.001** | | 0.221 | ω_0_ = 0.193, ω_1_ = 1,  ω_2_ = 10.492 | 619(0.984)* 620(0.987)*  624(0.967)* |
|  | Branch (*D. leucas*) | | | | | | |  |  |
|  | Null | 37994.166 | |  |  | |  | ω_0_ = 0.194, ω_1_ = 1,  ω_2_ = 1 |  |
|  | Alternative | 37992.543 | | 5.246 | **0.022** | | 0.912 | ω_0_ = 0.194, ω_1_ = 1,  ω_2_ = 12.960 |  |
|  | Branch (*Physeter catodon*) | | | | | | |  |  |
|  | Null | 37997.654 | |  |  | |  | ω_0_ = 0.194, ω_1_ = 1,  ω_2_ = 1 |  |
|  | Alternative | 37995.368 | | 4.571 | **0.033** | | 1.000 | ω_0_ = 0.194, ω_1_ = 1,  ω_2_ = 5.206 | 387(0.966)* 395(0.847) 398(0.816) 420(0.817) 430(0.826) 438(0.836) 447(0.823) 508(0.844) 510(0.818) 520(0.829) 615(0.835) 652(0.842) 735(0.844) |
|  | Branch (LCA of *P. catodon* and *Kogia sima*) | | | | | | |  |  |
|  | Null | 38001.179 | |  |  | |  | ω_0_ = 0.196, ω_1_ = 1,  ω_2_ = 1 |  |
|  | Alternative | 37997.052 | | 8.253 | **0.004** | | 0.403 | ω_0_ = 0.196. ω_1_ = 1,  ω_2_ = 24.497 |  |
|  | Branch (LCA of *Balaenoptera acutorostrata* and *B. omurai*) | | | | | | |  |  |
|  | Null | 38000.887 | |  |  | |  | ω_0_ = 0.196, ω_1_ = 1,  ω_2_ = 1 |  |
|  | Alternative | 37998.712 | | 4.349 | **0.037** | | 1.000 | ω_0_ = 0.196, ω_1_ = 1,  ω_2_ = 47.979 |  |
|  | Branch (LCA of Cetacea) | | | | | | |  |  |
|  | Null | 38001.179 | |  |  | |  | ω_0_ = 0.196, ω_1_ = 1,  ω_2_ = 1 |  |
|  | Alternative | 37994.875 | | 12.608 | **< 0.001** | | 0.118 | ω_0_ = 0.196, ω_1_ = 1,  ω_2_ = 33.784 |  |
|  | Branch (*E. caballus*) | | | | | | |  |  |
|  | Null | 38000.219 | |  |  | |  | ω_0_ = 0.196, ω_1_ = 1,  ω_2_ = 1 |  |
|  | Alternative | 37995.857 | | 8.724 | **0.003** | | 0.386 | ω_0_ = 0.196, ω_1_ = 1,  ω_2_ = 47.979 | 62(0.937) 654(0.857) 756(0.944) |
|  | Branch (*Ailuropoda melanoleuca*) | | | | | | |  |  |
|  | Null | 37994.578 | |  |  | |  | ω_0_ = 0.194, ω_1_ = 1,  ω_2_ = 1 |  |
|  | Alternative | 37980.512 | | 28.133 | **< 0.001** | | **< 0.001** | ω_0_ = 0.194, ω_1_ = 1,  ω_2_ = 51.776 | 49(0.847) 86(0.848) 129(0.867) 130(0.830) 192(0.882) 194(0.994)** 196(0.988)* 233(0.838) 253(0.843) 284(0.839) 336(0.847) 358(0.836) 723(0.948) |
|  | Branch (*Ursus maritimus*) | | | | | | |  |  |
|  | Null | 37993.799 | |  |  | |  | ω_0_ = 0.193, ω_1_ = 1,  ω_2_ = 1 |  |
|  | Alternative | 37991.451 | | 4.697 | **0.030** | | 1.000 | ω_0_ = 0.193, ω_1_ = 1,  ω_2_ = 6.610 | 46(0.845) 81(0.994)** 88(0.844) 142(0.850) 144(0.896) 254(0.856) 273(0.847) 438(0.857) 474(0.858) 558(0.842) 981(0.807) 994(0.839) 1009(0.807) 1013(0.852)  1017(0.863) 1022(0.845)  1073(0.856) |
|  | Branch (LCA of *A. melanoleuca* and *U. maritimus*) | | | | | | |  |  |
|  | Null | 37990.780 | |  |  | |  | ω_0_ = 0.191, ω_1_ = 1,  ω_2_ = 1 |  |
|  | Alternative | 37985.068 | | 11.423 | **0.001** | | 0.149 | ω_0_ = 0.192, ω_1_ = 1,  ω_2_ = 13.855 | 106(0.863) 122(0.806) 154(0.818) 183(0.829) 228(0.819) 231(0.832) 254(0.820) 289(0.834) 296(0.839) 346(0.824) 362(0.826) 447(0.815) 580(0.801) 723(0.977)* 78(0.821) 969(0.807) |

^a^ Codons identified by PAML as under positive selection along with Bayesian (BEB) analysis PPs for sites with P > 80% under branch-site models.

* indicate posterior probabilities ≥ 0.95; ** indicate posterior probabilities ≥ 0.99.
