# Supplementary material for: Evolution of canonical circadian clock genes underlies unique sleep strategies of marine mammals for secondary aquatic adaptation
Source: PLoS Genet. 2025 Mar 18;21(3):e1011598. doi: 10.1371/journal.pgen.1011598 (PMC11919277; doi:10.1371/journal.pgen.1011598)
Supplement: S5 Table — (DOCX) [file pgen.1011598.s021.docx]

Table S5 Summary of branch-site positive selection analysis using the aBSREL method implemented in HyPhy v2.5.58.

| **Gene** | **Branch** | ***p* value** | **ω distribution** | **Site**  **((ER ≥ 100)** | **Evidence ratio** | **Empirical BF** |
| --- | --- | --- | --- | --- | --- | --- |
| *CLOCK* | *Delphinapterus leucas* | 0.008 | ω_1_ = 0.033 (99.830%);  ω_2_ = 1.000e+5 (0.170%) | 598 | 82.703 | 348,714.805 |
|  |  |  |  | 761 | 0.503 | 359.087 |
|  | *Monodelphis domestica* | 0.036 | ω_1_ = 0.090 (99.208%);  ω_2_ = 609.900 (0.792%) | 2 | 2.805 | 1,255.976 |
|  |  |  |  | 627 | 1.635 | 125.500 |
|  |  |  |  | 762 | 4.701 | 2,716.607 |
| *NPAS2* | *Stenella coeruleoalba* | 0.017 | ω_1_ = 1.000e+10 (100.000%); |  |  |  |
|  | LCA of Cetacea | 0.019 | ω_1_ = 0.218 (99.720%);  ω_2_ = 634.200 (0.280%) |  |  |  |
| *CRY2* | *Loxodonta africana* | 0.015 | ω_1_ = 0.065 (99.308%);  ω_2_ = 23.870 (0.692%) | 9 | 1.148 | 150.855 |
|  |  |  |  | 16 | 14.154 | 85,442.589 |
|  |  |  |  | 544 | 5.592 | 23,259.603 |
| *PER1* | *Sus_scrofa* | 0.026 | ω_1_ = 0.095 (27.233%);  ω_2_ = 0.096 (72.411%);  ω_3_ = 2032 (0.356%) | 572 | 11.276 | 31,761.947 |
|  |  |  |  | 896 | 1.164 | 110.19 |
|  |  |  |  | 1130 | 1.365 | 3,369.746 |
|  |  |  |  | 1233 | 0.892 | 126.944 |
|  | *Pan troglodytes* | 0.006 | ω_1_ = 0.118 (99.720%);  ω_2_ = 703.300 (0.280%) | 215 | 2.096 | 1,498.573 |
|  |  |  |  | 1233 | 5.638 | 2,729.153 |
|  |  |  |  | 1235 | 33.275 | 32,877.957 |
| *PER2* | *Tursiops aduncus* | 0.027 | ω_1_ = 0.2458 (99.705%);  ω_2_ = 2.424e+4 (0.295%) | 70 | 2.716 | 732.704 |
|  |  |  |  | 653 | 0.736 | 709.54 |
|  |  |  |  | 1125 | 20.076 | 30,355.491 |
|  | *S. coeruleoalba* | 0.047 | ω_1_ = 1.000e+10 (100.000%); |  |  |  |
|  | *Rattus norvegicus* | 0.015 | ω_1_ = 0.132 (39.167%);  ω_2_ = 0.137 (59.758%);  ω_3_ = 761.0 (1.076%) | 704 | 1.055 | 192.664 |
|  |  |  |  | 705 | 1.016 | 213.778 |
|  |  |  |  | 535 | 2.111 | 667.813 |
|  |  |  |  | 998 | 17.825 | 9,846.331 |
|  |  |  |  | 1050 | 0.878 | 238.325 |
| *PER3* | *Odobenus rosmarus* | 0.005 | ω_1_ = 0.000 (80.577%);  ω_2_ = 5.311 (19.423%) | 259 | 1.076 | 4,335.704 |
|  |  |  |  | 591 | 4.192 | 1,230,464.649 |
|  |  |  |  | 619 | 4.235 | 2,401,809.221 |
|  |  |  |  | 620 | 4.058 | 2,174,175.129 |
|  |  |  |  | 624 | 3.972 | 369,752.38 |
|  |  |  |  | 885 | 4.164 | 1,391,807.344 |
|  | *Physeter catodon* | 0.030 | ω_1_ = 0.751 (99.526%);  ω_2_ = 109.500 (0.474%) | 55 | 5.495 | 1,487.861 |
|  |  |  |  | 126 | 2.351 | 504.211 |
|  |  |  |  | 831 | 2.585 | 610.667 |
|  |  |  |  | 1028 | 4.147 | 1,070.610 |
|  | LCA of *P. catodon* and *Kogia sim* | 0.001 | ω_1_ = 0.000 (99.884%);  ω_2_ = 887.600 (0.116%) | 223 | 190.438 | 21,067,897.734 |
|  | LCA of Cetacea | 0.035 | ω_1_ = 0.000 (93.079%);  ω_2_ = 7.344 (6.9213%) | 942 | 3.699 | 550.578 |
|  | *Ailuropoda melanoleuca* | 0.004 | ω_1_ = 0.659 (98.100%);  ω_2_ = 44.790 (1.900%) | 192 | 11.239 | 1,356.549 |
|  |  |  |  | 194 | 5.676 | 684.641 |
|  |  |  |  | 196 | 6.846 | 841.463 |
|  | *Ursus maritimus* | 0.004 | ω_1_ = 1.000 (99.516%);  ω_2_ = 238.600 (0.484%) | 81 | 9.043 | 2,146.877 |
|  |  |  |  | 144 | 8.662 | 2,035.662 |
|  |  |  |  | 981 | 2.205 | 365.426 |
|  | LCA of *A. melanoleuca* and *U. maritimus* | 0.008 | ω_1_ = 0.653 (98.442%);  ω_2_ = 2.424e+4 (1.558%) | 11 | 1.257 | 128.979 |
|  |  |  |  | 13 | 1.002 | 451.458 |
|  |  |  |  | 33 | 2.122 | 174.075 |
|  |  |  |  | 106 | 1.293 | 115.354 |
|  |  |  |  | 808 | 1.139 | 175.25 |
|  |  |  |  | 976 | 4.864 | 487.408 |
|  |  |  |  | 1096 | 0.941 | 631.105 |
|  |  |  |  | 1103 | 1.082 | 106.752 |
