# Supplementary material for: Evolution of canonical circadian clock genes underlies unique sleep strategies of marine mammals for secondary aquatic adaptation
Source: PLoS Genet. 2025 Mar 18;21(3):e1011598. doi: 10.1371/journal.pgen.1011598 (PMC11919277; doi:10.1371/journal.pgen.1011598)
Supplement: S6 Table — (DOCX) [file pgen.1011598.s022.docx]

Table S6 Summary of branch-site positive selection analysis using the BUSTED method implemented in HyPhy v2.5.58.

| **Gene** | **Branch** | **Model** | **Parameters** | **-lnL** | **AICc** | ***p* value** | **Rate distribution** | | | **Site**  **(ER ≥ 10)** |
| --- | --- | --- | --- | --- | --- | --- | --- | --- | --- | --- |
|  |  |  |  |  |  |  | **ω_1_** | **ω_2_** | **ω3** |  |
| *CLOCK* | *Delphinapterus leucas* | Unconstrained model | 102 | 14056.6 | 28317.8 | 0.005 | 0.033 (93.554%) | 0.245 (6.309%) | 1.000e+10 (0.137%) | 598(802.125) |
|  |  | Constrained model | 101 | 14061.1 | 28324.9 |  | 0.000 (90.717%) | 1.000 (4.251%) | 1.000 (5.032%) |  |
|  | *Monodelphis domestica* | Unconstrained model | 102 | 14055.8 | 28316.2 | 0.005 | 0.0656 (99.299%) | 0.065 (0.054%) | 1553 (0.647%) | 2(58.705) |
|  |  | Constrained model | 101 | 14060.8 | 28324.3 |  | 0.061 (98.276%) | 0.061 (0.046%) | 1.000 (1.68%) | 762(11.639) |
| *NPAS2* | *Trichechus manatus* | Unconstrained model | 101 | 18738.7 | 37680.0 | 0.010 | 0.259 (82.077%) | 0.260 (17.458%) | 351.3 (0.465%) | 692(20.398) |
|  |  | Constrained model | 100 | 18742.6 | 37685.8 |  | 0.295 (0.000%) | 0.213 (90.392%) | 1.000 (9.608%) | 778(19.788) |
|  | LCA of Cetacea | Unconstrained model | 101 | 18738.7 | 37680.1 | 0.043 | 0.198 (98.557%) | 0.198 (1.084%) | 104.6 (0.359%) | 131(32.714) |
|  |  | Constrained model | 100 | 18741.2 | 37683.0 |  | 0.193 (86.600%) | 0.142 (10.153%) | 1.000 (3.247%) |  |
| *PER2* | *Stenella coeruleoalba* | Unconstrained model | 100 | 39815.3 | 79831.1 | 0.042 | 0.141 (91.359%) | 0.140 (6.308%) | 6.128 (2.333%) | 603(36.058) |
|  |  | Constrained model | 99 | 39817.8 | 79834.1 |  | 0.119 (71.143%) | 0.116 (20.914%) | 1.000 (7.943%) | 626(35.754) |
|  |  |  |  |  |  |  |  |  |  | 818(35.762) |
|  |  |  |  |  |  |  |  |  |  | 1109(36.305) |
|  |  |  |  |  |  |  |  |  |  | 1125(11.793) |
| *PER3* | *Odobenus rosmarus* | Unconstrained model | 98 | 37801.1 | 75798.8 | 0.020 | 0.317 (40.247%) | 0.829 (55.919%) | 13.28 (3.834%) |  |
|  |  | Constrained model | 97 | 37804.4 | 75803.2 |  | 0.000 (21.393%) | 1.000 (67.166%) | 1.000 (11.440%) |  |
|  | *Physeter catodon* | Unconstrained model | 98 | 37801.3 | 75799.2 | 0.032 | 0.995 (25.366%) | 0.966 (74.122%) | 147.6 (0.512%) | 126(11.129) |
|  |  | Constrained model | 97 | 37804.4 | 75803.3 |  | 1.000 (62.122%) | 1.000 (29.035%) | 1.000 (8.842%) | 1028(10.037) |
|  | LCA of *P. catodon* and *Kogia sim* | Unconstrained model | 98 | 37802.7 | 75801.9 | 0.005 | 0.000 (69.175%) | 0.000 (30.614%) | 597.2 (0.211%) | 223(958.121) |
|  |  | Constrained model | 97 | 37807.3 | 75809.1 |  | 0.000 (66.712%) | 0.000 (4.401%) | 1.000 (28.886%) |  |
|  | LCA of *Balaenoptera acutorostrata* and *B. omurai* | Unconstrained model | 98 | 37800.2 | 75796.8 | 0.048 | 0.515 (12.383%) | 0.516 (85.613%) | 13.28 (2.004%) | 199(125.472) |
|  |  | Constrained model | 97 | 37802.5 | 75799.5 |  | 0.322 (39.488%) | 0.338 (22.031%) | 1.000 (38.480%) | 374(30.921) |
|  |  |  |  |  |  |  |  |  |  | 587(14.937) |
|  | *Equus caballus* | Unconstrained model | 98 | 37804.2 | 75804.9 | 0.011 | 0.305 (35.637%) | 0.495 (63.824%) | 3.200e+4 (0.539%) | 62(56.699) |
|  |  | Constrained model | 97 | 37808.1 | 75810.6 |  | 0.216 (54.852%) | 0.279 (15.602%) | 1.000 (29.546%) |  |
|  | *Ailuropoda melanoleuca* | Unconstrained model | 98 | 37793.5 | 75783.5 | < 0.001 | 1.000 (28.732%) | 1.000 (69.218%) | 144.4 (2.050%) | 192(42.377) |
|  |  | Constrained model | 97 | 37805.7 | 75805.9 |  | 1.000 (27.423%) | 1.000 (67.279%) | 1.000 (5.298%) | 194(45.356) |
|  |  |  |  |  |  |  | 1.000 (34.735%) | 1.000 (64.879%) | 1548 (0.386%) | 196(88.731) |
|  | *Ursus maritimus* | Unconstrained model | 98 | 37796.5 | 75789.5 | 0.001 | 1.000 (33.257%) | 1.000 (63.728%) | 1.000 (3.015%) | 81(59.848) |
|  |  | Constrained model | 97 | 37802.6 | 75799.6 |  | 0.393 (62.345%) | 0.482 (30.456%) | 12.010 (7.199%) | 144(67.027) |
|  | LCA of *A. melanoleuca* and *U. maritimus* | Unconstrained model | 98 | 37798.4 | 75793.3 | < 0.001 | 1.000 (15.652%) | 0.000 (29.224%) | 1.000 (55.124%) | 13(12.766) |
|  |  | Constrained model | 97 | 37804.9 | 75804.4 |  |  |  |  | 106(10.695) |
|  |  |  |  |  |  |  |  |  |  | 976(10.506) |
