# Supplementary material for: Evolution of canonical circadian clock genes underlies unique sleep strategies of marine mammals for secondary aquatic adaptation
Source: PLoS Genet. 2025 Mar 18;21(3):e1011598. doi: 10.1371/journal.pgen.1011598 (PMC11919277; doi:10.1371/journal.pgen.1011598)
Supplement: S7 Table — (DOCX) [file pgen.1011598.s023.docx]

Table S7 The SWS/TST ratios in mammals.

| **Species** | **SWS/TST** |
| --- | --- |
| *Tursiops truncatus* | 100.00 |
| *Bos taurus* | 81.11 |
| *Ovis aries* | 85.19 |
| *Sus scrofa* | 70.87 |
| *Equus caballus* | 72.57 |
| *Leptonychotes weddellii* | 85.02 |
| *Odobenus rosmarus* | 91.67 |
| *Canis familiaris* | 82.04 |
| *Myotis lucifugus* | 90.02 |
| *Sorex araneus* | 84.62 |
| *Erinaceus europaeus* | 71.43 |
| *Pan troglodytes* | 85.0 |
| *Macaca mulatta* | 88.96 |
| *Callithrix jacchus* | 83.15 |
| *Tupaia chinensis* | 83.60 |
| *Rattus norvegicus* | 80.52 |
| *Ictidomys tridecemlineatus* | 75.29 |
| *Oryctolagus cuniculus* | 87.09 |
| *Choloepus didactylus* | 88.70 |
| *Trichechus manatus* | 96.43 |
| *Monodelphis domestica* | 78.06 |
