# Supplementary material for: Evolution of canonical circadian clock genes underlies unique sleep strategies of marine mammals for secondary aquatic adaptation
Source: PLoS Genet. 2025 Mar 18;21(3):e1011598. doi: 10.1371/journal.pgen.1011598 (PMC11919277; doi:10.1371/journal.pgen.1011598)
Supplement: S8 Table — (DOCX) [file pgen.1011598.s024.docx]

Table S8 The PGLS analyses between the root-to-tip ω and SWS/TST in all mammals.

| **Gene** | ***P* value** | **R^2^** | **λ** |
| --- | --- | --- | --- |
| *BMAL1* | 0.871 | 0.004 | 0 |
| *CLOCK* | 0.558 | 0.025 | 0 |
| *CRY1* | 0.004 | 0.378 | 1 |
| *CRY2* | 0.182 | 0.123 | 0 |
| *NPAS2* | 0.131 | 0.155 | 0 |
| *PER1* | 0.014 | 0.266 | 1 |
| *PER2* | 0.085 | 0.175 | 1 |
| *PER3* | 0.533 | 0.028 | 1 |
