# Supplementary material for: Evolution of canonical circadian clock genes underlies unique sleep strategies of marine mammals for secondary aquatic adaptation
Source: PLoS Genet. 2025 Mar 18;21(3):e1011598. doi: 10.1371/journal.pgen.1011598 (PMC11919277; doi:10.1371/journal.pgen.1011598)
Supplement: S9 Table — (DOCX) [file pgen.1011598.s025.docx]

Table S9 Functional effect of USWS group-specific amino acid changes.

| **Gene** | **UniProt Accession ID** | **Convergent amino acid substitutions** | **PolyPhen-2** | | **SIFT** | | | **PROVEAN** | | |
| --- | --- | --- | --- | --- | --- | --- | --- | --- | --- | --- |
|  |  |  | **Score** | **Prediction** | | **Score** | **Prediction**  **(cutoff = 0.05)** | | **Score** | **Prediction**  **(cutoff = -2.5)** |
| unique substitution shared by cetaceans and manatee | | | | | | | | | | |
| *NPAS2* | Q99743 | T699P | 0.800 | possibly damaging | | 0.012 | Damaging | | -0.77 | Neutral |
| unique substitution shared by cetaceans and walrus | | | | | | | | | | |
| *PER2* | O15055 | K127R | 0.100 | benign | | 1.000 | Tolerated | | -0.53 | Neutral |
| unique substitution shared by cetaceans and walrus | | |  |  | |  |  | |  |  |
| *PER3* | P56645 | I636V | 0.176 | benign | | 0.030 | Damaging | | -0.82 | Neutral |
