# Supplementary material for: Evolution of canonical circadian clock genes underlies unique sleep strategies of marine mammals for secondary aquatic adaptation
Source: PLoS Genet. 2025 Mar 18;21(3):e1011598. doi: 10.1371/journal.pgen.1011598 (PMC11919277; doi:10.1371/journal.pgen.1011598)
Supplement: S10 Table — (DOCX) [file pgen.1011598.s026.docx]

Table S10 Functional effect of cetacean-specific amino acid replacements.

| **Gene** | **UniProt Accession ID** | **Position** | **From** | **To** | **PolyPhen-2** | | **SIFT** | | **PROVEAN** | |
| --- | --- | --- | --- | --- | --- | --- | --- | --- | --- | --- |
|  |  |  |  |  | **Score** | **Prediction** | **Score** | **Prediction**  **(cutoff = 0.05)** | **Score** | **Prediction**  **(cutoff = -2.5)** |
| *BMAL1* | O00327 | 3 | D | E | 0.984 | probably damaging | 0.631 | Tolerated | 0.19 | Neutral |
|  |  | 456 | L | P | 0.165 | benign | 0.277 | Tolerated | 0.59 | Neutral |
|  |  | 461 | H | R | 0.000 | benign | 0.504 | Tolerated | 0.00 | Neutral |
|  |  | 466 | M | T | 0.001 | benign | 0.439 | Tolerated | 0.01 | Neutral |
| *CLOCK* | O15516 | 724 | M | V | 0.730 | possibly damaging | 0.016 | Damaging | -1.20 | Neutral |
|  |  | 752 | S | P | 0.000 | benign | 0.200 | Tolerated | 0.01 | Neutral |
|  |  | 779 | T | A | 0.000 | benign | 0.310 | Tolerated | -0.68 | Neutral |
| *NPAS2* | Q99743 | 131 | N | R | 0.999 | probably damaging | 0.002 | Damaging | -4.85 | Deleterious |
|  |  | 246 | E | K | 0.992 | probably damaging | 0.052 | Tolerated | -3.12 | Deleterious |
|  |  | 381 | D | H | 0.960 | probably damaging | 0.005 | Damaging | -2.20 | Neutral |
|  |  | 431 | T | G | 0.000 | benign | 1.000 | Tolerated | -0.34 | Neutral |
|  |  | 438 | S | G | 0.658 | possibly damaging | 0.024 | Damaging | -2.45 | Neutral |
|  |  | 712 | F | C | 0.999 | probably damaging | 0.007 | Damaging | -1.45 | Neutral |
|  |  | 721 | N | T | 0.003 | benign | 0.906 | Tolerated | 0.07 | Neutral |
|  |  | 761 | H | C | 0.941 | possibly damaging | 0.020 | Damaging | -2.26 | Neutral |
| *CRY1* | Q16526 | 532 | Q | P | 0.475 | possibly damaging | 0.048 | Damaging | 0.41 | Neutral |
| *CRY2* | Q49AN0 | 564 | E | G | 0.319 | benign | 0.019 | Damaging | -0.77 | Neutral |
| *PER1* | O15534 | 85 | E | D | 0.004 | benign | 0.034 | Damaging | -1.57 | Neutral |
|  |  | 529 | D | A | 0.997 | probably damaging | 0.015 | Damaging | -5.91 | Damaging |
|  |  | 593 | P | A | 0.000 | benign | 0.457 | Tolerated | 0.03 | Neutral |
|  |  | 771 | D | G | 0.997 | probably damaging | 0.023 | Damaging | -2.05 | Neutral |
|  |  | 823 | H | R | 0.004 | benign | 0.801 | Tolerated | -0.29 | Neutral |
|  |  | 844 | H | Q | 0.976 | probably damaging | 0.632 | Tolerated | -1.16 | Neutral |
|  |  | 884 | Y | C | 0.002 | benign | 0.005 | Damaging | -4.35 | Damaging |
|  |  | 1019 | E | D | 0.000 | benign | 0.372 | Tolerated | -0.47 | Neutral |
|  |  | 1022 | A | E | 0.048 | benign | 0.402 | Tolerated | -1.42 | Neutral |
|  |  | 1027 | V | I | 0.000 | benign | 0.348 | Tolerated | -0.51 | Neutral |
|  |  | 1079 | G | S | 0.126 | benign | 0.930 | Tolerated | -0.06 | Neutral |
| *PER2* | O15055 | 136 | A | L | 0.979 | probably damaging | 0.181 | Tolerated | -3.50 | Deleterious |
|  |  | 220 | D | G | 0.571 | possibly damaging | 0.053 | Tolerated | -4.69 | Deleterious |
|  |  | 478 | G | S | 0.991 | probably damaging | 0.178 | Tolerated | -2.06 | Neutral |
|  |  | 488 | N | S | 0.952 | probably damaging | 0.105 | Tolerated | -2.57 | Deleterious |
|  |  | 502 | D | E | 0.365 | benign | 0.212 | Tolerated | -2.30 | Neutral |
|  |  | 503 | S | C | 1.000 | probably damaging | 0.003 | Damaging | -4.22 | Deleterious |
|  |  | 573 | C | F | 0.004 | benign | 0.700 | Tolerated | -2.13 | Neutral |
|  |  | 577 | P | L | 0.996 | probably damaging | 0.008 | Damaging | -4.41 | Deleterious |
|  |  | 764 | E | D | 0.344 | benign | 0.278 | Tolerated | -0.98 | Neutral |
|  |  | 1150 | T | A | 0.235 | benign | 0.005 | Damaging | -3.62 | Deleterious |
|  |  | 1210 | Y | C | 1.000 | probably damaging | 0.147 | Tolerated | -2.90 | Deleterious |
| *PER3* | P56645 | 53 | E | K | 0.999 | probably damaging | 0.879 | Tolerated | 1.08 | Neutral |
|  |  | 54 | E | D | 0.999 | probably damaging | 0.065 | Tolerated | -2.38 | Neutral |
|  |  | 156 | N | H | 1.000 | probably damaging | 0.339 | Tolerated | -1.61 | Neutral |
|  |  | 334 | P | L | 1.000 | probably damaging | 0.000 | Damaging | -4.76 | Deleterious |
|  |  | 636 | I | R | 0.957 | probably damaging | 0.003 | Damaging | -5.67 | Deleterious |
|  |  | 701 | S | Q | 0.999 | probably damaging | 0.004 | Damaging | -1.90 | Neutral |
|  |  | 704 | S | D | 0.935 | possibly damaging | 0.385 | Tolerated | -1.92 | Neutral |
|  |  | 928 | R | K | 0.842 | possibly damaging | 0.009 | Damaging | -4.21 | Deleterious |
