# Supplementary material for: Evolution of canonical circadian clock genes underlies unique sleep strategies of marine mammals for secondary aquatic adaptation
Source: PLoS Genet. 2025 Mar 18;21(3):e1011598. doi: 10.1371/journal.pgen.1011598 (PMC11919277; doi:10.1371/journal.pgen.1011598)
Supplement: S11 Table — (DOCX) [file pgen.1011598.s027.docx]

Table S11 Identified site changes link to protein function.

| **Gene** | **Site** | **AA change** | **Radical/Conservative changes** | **Functional region (identity with sites of human)** |
| --- | --- | --- | --- | --- |
| **Convergent mutations** | | | | |
| *NPAS2* | 699 | T-P | C | Region: Disordered: 739-824 |
| *PER2* | 127 | K-R | C | Motif: Nuclear export signal 1: 111-120 |
| *PER3* | 636 | I-V | C | Region: CSNK1E binding domain: 555-760 |
| **Cetacean-specific mutations** | | | | |
| *BMAL1* | 3 | D-E | C | Region: Disordered: 1-60 |
|  | 456 | L-P | R | Region: Disordered: 458-493 |
|  | 461 | H-R | C | Region: Disordered: 458-493 |
|  | 466 | M-T | R | Region: Disordered: 458-493 |
| *CLOCK* | 752 | S-P | C | Region: Disordered: 764-783 |
|  | 779 | T-A | R | Region: Disordered: 764-783 |
| *NPAS2* | 131 | N-R | R | Domain: PAS-A: 82-152 |
|  | 246 | E-K | R | Domain: PAS-B: 237-307 |
|  | 381 | D-H | R | Region: Disordered: 367-437 |
|  | 712 | F-C | R | Region: Disordered: 681-704 |
|  | 761 | H-C | R | Region: Disordered: 739-824 |
| *CRY2* | 564 | E-G | R | Site: Phosphoserine; by DYRK1A and MAPK: 558 |
|  |  |  |  | Region: C-terminal; Interaction with PER2: 493-592 |
| *PER1* | 85 | E-D | C | Region: Interaction with BTRC: 1-151 |
|  |  |  |  | Region: Disordered: 1-134 |
|  | 529 | D-A | R | Region: Disordered: 508-544 |
|  | 593 | P-A | R | Region: Required for phosphorylation by CSNK1E: 596-815 |
|  | 823 | H-R | C | Motif: Nuclear export signal: 827-843 |
|  |  |  |  | Region: Disordered: 805-874 |
|  | 844 | H-Q | R | Motif: Nuclear export signal: 827-843 |
|  |  |  |  | Region: Disordered: 805-874 |
|  | 1019 | E-D | C | Region: Disordered: 996-1037 |
|  | 1022 | A-E | R | Region: Disordered: 996-1037 |
|  | 1027 | V-I | C | Region: Disordered: 996-1037 |
|  | 1079 | G-S | R | Region: Disordered: 1051-1098 |
| *PER2* | 220 | D-G | R | Domain: PAS-A: 181-248 |
|  | 478 | G-S | R | Motif: Nuclear export signal 2: 462-471 |
|  |  |  |  | Region: Important for protein stability: 480-484 |
|  | 488 | N-S | C | Region: Important for protein stability: 480-484 |
|  | 573 | C-F | R | Region: CSNK1E binding domain: 512-717 |
|  | 577 | P-L | R | Region: CSNK1E binding domain: 512-717 |
|  | 764 | E-D | C | Region: Disordered: 764-838 |
|  | 1150 | T-A | R | Region: CRY binding domain: 1155-1255 |
|  | 1210 | Y-C | R | Region: CRY binding domain: 1155-1255 |
| *PER3* | 53 | E-K | R | Motif: Nuclear export signal 1: 55-64 |
|  | 54 | E-D | C | Motif: Nuclear export signal 1: 55-64 |
|  | 156 | N-H | R | Domain: PAS-A: 121-188 |
|  | 334 | P-L | R | Domain: PAC: 337-380 |
|  | 636 | I-R | R | Region: CSNK1E binding domain: 555-760 |
|  | 701 | S-Q | C | Region: CSNK1E binding domain: 555-760 |
|  |  |  |  | Site: Phosphoserine: 700 |
|  | 704 | S-D | R | Region: CSNK1E binding domain: 555-760 |
|  | 928 | R-K | C | Motif: Nuclear export signal 2: 925-932 |
