# Supplementary material for: Evolution of canonical circadian clock genes underlies unique sleep strategies of marine mammals for secondary aquatic adaptation
Source: PLoS Genet. 2025 Mar 18;21(3):e1011598. doi: 10.1371/journal.pgen.1011598 (PMC11919277; doi:10.1371/journal.pgen.1011598)
Supplement: S12 Table — (DOCX) [file pgen.1011598.s028.docx]

Table S12 Structural alignments for the WT and mutant-type circadian clock proteins. Rows shaded in grey correspond to the TM-score < 0.5.

|  | **TM-alignment** | | | | **jFATCAT (rigid)** | | | | |
| --- | --- | --- | --- | --- | --- | --- | --- | --- | --- |
|  | | **RMSD** | **TM-score** | **Identity** | | **RMSD** | **TM-score** | **Identity** | |
| **Convergent mutations** | | | | | | | | | |
| ***NAPS2*** | | | | | | | | | |
| WT-*Homo sapiens*;  Mutant-type (T699P) | | 4.64 | 0.57 | 82% | | 13.26 | 0.56 | 74% | |
| WT-*Mus musculus*;  Mutant-type (T703P) | | 4.39 | 0.57 | 82% | | 13.39 | 0.5 | 79% | |
| WT-*Bos taurus*;  Mutant-type (T689P) | | 3.83 | 0.6 | 85% | | 15.32 | 0.57 | 73% | |
| WT-*Tursiops truncatus*;  Mutant-type (P691T) | | 5.27 | 0.61 | 72% | | 13.52 | 0.56 | 62% | |
| WT-*Loxodonta africana*;  Mutant-type (T699P) | | 4.47 | 0.7 | 82% | | 12.07 | 0.68 | 71% | |
| WT-*Trichechus manatus*;  Mutant-type (P663T) | | 4.92 | 0.65 | 72% | | 15.4 | 0.58 | 58% | |
| ***PER2*** | | | | | | | | | |
| WT-*Homo sapiens*;  Mutant-type (K127R) | | 6.76 | 0.59 | 61% | | 14.68 | 0.61 | 54% | |
| WT-*Mus musculus*;  Mutant-type (K125R) | | 7.6 | 0.43 | 56% | | 19.54 | 0.4 | 59% | |
| WT-*Bos taurus*;  Mutant-type (K127R) | | 6.38 | 0.51 | 55% | | 16.68 | 0.51 | 57% | |
| WT-*Tursiops truncatus*;  Mutant-type (R127K) | | 6.78 | 0.55 | 72% | | 15.58 | 0.52 | 75% | |
| WT-*Leptonychotes weddellii*;  Mutant-type (K121R) | | 7.13 | 0.4 | 60% | | 18.47 | 0.38 | 52% | |
| WT-*Odobenus rosmarus*;  Mutant-type (R118K) | | 7.14 | 0.5 | 58% | | 16.66 | 0.48 | 56% | |
| ***PER3*** | | | | | | | | | |
| WT-*Homo sapiens*;  Mutant-type (I636V) | | 7 | 0.5 | 56% | | 16.51 | 0.5 | 41% | |
| WT-*Mus musculus*;  Mutant-type (S627V) | | 6.62 | 0.56 | 71% | | 15.06 | 0.56 | 69% | |
| WT-*Ovis aries*;  Mutant-type (L648V) | | 7.49 | 0.43 | 64% | | 18.42 | 0.41 | 44% | |
| WT-*Tursiops truncatus*;  Mutant-type (V654I) | | 6.13 | 0.51 | 75% | | 18.5 | 0.51 | 70% | |
| WT-*Leptonychotes weddellii*;  Mutant-type (I575V) | | 6.37 | 0.5 | 71% | | 16.65 | 0.52 | 64% | |
| WT-*Odobenus rosmarus*;  Mutant-type (V657I) | | 5.39 | 0.62 | 68% | | 13.14 | 0.58 | 52% | |
| **Cetacean-specific mutations** | | | | | | | | |  |
| ***BMAL1*** | |  |  |  | |  |  |  | |
| WT-*Homo sapiens*;  Mutant-type (D3E, L456P, H461R, M466T) | | 2.75 | 0.93 | 86% | | 2.81 | 0.92 | 83% | |
| WT-*Mus musculus*;  Mutant-type (D3E, L462P, H467R, M472T) | | 2.73 | 0.93 | 85% | | 2.79 | 0.93 | 85% | |
| WT-*Bos taurus*;  Mutant-type (D3E, L456P, H461R, M466T) | | 2.74 | 0.91 | 83% | | 3.05 | 0.91 | 83% | |
| WT-*Tursiops truncatus*;  Mutant-type (E3D, P456L, R461H, T466M) | | 3.96 | 0.84 | 80% | | 4.24 | 0.84 | 75% | |
| ***CLOCK*** | |  |  |  | |  |  |  | |
| WT-*Homo sapiens*;  Mutant-type (M724V, S752P, T779A) | | 7.12 | 0.47 | 78% | | 17.01 | 0.46 | 60% | |
| WT-*Mus musculus*;  Mutant-type (M720V, S748P, G788A) | | 6.46 | 0.42 | 87% | | 17.27 | 0.43 | 66% | |
| WT-*Bos taurus*;  Mutant-type (M725V, S753P, T778A) | | 7.24 | 0.46 | 71% | | 16.68 | 0.45 | 64% | |
| WT-*Tursiops truncatus*;  Mutant-type (V725M, P753S, A780T) | | 6.25 | 0.48 | 78% | | 16.34 | 0.48 | 64% | |
| ***NAPS2*** | |  |  |  | |  |  |  | |
| WT-*Homo sapiens*;  Mutant-type (N131R, E246K, D381H, T431G, S438G, F712C, N721T, H761C) | | 4.41 | 0.56 | 82% | | 14.98 | 0.48 | 62% | |
| WT-*Mus musculus*;  Mutant-type (N131R, E246K, E381H, T431G, S438G, F716C, N725T, P763C) | | 4.75 | 0.57 | 85% | | 15.69 | 0.5 | 62% | |
| WT-*Bos taurus*;  Mutant-type (N131R, E246K, E381H, T431G, S438G, F702C, S711T, H746C) | | 5.17 | 0.64 | 76% | | 11.37 | 0.6 | 71% | |
| WT-*Tursiops truncatus*;  Mutant-type (R131N, K246E, H381E, G431T, G438S, C704F, T713S, C748H) | | 4.32 | 0.59 | 80% | | 14.89 | 0.53 | 61% | |
| ***CRY1*** | |  |  |  | |  |  |  | |
| WT-*Homo sapiens*;  Mutant-type (Q532P) | | 2.24 | 0.9 | 96% | | 3.04 | 0.9 | 94% | |
| WT-*Mus musculus*;  Mutant-type (Q533P) | | 2.13 | 0.89 | 93% | | 3.36 | 0.89 | 90% | |
| WT-*Bos taurus*;  Mutant-type (Q532P) | | 1.85 | 0.89 | 97% | | 0.52 | 0.88 | 100% | |
| WT-*Tursiops truncatus*;  Mutant-type (P533Q) | | 1.82 | 0.87 | 96% | | 3.04 | 0.86 | 91% | |
| ***CRY2*** | |  |  |  | |  |  |  | |
| WT-*Homo sapiens*;  Mutant-type (E564G) | | 1.94 | 0.89 | 100% | | 2.24 | 0.89 | 100% | |
| WT-*Mus musculus*;  Mutant-type (E563G) | | 2.04 | 0.88 | 99% | | 3.95 | 0.88 | 93% | |
| WT-*Bos taurus*;  Mutant-type (E564G) | | 2.19 | 0.9 | 95% | | 2.93 | 0.9 | 95% | |
| WT-*Tursiops truncatus*;  Mutant-type (G564E) | | 2.14 | 0.96 | 93% | | 2.17 | 0.95 | 93% | |
| ***PER1*** | |  |  |  | |  |  |  | |
| WT-*Homo sapiens*;  Mutant-type (E85D, D529A, P593A, D771G, H823R, H844Q, Y884C, E1019D, E1021A, V1027I, G1079S) | | 6.05 | 0.52 | 71% | | 17.48 | 0.52 | 74% | |
| WT-*Mus musculus*;  Mutant-type (E85D, D529A, P593A, D771G, H820R, H841Q, Y883C, E1018D, E1020A, V1026I, G1078S) | | 6.33 | 0.66 | 61% | | 10.9 | 0.67 | 62% | |
| WT-*Bos taurus*;  Mutant-type (E87D, D531A, P595A, D773G, H825R, H846Q, Y886C, E1018D, E1020A, V1026I, G1076S) | | 7.31 | 0.5 | 66% | | 17.16 | 0.51 | 53% | |
| WT-*Tursiops truncatus*;  Mutant-type (D85E, A529D, A593P, G771D, R820H, Q841H, C881Y, D1001E, A1003E, I1009V, S1061G) | | 7.78 | 0.48 | 59% | | 17.32 | 0.47 | 55% | |
| ***PER2*** | |  |  |  | |  |  |  | |
| WT-*Homo sapiens*;  Mutant-type (A136L, D220G, G478S, N488S, D502E, S503C, C573F, P577L, E764D, T1150A, Y1210C) | | 7.48 | 0.55 | 56% | | 15.39 | 0.55 | 47% | |
| WT-*Mus musculus*;  Mutant-type (A134L, D218G, G476S, N486S, D500E, S501C, Y576F, P580L, E756D, T1152A, Y1212C) | | 7.2 | 0.61 | 56% | | 12.65 | 0.61 | 50% | |
| WT-*Bos taurus*;  Mutant-type (A136L, D220G, G478S, N488S, D502E, S503C, C578F, P582L, E770D, T1172A, Y1232C) | | 7.11 | 0.5 | 65% | | 16.8 | 0.51 | 50% | |
| WT-*Tursiops truncatus*;  Mutant-type (L136A, G220D, S478G, S488N, E502D, C503S, F578C, L582P, D769E, A1172T, C1232Y) | | 7.36 | 0.46 | 56% | | 17.23 | 0.41 | 40% | |
| ***PER3*** | |  |  |  | |  |  |  | |
| WT-*Homo sapiens*;  Mutant-type (E53K, E54D, N156H, P334L, S636R, K701Q, E704D, R928K) | | 6.44 | 0.4 | 69% | | 20.47 | 0.36 | 52% | |
| WT-*Mus musculus*;  Mutant-type (E52K, E53D, N155H, P329L, S619R, R683Q, E686D, R908K) | | 6.62 | 0.49 | 69% | | 16.74 | 0.48 | 59% | |
| WT-*Ovis aries*;  Mutant-type (D70K, E71D, N173H, P349L, S640R, K709Q, E712D, G931K) | | 6.32 | 0.53 | 65% | | 17.27 | 0.54 | 53% | |
| WT-*Tursiops truncatus*;  Mutant-type (K70E, D71E, H173N, L349P, R646S, Q715K, D718E, K942G) | | 6.91 | 0.42 | 70% | | 21.91 | 0.4 | 55% | |
