# Supplementary material for: Evolution of canonical circadian clock genes underlies unique sleep strategies of marine mammals for secondary aquatic adaptation
Source: PLoS Genet. 2025 Mar 18;21(3):e1011598. doi: 10.1371/journal.pgen.1011598 (PMC11919277; doi:10.1371/journal.pgen.1011598)
Supplement: S13 Table — (DOCX) [file pgen.1011598.s029.docx]

Table S13 Structural alignments for circadian clock proteins from representative species. Rows shaded in grey correspond to the TM-score < 0.5.

|  | **TM-alignment** | | | | **jFATCAT (rigid)** | | | |
| --- | --- | --- | --- | --- | --- | --- | --- | --- |
|  | | **RMSD** | **TM-score** | **Identity** | | **RMSD** | **TM-score** | **Identity** |
| ***BMAL1*** | |  |  |  | |  |  |  |
| *Homo sapiens* | |  |  |  | |  |  |  |
| *Mus musculus* | | 2.73 | 0.92 | 90% | | 2.75 | 0.92 | 89% |
| *Bos taurus* | | 2.57 | 0.92 | 87% | | 3.07 | 0.92 | 88% |
| *Tursiops truncatus* | | 2.29 | 0.94 | 80% | | 2.29 | 0.94 | 80% |
| *Loxodonta africana* | | 4.32 | 0.69 | 67% | | 6.04 | 0.69 | 64% |
| *Trichechus manatus* | | 3.52 | 0.86 | 88% | | 3.94 | 0.86 | 85% |
| *Leptonychotes weddellii* | | 2.42 | 0.92 | 95% | | 3.03 | 0.92 | 96% |
| *Odobenus rosmarus* | | 2.55 | 0.93 | 90% | | 2.56 | 0.93 | 91% |
| ***CLOCK*** | |  |  |  | |  |  |  |
| *Homo sapiens* | |  |  |  | |  |  |  |
| *Mus musculus* | | 5.15 | 0.54 | 79% | | 15.22 | 0.5 | 64% |
| *Bos taurus* | | 5.35 | 0.57 | 78% | | 12.9 | 0.57 | 66% |
| *Tursiops truncatus* | | 7.32 | 0.46 | 77% | | 15.59 | 0.48 | 66% |
| *Loxodonta africana* | | 6.72 | 0.45 | 85% | | 17.02 | 0.41 | 64% |
| *Trichechus manatus* | | 6.24 | 0.47 | 74% | | 16.6 | 0.42 | 68% |
| *Leptonychotes weddellii* | | 4.55 | 0.54 | 80% | | 15.71 | 0.47 | 61% |
| *Odobenus rosmarus* | | 6.42 | 0.48 | 82% | | 16.58 | 0.43 | 55% |
| ***NAPS2*** | |  |  |  | |  |  |  |
| *Homo sapiens* | |  |  |  | |  |  |  |
| *Mus musculus* | | 3.9 | 0.54 | 83% | | 22.18 | 0.16 | 5% |
| *Bos taurus* | | 4.47 | 0.58 | 74% | | 15.64 | 0.57 | 63% |
| *Tursiops truncatus* | | 5.11 | 0.6 | 80% | | 14.78 | 0.52 | 67% |
| *Loxodonta africana* | | 4.55 | 0.55 | 78% | | 15.68 | 0.56 | 61% |
| *Trichechus manatus* | | 5.11 | 0.48 | 70% | | 15.85 | 0.45 | 51% |
| *Leptonychotes weddellii* | | 4.58 | 0.56 | 76% | | 13.4 | 0.51 | 66% |
| *Odobenus rosmarus* | | 4.32 | 0.52 | 85% | | 16.04 | 0.48 | 71% |
| ***CRY1*** | |  |  |  | |  |  |  |
| *Homo sapiens* | |  |  |  | |  |  |  |
| *Mus musculus* | | 1.39 | 0.89 | 95% | | 0.82 | 0.88 | 96% |
| *Bos taurus* | | 2.49 | 0.94 | 88% | | 2.56 | 0.94 | 90% |
| *Tursiops truncatus* | | 1.74 | 0.88 | 98% | | 0.93 | 0.87 | 100% |
| *Loxodonta africana* | | 2.49 | 0.89 | 94% | | 3.73 | 0.88 | 94% |
| *Trichechus manatus* | | 1.46 | 0.89 | 96% | | 1.96 | 0.89 | 93% |
| *Leptonychotes weddellii* | | 1.32 | 0.9 | 96% | | 1.22 | 0.89 | 96% |
| *Odobenus rosmarus* | | 1.42 | 0.89 | 96% | | 1.09 | 0.89 | 97% |
| ***CRY2*** | |  |  |  | |  |  |  |
| *Homo sapiens* | |  |  |  | |  |  |  |
| *Mus musculus* | | 1.56 | 0.87 | 97% | | 5.1 | 0.85 | 96% |
| *Bos taurus* | | 1.57 | 0.85 | 95% | | 6.13 | 0.83 | 91% |
| *Tursiops truncatus* | | 1.38 | 0.87 | 97% | | 3.09 | 0.87 | 97% |
| *Loxodonta africana* | | 1.78 | 0.86 | 95% | | 2.87 | 0.86 | 97% |
| *Trichechus manatus* | | 1.55 | 0.87 | 98% | | 7.6 | 0.78 | 98% |
| *Leptonychotes weddellii* | | 2.17 | 0.87 | 96% | | 4.08 | 0.85 | 91% |
| *Odobenus rosmarus* | | 2.63 | 0.92 | 91% | | 2.88 | 0.91 | 92% |
| ***PER1*** | |  |  |  | |  |  |  |
| *Homo sapiens* | |  |  |  | |  |  |  |
| *Mus musculus* | | 7.55 | 0.57 | 52% | | 14.32 | 0.57 | 59% |
| *Bos taurus* | | 6.78 | 0.55 | 69% | | 14.86 | 0.54 | 67% |
| *Tursiops truncatus* | | 7.86 | 0.43 | 61% | | 19.65 | 0.41 | 46% |
| *Loxodonta africana* | | 7.12 | 0.5 | 55% | | 16.86 | 0.5 | 49% |
| *Trichechus manatus* | | 7.56 | 0.42 | 64% | | 19.51 | 0.41 | 46% |
| *Leptonychotes weddellii* | | 7.83 | 0.52 | 60% | | 16.11 | 0.5 | 53% |
| *Odobenus rosmarus* | | 6.49 | 0.54 | 67% | | 15.93 | 0.54 | 66% |
| ***PER2*** | |  |  |  | |  |  |  |
| *Homo sapiens* | |  |  |  | |  |  |  |
| *Mus musculus* | | 8.21 | 0.45 | 52% | | 17.85 | 0.44 | 44% |
| *Bos taurus* | | 6.95 | 0.56 | 52% | | 13.33 | 0.57 | 42% |
| *Tursiops truncatus* | | 7.49 | 0.54 | 54% | | 13.64 | 0.54 | 49% |
| *Loxodonta africana* | | 6.91 | 0.54 | 54% | | 15.29 | 0.53 | 49% |
| *Trichechus manatus* | | 7.23 | 0.33 | 52% | | 22.23 | 0.25 | 31% |
| *Leptonychotes weddellii* | | 7.38 | 0.53 | 43% | | 13.95 | 0.55 | 39% |
| *Odobenus rosmarus* | | 7.9 | 0.48 | 44% | | 15.42 | 0.5 | 37% |
| ***PER3*** | |  |  |  | |  |  |  |
| *Homo sapiens* | |  |  |  | |  |  |  |
| *Mus musculus* | | 5.91 | 0.35 | 61% | | 25.14 | 0.27 | 32% |
| *Ovis aries* | | 6.79 | 0.45 | 48% | | 16.47 | 0.44 | 43% |
| *Tursiops truncatus* | | 6.4 | 0.35 | 63% | | 21.67 | 0.32 | 38% |
| *Loxodonta africana* | | 7.06 | 0.38 | 56% | | 19.97 | 0.34 | 46% |
| *Trichechus manatus* | | 6.64 | 0.36 | 58% | | 20.91 | 0.36 | 36% |
| *Leptonychotes weddellii* | | 6.77 | 0.4 | 58% | | 17.75 | 0.41 | 39% |
| *Odobenus rosmarus* | | 6.07 | 0.37 | 61% | | 18.86 | 0.35 | 41% |
