# Supplementary material for: Evolution of canonical circadian clock genes underlies unique sleep strategies of marine mammals for secondary aquatic adaptation
Source: PLoS Genet. 2025 Mar 18;21(3):e1011598. doi: 10.1371/journal.pgen.1011598 (PMC11919277; doi:10.1371/journal.pgen.1011598)
Supplement: S14 Table — (DOCX) [file pgen.1011598.s030.docx]

Table S14 Molecular docking results.

| Proteins | Binding energy (Kcal/mol) | Interface area  (Å^2^) | No. of H- bonds |
| --- | --- | --- | --- |
| *Homo sapiens* |  |  |  |
| WT-CLOCK/WT-BMAL1 | -16.0 | 3122.2 | 17 |
| CLOCK-mut/WT-BMAL1 | -43.3 | 4204.2 | 17 |
| WT-CLOCK/ BMAL1-mut | -31.1 | 3879.5 | 11 |
| CLOCK-mut/ BMAL1-mut | -30.7 | 3460.6 | 15 |
| *Mus musculus* |  |  |  |
| WT-CLOCK/WT-BMAL1 | -17.9 | 3332.3 | 6 |
| CLOCK-mut/WT-BMAL1 | -32.4 | 4177.8 | 16 |
| WT-CLOCK/ BMAL1-mut | -37.9 | 4939.8 | 14 |
| CLOCK-mut/ BMAL1-mut | -27.1 | 4057.8 | 21 |
| *Bos taurus* |  |  |  |
| WT-CLOCK/WT-BMAL1 | -11.5 | 3688.3 | 10 |
| CLOCK-mut/WT-BMAL1 | -30.7 | 4633.5 | 21 |
| WT-CLOCK/ BMAL1-mut | -24.5 | 3729.5 | 16 |
| CLOCK-mut/ BMAL1-mut | -38.6 | 4476.0 | 12 |
| *Tursiops truncatus* |  |  |  |
| WT-CLOCK/WT-BMAL1 | -37.5 | 3860.1 | 13 |
| CLOCK-mut/WT-BMAL1 | -22.8 | 3408.1 | 12 |
| WT-CLOCK/ BMAL1-mut | -32.3 | 3417.7 | 10 |
| CLOCK-mut/ BMAL1-mut | -31.9 | 3707.3 | 9 |
