# Supplementary material for: Evolution of canonical circadian clock genes underlies unique sleep strategies of marine mammals for secondary aquatic adaptation
Source: PLoS Genet. 2025 Mar 18;21(3):e1011598. doi: 10.1371/journal.pgen.1011598 (PMC11919277; doi:10.1371/journal.pgen.1011598)
Supplement: S15 Table — (DOCX) [file pgen.1011598.s031.docx]

Table S15 Detail information on sample collection for PCR amplification and sequencing.

| Scientific name | Sample ID | Sex | Body length  (cm) | Body weight  (Kg) | Date | Location | Tissue type |
| --- | --- | --- | --- | --- | --- | --- | --- |
| *Stenella coeruleoalba* | 2013056 | Male | 210 | - | November 24st, 2023 | Beihai, Guangxi, China | Muscle, Liver |
|  | 2013057 | Male | 220 | - | November 25st, 2013 | Beihai, Guangxi, China | Muscle, Liver |
| *Delphinus delphis* | 7807 | Male | 205 | 75 | June 13st, 1978 | Taichang, Jiangsu, China | Muscle, Liver |
| *Tursiops aduncus* | 200624 | Male | 234 | 133.5 | March 17st, 2006 | Xiamen, Fujian, China | Muscle |
|  | 200915 | - | - | - | April 17st, 2009 | Xiamen, Fujian, China | Blood |
|  | 2013038 | - | - | - | June 15st, 2013 | Nanning, Guangxi, China | Blood |
| *Sousa chinensis* | 7805 | Female | 237 | 161.5 | March 17st, 1978 | Zhangzhou, Fujian, China | Liver |
|  | 9501 | Female | 229 | 140 | February 17st, 1995 | Yueqing, Zhejiang, China | Muscle, |
| *Grampus griseus* | 9821 | - | 348 | - | December 6th, 1998 | Dongshan, Fujian, China | Muscle |
| *Delphinapterus leucas* | 200942 | - | - | - | May 21st, 2009 | Penglai, Shandong, China | Blood |
|  | 200943 | - | - | - | May 21st, 2009 | Penglai, Shandong, China | Blood |
| *Mesoplodon densirostris* | 2021001 | Male | 412 | - | March 7th 2020 | Wenzhou, Zhejiang, China | Muscle, Liver |
| *Kogia sima* | 200893 | - | - | - | June 4th 2008 | Pingtan, Fujian, China | Blood |
| *Balaenoptera omurai* | 2019008 | Male | 714 | 2,500 | January 24st, 2019 | Ningbo, Zhejiang, China | Muscle, Liver |
